# A Copper-Catalyzed Tandem Cyclization Reaction of Aminoalkynes with Alkynes for the Construction of Tetrahydropyrrolo[1,2-a]quinolines Scaffold

**DOI:** 10.1038/s41598-017-16887-0

**Published:** 2017-11-30

**Authors:** Can-Liang Ma, Jin-Hao Zhao, Yong Yang, Min-Kui Zhang, Chao Shen, Rong Sheng, Xiao-Wu Dong, Yong-Zhou Hu

**Affiliations:** 1 Zhejiang Province Key Laboratory of Anti-Cancer Drug Research, College of Pharmaceutical Sciences, Zhejiang University, Hangzhou, 310058 China; 2 Ministry of Agriculture Key Laboratory of Molecular Biology of Crop Pathogens and Insects, Institute of Pesticide and Environmental Toxicology, Zhejiang University, Hangzhou, 310029 China

## Abstract

A synthetic method for diversely substituted tetrahydropyrrolo[1,2-*a*]quinolines was developed via CuCl-catalyzed cascade transformation of internal aminoalkynes with alkynes under microwave- irradiation.

## Introduction

**Figure 1:**
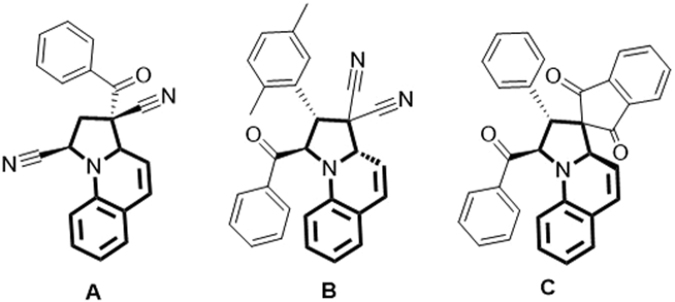
Selected biologically active molecules containing the tetrahydropyrrolo[1,2-*a*]quinoline scaffold.

The substituted tetrahydropyrrolo[1,2-*a*]quinoline scaffold is found in a variety of biologically active compounds. For example, the treatment of cells with compound **A** (Fig. 1) results in reduced sensitivity to the toxicity of anthrax^1^. In addition, compound **B** exhibits potent malarial cysteine protease inhibitory activity^2^ and compound **C** was regarded as a selective Aurora B-INCENP interaction inhibitor^3^.

**Figure 2:**
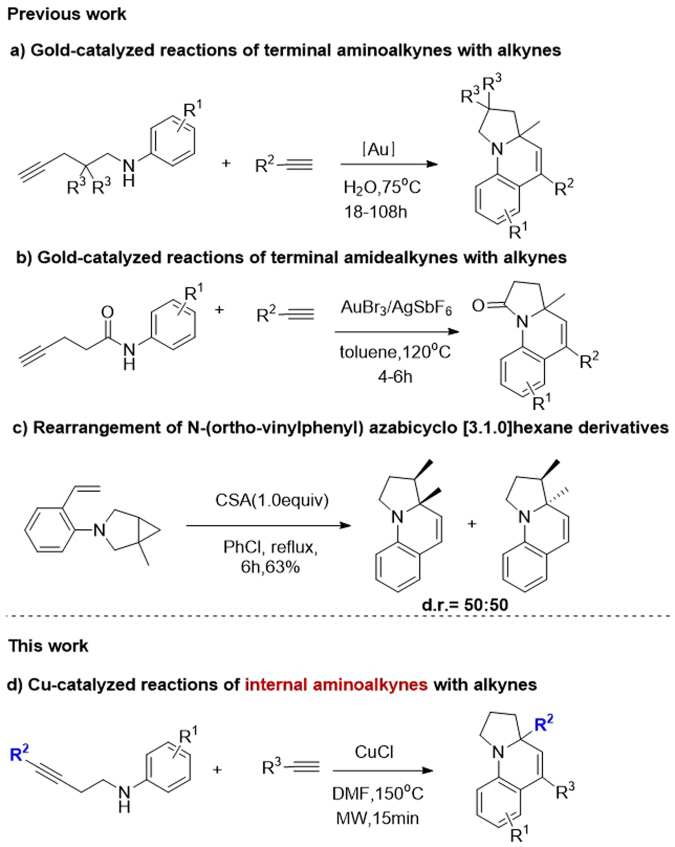
Synthesis of tetrahydropyrrolo[1,2-*a*] quinolines.

In the past few years, several synthetic methods have been explored on the construction of the tetrahydropyrrolo-[1,2-*a*]quinoline scaffold^4–6^. In particular, Liu *et al*. developed a gold catalyzed tandem reaction using terminal aminoalkynes and alkynes as starting material, which can establish the polyheterocycle scaffold simultaneously under mild reaction conditions (Fig. 2a)^4^. Consequently, Zhou *et al*. extended this reaction using less active terminal amidoalkynes and alkynes with similar conditions (Fig. 2b)^5^. In these reactions, only terminal aminoalkynes or amidoalkynes were used as substrates, and expensive gold catalysts were required with long reaction times, these factors restrict the exploration of larger panels of substituted tetrahydropyrrolo[1,2-*a*]quinoline substrates. Recently, Wasilewska *et al*. reported the rearrangement of *N*-(*ortho*-vinylphenyl) azabicyclo-[3.1.0]hexane derivatives to obtain the target compounds. However, the tedious procedure to prepare starting materials also restricts its widespread application (Fig. 2c)^6^.

The multiple biological activities of tetrahydropyrrolo-[1,2-*a*]quinoline attracted our interest and we try to search for a more convenient and efficient method. In 2010, Han *et al*. reported the Cu(I) catalyzed intramolecular amination of internal aminoalkynes to prepare *N*-heterocycles under microwave irradiation conditions^7^, where the copper catalyst was more tolerant towards basic amines than gold catalysts for Snogashira-type reactions. Inspired by this discovery, we envisioned that the tetrahydropyrrolo-[1,2-*a*] quinolines could be produced via a Cu(I) catalyzed tandem reaction using internal aminoalkynes and alkynes (Fig. 2d).

## Results and Discussion

**Figure 3:**
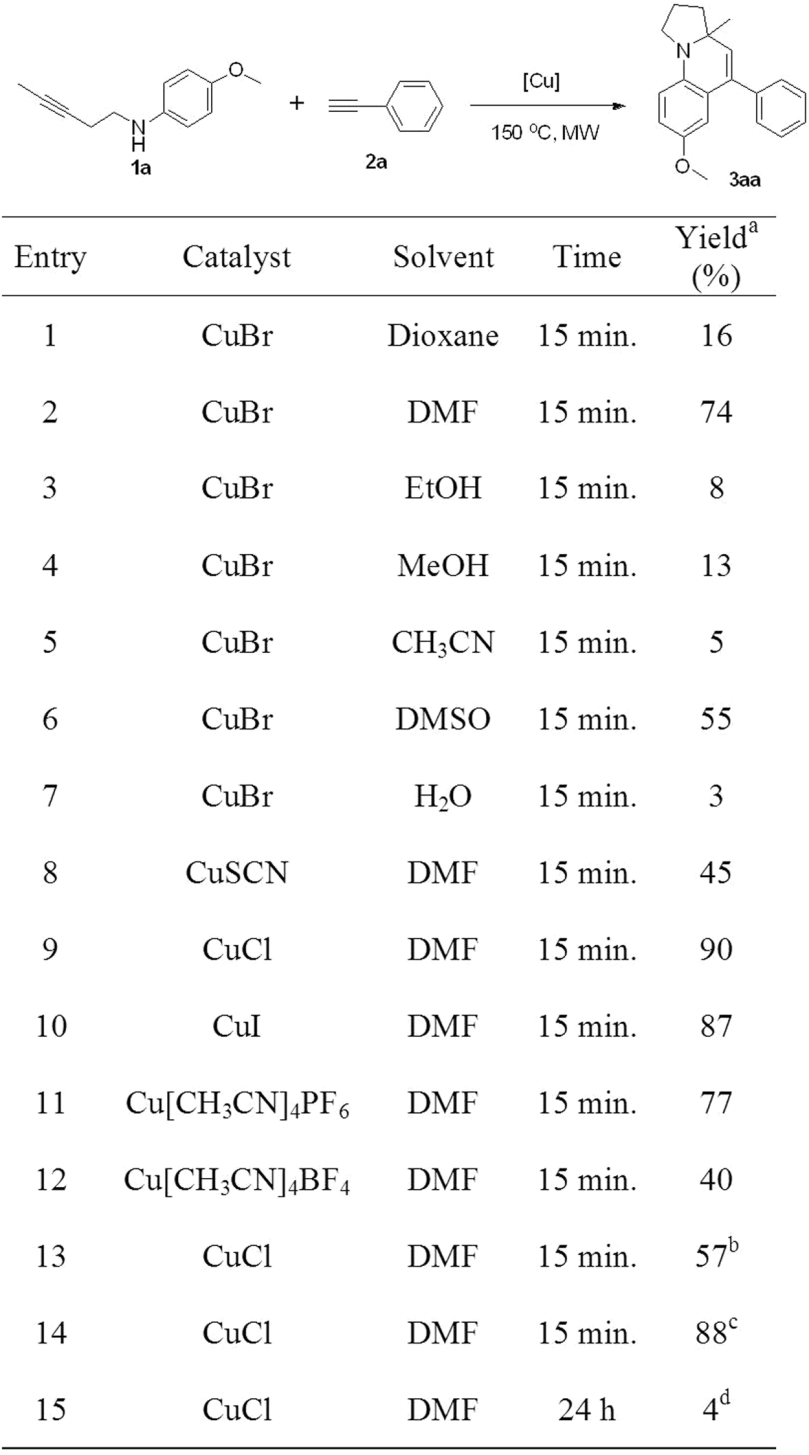
Screening of reaction conditions.

To validate the feasibility of the proposed process, aminoalkyne (**1a**) and phenylacetylene (**2a**) were chosen as the starting materials with different catalytic reaction conditions. Firstly, we tried the similar condition as Han’s method, in whose catalytic system CuBr can increase the reaction rate^7^. Thus, CuBr (10 mol %) was used as catalyst in dioxane under MW irradiation at 150 °C. To our delight, the desired product (**3aa**) can be produced with a relatively low yield of 16% (Fig. 3, Entry 1). Intriguingly, the replacement of dioxane with DMF and DMSO led to a remarkable increase in yield of desired product (74% and 55%, Entry 2 and 6), probably due to its excellent polarity and good stability under high temperature. Further screening of polar solvents (ethanol, methanol and water, Entry 3,4,7) revealed that protonic solvent ethanol, methanol and acetonitrilewere not suitable for this reaction. Additionally, the use of acetonitrile (Entry 3–5) also only produced trace of target compound (5%, Entry 7), maybe owing to the poor solubility of CuBr in thisese solvent. Subsequently, different copper catalysts were investigated, and the data showed that CuSCN, CuI, Cu[CH_3_CN]_4_PF_4_ and Cu[CH_3_CN]_4_BF_4_ provided moderate to good yields of **3aa** (40–87%, Entry 8, 10–12), CuCl displayed the most potent catalytic efficiency with a yield of 90% (Entry 9). Further modification of the ratio of **1a** to **2a** revealed that 1:3 was optimal (Entry 9, 13–14). To confirm the use of MW irradiation is necessary or not in this reaction^8–10^, we also tried the reaction without microwave irradiation, only affording the desired product with 4% yield (Entry 15). Therefore, the optimal conditions for this copper catalyzed reaction between internal aminoalkynes and alkynes is CuCl (10 mol %), in DMF under microwave irradiation at 150 °C for 15 minutes.

**Figure 4:**
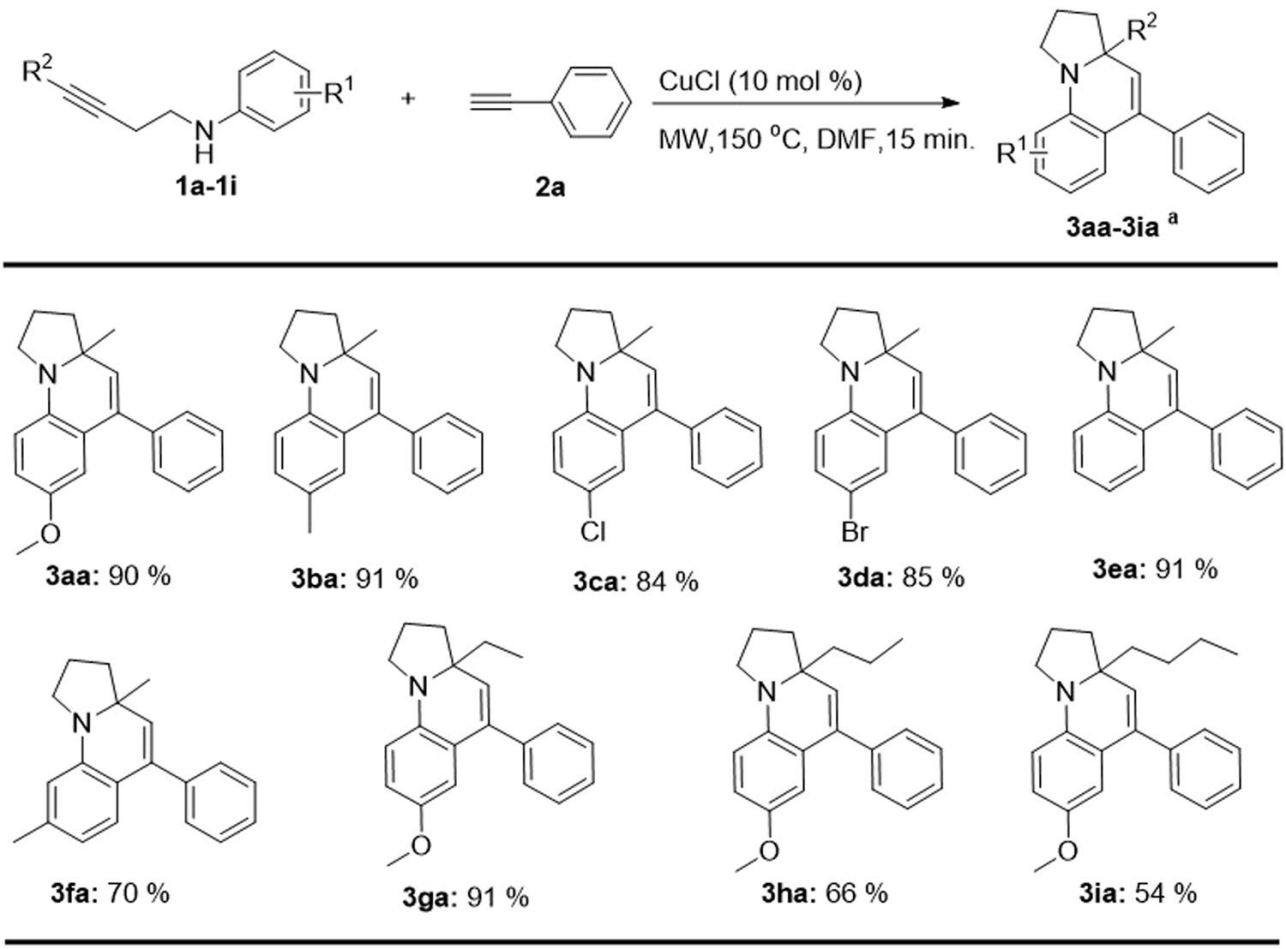
The scope of aminoalkynes 1. Reaction conditions: 1a (0.1 mmol), 2 (0.3 mmol), catalyst (10 mol %), DMF (3.0 mL), at the corresponding temperature under argon. ^a^Isolated yields are shown.

Next, the substrate scope of aminoalkynes **1** was investigated. As shown in Fig. 4, both products with electron-donating groups (**3aa**, **3ba**) and electron-withdrawing groups (**3ca**, **3da**) were obtained in excellent yield (84–91%), indicating the electronic effect was of little influence on this tandem reaction.

Reaction conditions: **1a** (0.1 mmol), **2a** (0.3 mmol), catalyst (10 mol %), solvent (3.0 mL), at the corresponding temperature under argon. ^a)^Determined by ^1^H NMR spectroscopy using CH_2_Br_2_ as internal standard. ^b)^ **1a** (0.1 mmol), **2a** (0.2 mmol). ^c)^ **1a** (0.1 mmol), **2a** (0.4 mmol). ^d)^The reaction was carried out without microwave irradiation.

Then, the effect of the substituent position in compound **1** on this tandem cyclization reaction was investigated. When 3-methyl-*N*-(pent-3-yn-1-yl)-aniline **1f** was used as the substrate, cyclization was observed at the 6-position to produce **3fe** in 70% yield. Although there were two possible positions where cyclization could occur with the alkyne moiety (2- and 6-position), none of the product with cyclization at the 2-position was detected. These results show that good regio-selectivity was achieved in this tandem cyclization, probably due to the steric hindrance effect. In addition, aminoalkynes with different R^2^ (ethyl, propyl and butyl) can also perform this reaction smoothly to give **3ga**, **3ha** and **3ia** with yields ranging from 54% to 91%, suggesting that this method can be used for various aminoalkynes.

**Figure 5:**
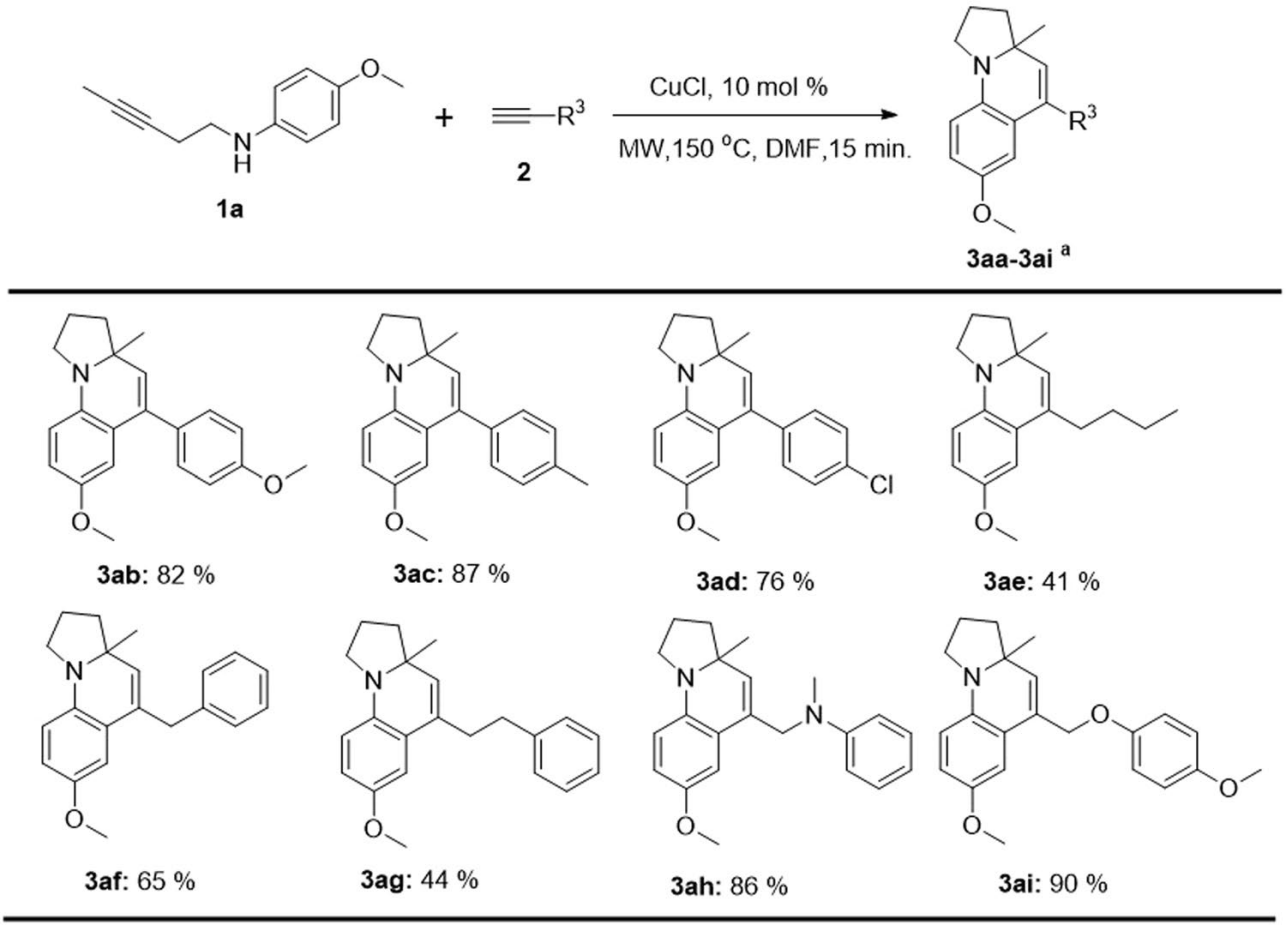
The scope of alkyne 2. Reaction conditions: 1a (0.1 mmol), 2 (0.3 mmol), catalyst (10 mol %), DMF (3.0 mL), at the corresponding temperature under argon. ^a^Isolated yields are shown.

We have also investigated the substrate scope of the alkynes **2**, including the presence of electron-donating groups (EDG) and electron-withdrawing groups (EWG) substituted phenylacetylenes (**2a-2d**), hexyne (**2e**), 3-phenyl-1-propyne (**2 f**), 4-phenyl-1-butyne (**2g**), *N*-methyl-*N*-(prop-2-yn-1-yl)aniline (**2 h**) and 4-(prop-2-yn-1-yloxy) anisole (**2i**). The data in Fig. 5 shows that all these alkynes can undergo this tandem cyclization reaction smoothly with moderate to good yields (41–90%).

**Figure 6:**
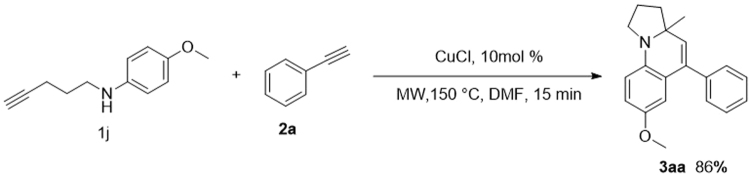
The reaction of terminal aminoalkyne 1 and alkyne 2a under optimized conditions.

With the successful tandem cyclization of internal aminoalkynes with alkynes, we postulated the terminal aminoalkynes also could carry out this reaction with our method. The reaction between 4-methoxy-N-(pent-4-yn-1-yl)-aniline and phenylethylene under optimized condition also gave desired product **3aa** in excellent yield (Fig. 6, 86%), which confirmed that both terminal aminoalkynes and internal aminoalkynes are suitable for this tandem reaction. Therefore, our method provides a more efficient and feasible choice for the construction of the tetrahydropyrrolo[1,2-*a*]quinoline scaffold than those previously reported^4^.

**Figure 7:**
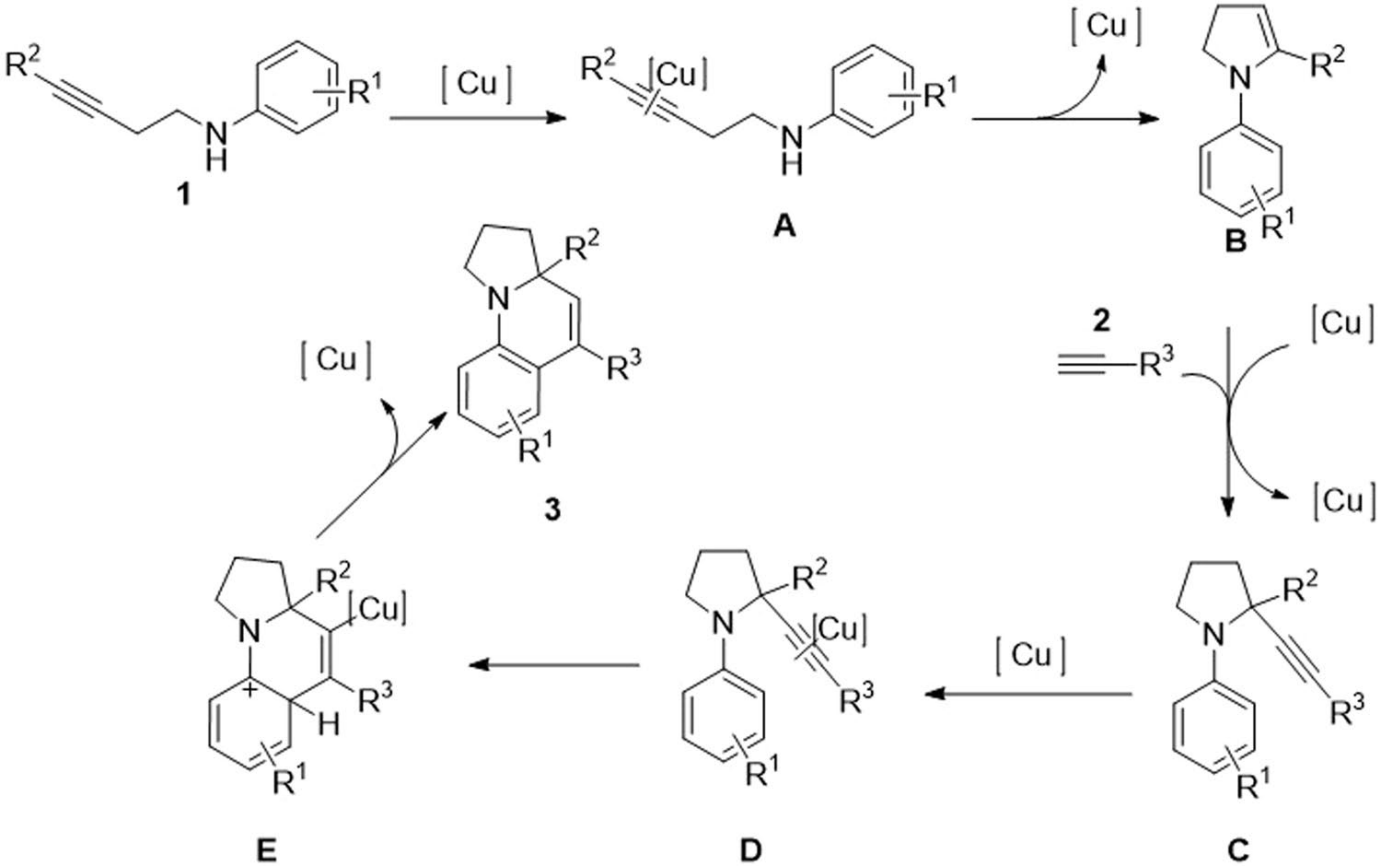
Proposed mechanism for copper-catalyzed tandem cyclization.

Through examination of the reaction conditions, a possible mechanism of the copper-catalyzed tandem cyclization reaction was proposed in Fig. 7. Similar to that of the gold-catalyzed reaction^4,5^, the internal aminoalkyne **1** is first activated by the Cu catalyst to generate intermediate **A**, which is then converted to enamine intermediate B via intramolecular hydroamination. Then, alkyne **2** reacted with anamine **B** to provide propargylamine **C**, which was cyclized intramolecularly to give product **3** in the presence of copper catalyst. The mechanism is also supported by reported reference, in where the intermediate similar to compound **C** was synthesized and cyclized to target compound via copper catalysis^11^.

**Figure 8:**
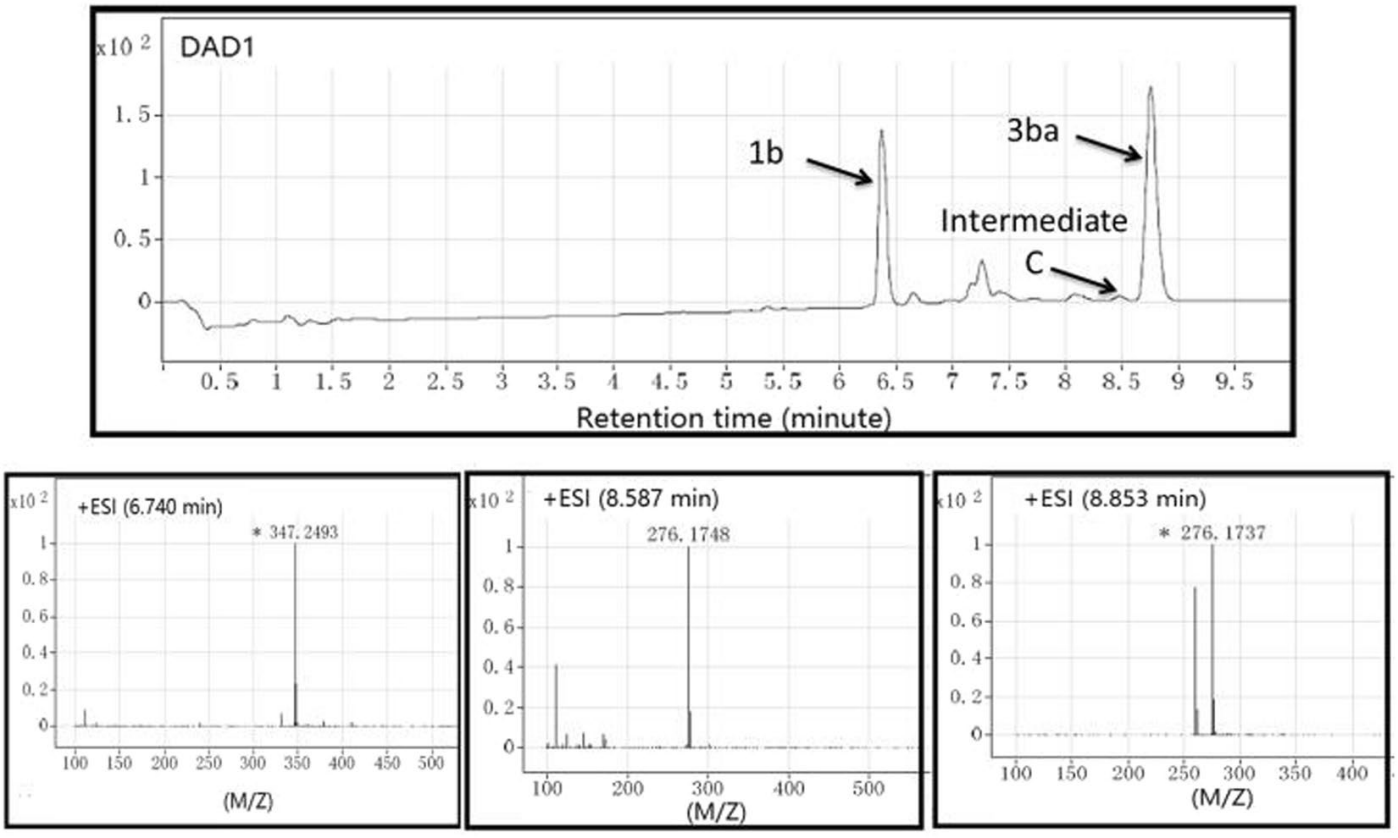
LC-HRMS analysis of the reaction mixture of **1b** and **2a** to identify key intermediate **C**.

In order to support the proposed mechanism, we performed LC-HRMS analysis of the reaction mixture of **1b** and **2a** in DMF after stirring for 10 min at 150 °C under microwave irradiation^12–14^. Three peaks were detected with m/z = 347.2493, 276.1748, 276.1737 at retention times of 6.47, 8.47 and 8.87 min, respectively. The first is the [2 M + H]^+^ of the starting material **1b**, the last peak is confirmed as **3ba** by comparison with the obtained product, and the middle peak can be attributed to the intermediate **C** (Fig. 8). In addition, we performed reaction of **1b** and **2a** in DMF after stirring for 2 h at 150 °C and isolated the intermediate C. (The structure of C was confirmed by ^1^H NMR spectra, see SI).

**Figure 9:**
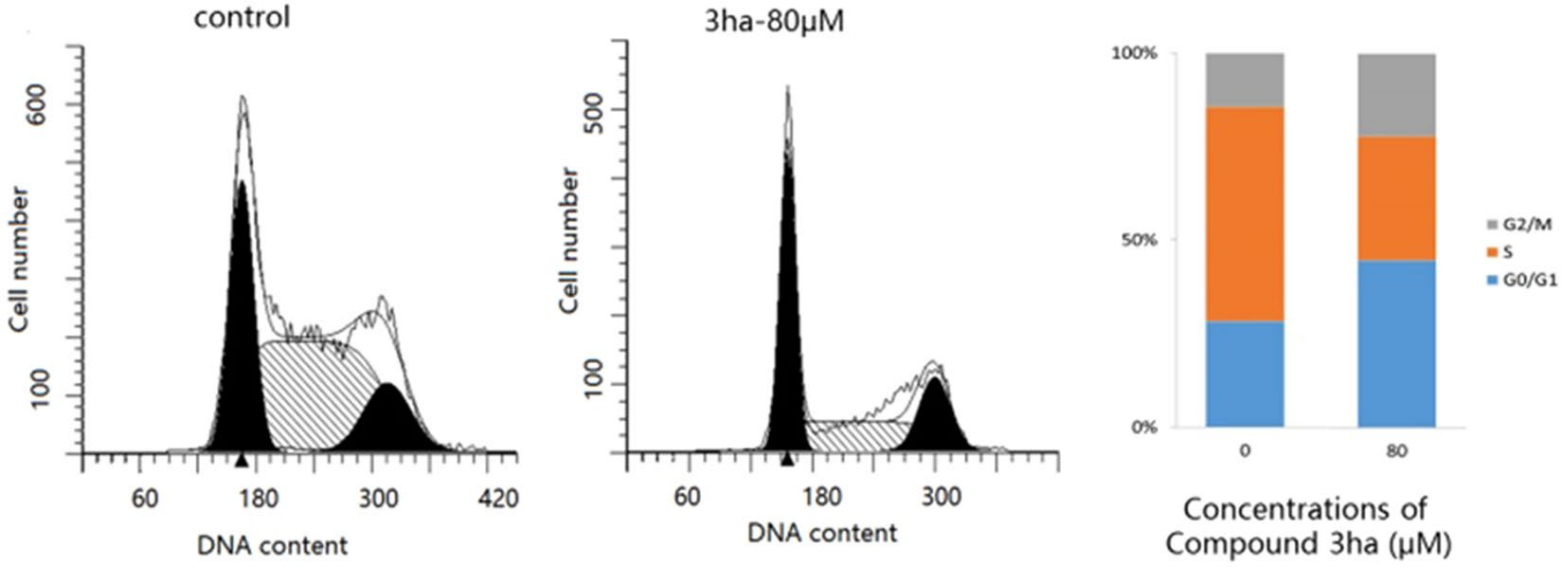
Cell cycle analysis. Cell cycle distribution upon treatment of **3ha** varies between capan-2 cell lines. The cell cycle distribution of capan-2 cells was determined by flow cytometry.

Moreover, these synthesized tetrahydropyrrolo[1,2-*a*]quinolines were submitted to biologically evalution in in various phenotypic screening. To be of interest, several of them demonstrated cytotoxic activity against human pancreatic cancer cell CFPAC1 and CAPAN2 *in vitro* (see the Supporting Information, Table S1). For example, compound **3ha** showed an inhibition ratio of 68.2% at 100 µM against pancreatic cancer cell capan-2 proliferation *in vitro*. Further cell cycle analysis using different concentrations of **3ha** showed that the percentage of capan-2 cells in G0/G1 phase (44.63%) treated with compound **3ha** (80 µM) was significantly higher than that of control (28.39%) (Fig. 9), indicating the growth of capan-2 cells can be arrested by compound **3ha** at G0/G1 phase in various concentrations. (see the Supporting Information, Figure S1).

## Conclusions

In summary, using a cheap catalyst CuCl and easily available starting materials, we developed a convenient and efficient method for the synthesis of diversely substituted tetrahydropyrrolo[1,2-*a*]quinolines with excellent regio- and chemoselectivity. Further studies, including asymmetric variation of this tandem reaction and extensive biological evaluations on tetrahydropyrrolo[1,2-*a*]quinolines are currently underway in our laboratory.

## Experimental Section

### General procedure for the synthesis of diversely substituted tetrahydropyrrolo[1,2-a]quinolines under microwave irradiation

To a 5 mL Biotage Microwave vial equipped with a magnetic stir bar, CuCl (0.0100 mmol), aminoalkyne **1** (0.100 mmol), alkyne **2** (0.300 mmol) and DMF (3.00 mL) were added. The resulting mixture in sealed vial was stirred at 150 °C under microwave irradiation for 15 min, and water (10.0 mL) was added to the vial to quench the reaction. The mixture was extracted with AcOEt (3 × 10.0 mL) and the combined organic layers was washed with small amounts of water (5 × 5.00 mL) and dried with Na_2_SO_4_. The solvent was evaporated in vacuo and the residue was purified by silica gel column chromatography using n-hexane/EA as eluent to give the desired products.

### Cell Viability assay

Cells were counted in logarithmic phase and 5,000 cells were placed in 96-well plates. After treatment with compounds (0.4, 20 and 100 μM), cells were incubated for an additional 2 h with CCK-8 reagent (100 μL/mL medium) and the absorbance was read at 450 nm using a microplate reader (Sunnyvale, CA, USA). Cell proliferation inhibition rates were calculated according to the following formula: the proliferation inhibition ratio (%) = 1 − [(A1 − A3)/(A2 − A3)] × 100, where, A1 is the OD value of drug experimental group, A2 is the OD value of blank control group, A3 is the OD value of the RPMI1640 medium without cells. Assays were performed on three independent experiments.

### Apoptosis assay by flow cytometry

Exponentially growing cells were seeded in 6-well plates (5 × 10^4^/well) and cultured overnight in a 5% CO_2_ atmosphere at 37 °C. After treatment with **3ha/3ah/**DMSO for 24 h, cells were harvested and washed with PBS. Then cells were stained with Annexin V-FITC Apoptosis Kit according to the manufacturer’s instructions and analyzed by flow cytometry (Becton Dickinson, Franklin Lakes, NJ, US). Assays were performed on three independent experiments.

## Electronic supplementary material

Supplementary Information
