# Supplementary material for: A Copper-Catalyzed Tandem Cyclization Reaction of Aminoalkynes with Alkynes for the Construction of Tetrahydropyrrolo[1,2-a]quinolines Scaffold
Source: Sci Rep. 2017 Nov 30;7:16640. doi: 10.1038/s41598-017-16887-0 (PMC5709353; doi:10.1038/s41598-017-16887-0)

**A Copper-Catalyzed Tandem Cyclization Reaction of Aminoalkynes with Alkynes for the Construction of Tetrahydropyrrolo[1,2-a]quinolines Scaffold**

Can-Liang Ma^1,+^,Jin-Hao Zhao^2,+^, Yong Yang^2^, Min-Kui Zhang^1^ , Chao Shen^1^, Rong Sheng^1,*^, Xiao-Wu Dong^1,*^, Yong-Zhou Hu^1,*^

^1^ Zhejiang Province Key Laboratory of Anti-Cancer Drug Research, College of Pharmaceutical Sciences, Zhejiang University, Hangzhou, 310058, China

^2^ Ministry of Agriculture Key Laboratory of Molecular Biology of Crop Pathogens and Insects, Institute of Pesticide and Environmental Toxicology, Zhejiang University, Hangzhou, 310029, China.

* Corresponding authors: (Rong Sheng, E-mail: [shengr@zju.edu.cn](mailto:shengr@zju.edu.cn); Xiaowu Dong, E-mail: dongxw@zju.edu.cn; Yongzhou Hu, E-mail: [huyz@zju.edu.cn](mailto:huyz@zju.edu.cn))

^+^ These authors contributed equally to this work

**Table of Contents**

**General information………………………………………………………………S2**

**Synthesis of starting materials……………………………………………………S2**

**General procedure for Cu-catalyzed Synthesis of Diversely Substituted tetrahrdropyrrolo[1,2-a]quinolines………………………………………………S6**

**Table S1……………………..……………………………………………………..S13**

**Figure S1……………………………………….………………………………….S14**

**Spectra…………………………………………………………………….……….S15**

**Experimental Section**

**General Methods.** All manipulations with air-sensitive reagents were carried out under a dry argon atmosphere. Unless otherwise stated, all commercial reagents were used without additional purification. Solvents were dried using standard methods and distilled before use. Analytical thin layer chromatography (TLC) was performed on percolated silica gel 60 F254 plates. Visualization on TLC was achieved by use of UV light (254 nm) or iodine. NMR spectra were recorded on a Bruker DPX-500 spectrometer at 500/600 MHz for ^1^H NMR, 125/150 MHz for ^13^C NMR in CDCl_3_ with tetramethylsilane (TMS) as internal standard. The chemical shifts are expressed in ppm and coupling constants are given in Hz. Data for ^1^H NMR are recorded as follows: chemical shift (ppm), multiplicity (s, singlet; d, doublet; t, triplet; q, quarter; m, multiplet), coupling constant (Hz), integration. Data for ^13^C NMR are reported in terms of chemical shift (δ, ppm). Mass spectra were determined on a Finnigan MAT 95 mass spectrometer.

**Synthesis of starting materials**

Aminoalkyne **1a-i** were synthesized through the below procedure

To a solution of 3-butyn-1-ol **A** (5.00 g, 72.0 mmol) and imidazole (6.80 g, 100 mmol) in CH_2_Cl_2_ (100 mL) was added TBSCl (12.9 g, 86.0 mmol) at 0 ˚C and stirred at rt for 30 min. The reaction mixture was quenched with H_2_O and extracted with CH_2_Cl_2_, the combined organic layer was washed with brine, dried over MgSO_4_, filtered and concentrated in vacuo to give a crude product, which was purified by silica gel column chromatography (AcOEt/ hexane = 1/ 9) to afford 11.6 g of **B** as a colorless oil with a yield of 92 %.

To a solution of the above obtained alkyne **B** (291 mg, 1.25 mmol) in dry THF (8.00 mL) at -78 °C was added n-BuLi (1.10 mL, 1.60 M in hexane, 1.76 mmol) dropwise via syringe and the mixture was stirred for 20 min. Iodohydrocarbon (2.17 mmol) was added via syringe and the mixture was allowed to warm to 0 °C over 1.5 h. It was then warmed to ambient temperature and stirred for 30 min after which the reaction was quenched with water (20.0 mL). The mixture was extracted with Et_2_O (3 × 20.0 mL). The combined organic layers were dried over Na_2_SO_4_, filtered, and evaporated in vacuo at -5 °C. The residue was passed through a short plug of silica eluted with hexane to give the compound **C** as a clear colorless oil with a yield of 57 %.

To a solution of the above obtained alkyne **C** (1.00 g, 3.00 mmol) in anhydrous THF (9.00 mL) cooled at 0 °C was added TBAF/THF (3.60 mL, 3.60 mmol ). After 1 h the reaction mixture was concentrated and the crude compound was purified by column chromatography using EtOAc–hexane (1 : 9, v/v) to afford pure hydroxyalkyne **D** as a colorless oil with a yield of 89 %.

To a solution of hydroxyalkyne **D** (15.8 mmol), triethylamine (2.70 mL, 19.0 mmol), and 4- (dimethylamino) pyridine (39.0 mg, 0.320 mmol) in DCM (53.0 mL) at 0 ^o^C was added p-toluenesulfonyl chloride (3.16 g, 16.6 mmol) in three portions. The reaction mixture was brought to rt and stirred for 15 h. Aqeaous NaOH (1.00 mol/L, 30.0 mL) was added to the mixture and vigorously stirred for 15 min at r.t. Then, the mixture was extracted with DCM, washed with brine, and removal of the solvent gave p-toluenesulfonate **E** as yellow oil with a yield of 93 %.

To a solution of substituted aniline (3.00 mmol, 1.50 eq.), p-toluenesulfonate **E** (2.00 mmol, 1.00 eq) and KI (33.0 mg, 0.200 mmol, 0.100 eq.) in DMF (4.00 mL) was added K_2_CO_3_ (818 mg, 6.00 mmol, 3.00 eq.), and the mixture was heated to 90 °C. After the complete consumption of the p-toluenesulfonate **E** (monitered by TLC), the reaction mixture was cooled to room temperature and then quenched with a saturated solution of NH_4_Cl, extracted with AcOEt (3×10.0 mL). The combinded organic layer was washed with small amounts of water (5×5.00 mL) and dried over Na_2_SO_4_. The solvent was removed under reduced pressure and the residue was purified by flash column chromatography to afford the desired compound **1a-1i** as yellow oil with yields from 47 % to 79 %.

Yellow oil; ^1^H NMR (500 MHz, CDCl_3_) δ 6.82 (d, *J* = 8.5 Hz, 2H), 6.66 (d, *J* = 8.5 Hz, 2H), 3.78 (s, 2H), 3.23 (t, *J* = 6.5 Hz, 2H), 2.49 – 2.43 (m, 2H), 1.83 (t, *J* = 2.5 Hz, 3H). ^13^C NMR (125 MHz, CDCl_3_) δ 152.4, 142.0, 114.9, 114.7, 77.4, 76.5, 55.8, 44.1, 19.5, 3.5. IR(KBr): ν 3060, 2922, 2831, 1618, 1513, 1464, 1239, 820 cm^-1^.ESI-MS: m/z = 190 [M+H]^+^.

Yellow oil; ^1^H NMR (500 MHz, CDCl_3_) δ 7.04 (d, *J* = 8.5 Hz, 2H), 6.61 (d, *J* = 8.5 Hz, 2H), 3.82 (s, 1H), 3.26 (t, *J* = 6.5 Hz, 2H), 2.50 – 2.45 (m, 2H), 2.29 (s, 3H), 1.85 (t, *J* = 2.5 Hz, 3H). ^13^C NMR (125 MHz, CDCl_3_) δ 145.6, 129.7, 126.9, 113.3, 77.3, 76.4, 43.3, 20.3, 19.4, 3.5. IR(KBr): ν 3095, 2925, 2853, 1599, 1503, 1431, 815 cm^-1^.ESI-MS: m/z = 174 [M+H]^+^.

Yellow oil; ^1^H NMR (500 MHz, CDCl_3_) δ 7.12 (d, *J* = 9.0 Hz, 2H), 6.55 (d, *J* = 9.0 Hz, 2H), 3.92 (s, 1H), 3.21 (t, *J* = 6.5 Hz, 2H), 2.51 – 2.36 (m, 2H), 1.80 (t, *J* = 2.5 Hz, 3H). ^13^C NMR (125 MHz, CDCl_3_) δ 146.4, 129.1, 122.2, 114.2, 77.7, 76.1, 43.1, 19.4, 3.5. IR(KBr): ν 3026, 2922, 2851, 1600, 1504, 1261, 815, 730 cm^-1^. ESI-MS: m/z = 194 [M+H]^+^.

Yellow oil; ^1^H NMR (500 MHz, CDCl_3_) δ 7.28 (d, *J* = 9.0 Hz, 2H), 6.53 (d, *J* = 9.0 Hz, 2H), 3.97 (s, 1H), 3.25-3.19 (m, 2H), 2.53 – 2.41 (m, 2H), 1.83 (t, *J* = 2.5 Hz, 3H). ^13^C NMR (125 MHz, CDCl_3_) δ 146.8, 131.9, 114.7, 109.3, 77. 7, 76.1, 42.9, 19.3, 3.5. IR(KBr): ν 3026, 2922, 2852, 1596, 1558, 1495, 1261, 813, cm^-1^. ESI-MS: m/z = 238 [M+H]^+^.

Yellow oil; ^1^H NMR (500 MHz, CDCl_3_) δ 7.26 – 7.19 (m, 2H), 6.76 (t, *J* = 7.5 Hz, 1H), 6.70 – 6.65 (m, 2H), 3.97 (s, 1H), 3.28 (t, *J* = 6.5 Hz, 2H), 2.55 – 2.41 (m, 2H), 1.85 (t, *J* = 2.5 Hz, 3H). ^13^C NMR (125 MHz, CDCl_3_) δ 147.9, 129.3, 117.7, 113.1, 77.4, 76.4, 43.0, 19.5, 3.5. IR(KBr): ν 3025, 2922, 1635, 1558, 1196 cm^-1^. ESI-MS: m/z = 160 [M+H]^+^.

Yellow oil; ^1^H NMR (500 MHz, CDCl_3_) δ 7.11 (t, *J* = 7.5 Hz, 1H), 6.58 (d, *J* = 7.5 Hz, 1H), 6.51-6.47 (m, 2H), 3.89 (s, 1H), 3.27 (t, *J* = 6.5 Hz, 2H), 2.47 (tq, *J* = 6.5, 2.5 Hz, 2H), 2.32 (s, 3H), 1.84 (t, *J* = 2.5 Hz, 3H). ^13^C NMR (125 MHz, CDCl_3_) δ 148.0, 139.0, 129.1, 118.6, 113.9, 110.2, 77.4, 76.5, 43.0, 21.6, 19.5, 3.5. IR(KBr): ν 3025, 2922, 2852, 1605, 1586, 1509, 1209, 810 cm^-1^. ESI-MS: m/z = 174 [M+H]^+^.

Yellow oil; ^1^H NMR (500 MHz, CDCl_3_) δ 6.82 (d, *J* = 9.0 Hz, 2H), 6.65 (d, *J* = 9.0 Hz, 2H), 3.78 (s, 3H), 3.23 (t, *J* = 6.5 Hz, 2H), 2.48 (tt, *J* = 6.5, 2.5 Hz, 2H), 2.24-2.18 (m, 2H), 1.16 (t, *J* = 7.5 Hz, 3H). ^13^C NMR (125 MHz, CDCl_3_) δ 152.42, 142.1, 117.4, 114.9, 114.7, 83.6, 76.6, 55.8, 44.1, 19.5, 14.3, 12.4. IR(KBr): ν 3056, 2974, 2832, 1513, 1238, 820 cm^-1^. ESI-MS: m/z = 204 [M+H]^+^.

Yellow oil; ^1^H NMR (500 MHz, CDCl_3_) δ 6.82 (d, *J* = 9.0 Hz, 2H), 6.66 (d, *J* = 9.0 Hz, 2H), 3.78 (s, 3H), 3.23 (t, *J* = 6.5 Hz, 2H), 2.49 (tt, *J* = 6.5, 2.5 Hz, 2H), 2.18 (tt, *J* = 6.5, 2.5 Hz, 2H), 1.59-1.47 (m, 2H), 1.01 (t, *J* = 7.5 Hz, 3H). ^13^C NMR (125 MHz, CDCl_3_) δ 152.5, 141.9, 114.9, 114.8, 82.2, 77.4, 55.8, 44.2, 22.4, 20.8, 19.5, 13.5. IR(KBr): ν 3025, 2922, 2832, 1509, 1243 cm^-1^. ESI-MS: m/z = 218 [M+H]^+^.

Yellow oil; ^1^H NMR (600 MHz, CDCl_3_) δ 6.80 (d, *J* = 9.0 Hz, 2H), 6.62 (d, *J* = 9.0 Hz, 2H), 3.76 (s, 3H), 3.71 (s, 1H), 3.21 (t, *J* = 6.6 Hz, 2H), 2.48 – 2.42 (m, 2H), 2.21-2.17 (m, 2H), 1.53 – 1.47 (m, 2H), 1.46-1.39 (m, 2H), 0.94 (t, *J* = 7.2 Hz, 3H). ^13^C NMR (150 MHz, CDCl_3_) δ 152.4, 142.2, 114.9, 114.6, 82.2, 77.3, 55.8, 44.1, 31.1, 22.0, 19.6, 18.5, 13.6. IR(KBr): ν 3025, 2924, 1616, 1195, 819 cm^-1^. ESI-MS: m/z = 232 [M+H]^+^.

**Experimental procedures for the syntheses of terminal aminoalkyne substrate (1j).**

To a solution of EDC·HCl (2.30 g, 12.0 mmol) and HOBt (1.50 g, 11.2 mmol) in DCM (20.0 mL) was added a solution of 4-pentynoic acid (785 mg, 8.00 mmol) in DCM (10.0 mL), followed by addition of a solution of amines (12.0 mmol) in DCM (10.0 mL). The mixture was cooled to 0 ^o^C and Et_3_N (1.20 mL, 12.0 mmol) was added in dropwise. After being slowly warmed to room temperature and stirred overnight, the reaction mixture was diluted with DCM, washed successively with water, 5% hydrochloric acid, saturated NaHCO_3_ and brine, and dried over Na_2_SO_4_. Evaporation of the solvent followed by column chromatography on silica gel with EtOAc/Hexane (1:2) as eluent afforded 4-pentynoylamide with a yield of 91%.

To a solution of 4-pentynoylamide (4.00 mmol) in THF (30.0 mL) was slowly added lithium aluminum hydride (642 mg, 16.0 mmol) at 0 ^o^C. The reaction mixture was slowly warmed to room temperature and stirred for 24-30 h, and then quenched with Baechströms reagent (Na_2_SO_4_·10H_2_O) and stirred for 30 min. After filtration, the filtrate was evaporated and chromatographed on silica gel column with EtOAc/Hx (1:20) as eluent to afford aminoalkynes **1j** with a yield of 88%.

**General procedure for the synthesis of diversely substituted tetrahydropyrrolo[1,2-a]quinolines under microwave-assisted conditions:**

To a 5 mL Biotage Microwave vial equipped with a magnetic stir bar, CuCl (0.0100 mmol), aminoalkyne **1** (0.100 mmol), alkyne **2** (0.300 mmol) and DMF (3.00 mL) were added. The resulting mixture in sealed vial was stirred at 150 ^o^C under microwave irradiation for 15 min, and water (10.0 mL) was added to the vial to stop the reaction. The mixture wasthen extracted with AcOEt (3×10.0 mL), and the combined organic layers was washed with small amounts of water (5×5.00 mL) and dried with Na_2_SO_4_. The solvent was evaporated in vacuo and the residue was purified by silica gel column chromatography using n-hexane/EA as eluent to give the desired products.

Yellow oil; ^1^H NMR (500 MHz, CDCl_3_) δ 7.42 – 7.28 (m, 5H), 6.75 (dd, *J* = 8.5, 3.0 Hz, 1H), 6.59 (d, *J* = 3.0 Hz, 1H), 6.46 (d, *J* = 8.5 Hz, 1H), 5.71 (s, 1H), 3.65 (s, 3H), 3.47 – 3.36 (m, 2H), 2.13 – 2.02 (m, 3H), 1.97 – 1.91 (m, 1H), 1.10 (s, 3H). ^13^C NMR (125 MHz, CDCl_3_) δ 150.8, 140.0, 138.3, 135.8, 129.3, 129.2, 128.1, 127.2, 123.3, 114.2, 112.9, 112.8, 60.9, 55.9, 46.0, 38.1, 22.8, 20.7. IR(KBr): ν 3024, 2957, 2861, 1601, 1564, 1492, 1221, 803, 705 cm^-1^. ESI-MS: m/z = 292 [M+H]^+^.

Yellow oil; ^1^H NMR (500 MHz, CDCl_3_) δ 7.45 – 7.35 (m, 5H), 6.98 (d, *J* = 8.0 Hz, 1H), 6.78 (s, 1H), 6.45 (d, *J* = 8.0 Hz, 1H), 5.67 (s, 1H), 3.53 – 3.40 (m, 2H), 2.18 (s, 3H), 2.14 – 2.04 (m, 3H), 2.03 – 1.93 (m, 1H), 1.15 (s, 3H). ^13^C NMR (125 MHz, CDCl_3_) δ 141.4, 140.4, 135.9, 129.3, 129.2, 128.2, 128.1, 127.1, 126.8, 124.5, 122.1, 111.9, 60.9, 45.6, 38.2, 23.1, 20.6, 20.6. IR(KBr): ν 3025, 2956, 2849, 1601, 1559, 1487, 804, 706 cm^-1^. ESI-MS: m/z = 276 [M+H]^+^.

Yellow oil; ^1^H NMR (500 MHz, CDCl_3_) δ 7.44 – 7.37 (m, 3H), 7.35 – 7.31 (m, 2H), 7.07 (dd, *J* = 8.5, 2.5 Hz, 1H), 6.90 (d, *J* = 2.5 Hz, 1H), 6.41 (d, *J* = 8.5 Hz, 1H), 5.69 (s, 1H), 3.53 – 3.46 (m, 1H), 3.46 – 3.39 (m, 1H), 2.15 – 2.05 (m, 3H), 2.00-1.96 (m, 1H), 1.16 (s, 3H). ^13^C NMR (125 MHz, CDCl_3_) δ 141.9, 139.4, 135.2, 129.1, 128.8, 128.4, 128.3, 127.5, 125.8, 123.4, 120.3, 112.8, 61.2, 45.5, 38.1, 23.6, 20.5. IR (KBr): ν 3021, 2962, 2853, 1594, 1485, 1417, 804, 706 cm^-1^. ESI-MS: m/z = 296 [M+H]^+^.

Yellow oil;^1^H NMR (500 MHz, CDCl_3_) δ 7.47 – 7.38 (m, 3H), 7.38 – 7.33 (m, 2H), 7.22 (dd, *J* = 8.5, 2.5 Hz, 1H), 7.05 (d, *J* = 2.5 Hz, 1H), 6.38 (d, *J* = 8.5 Hz, 1H), 5.69 (s, 1H), 3.52-3.47 (m, 1H), 3.46 – 3.38 (m, 1H), 2.17 – 2.05 (m, 3H), 2.04 – 1.95 (m, 1H), 1.17 (s, 3H). ^13^C NMR (125 MHz, CDCl_3_) δ 142.2, 139.4, 135.1, 131.3, 129.1, 128.7, 128.6, 128.3, 127.5, 125.9, 124.0, 113.5, 61.3, 45.7, 38.1, 23.6, 20.5. IR(KBr): ν 3025, 2959, 2847, 1605, 1566, 1497, 808, 709 cm^-1^. ESI-MS: m/z = 340 [M+H]+.

Yellow oil; ^1^H NMR (500 MHz, CDCl_3_) δ 7.50 – 7.33 (m, 5H), 7.16 (t, *J* = 7.5 Hz, 1H), 6.97 (d, *J* = 7.5 Hz, 1H), 6.57 (t, *J* = 7.5 Hz, 1H), 6.52 (d, *J* = 7.5 Hz, 1H), 5.67 (s, 1H), 3.59 – 3.42 (m, 2H), 2.20 – 2.05 (m, 3H), 2.02-1.92 (m, 1H), 1.18 (s, 3H). ^13^C NMR (125 MHz, CDCl_3_) δ 143.4, 140.2, 136.0, 129.3, 128.9, 128.1, 127.8, 127.2, 126.3, 122.0, 115.4, 111.7, 61.1, 45.4, 38.2, 23.5, 20.5. IR (KBr): ν 3031, 2956, 2837, 1602, 1507, 710 cm^-1^. ESI-MS: m/z = 262 [M+H]^+^.

Yellow oil; ^1^H NMR (500 MHz, CDCl_3_) δ 7.42 – 7.30 (m, 5H), 6.83 (d, *J* = 7.5 Hz, 1H), 6.37 (d, *J* = 7.5 Hz, 1H), 6.32 (s, 1H), 5.59 (s, 1H), 3.53 – 3.41 (m, 2H), 2.31 (s, 3H), 2.14 – 2.01 (m, 3H), 1.98-1.94 (m, 1H), 1.14 (s, 3H).^13^C NMR (125 MHz, CDCl_3_) δ 143.4, 140.4, 138.9, 135.8, 129.2, 128.1, 127.1, 126.8, 126.2, 119.5, 116.2, 112.4, 61.1, 45.3, 38.2, 23.5, 21.8, 20.6. IR (KBr): ν 3028, 2960, 2917, 2879, 1603, 1490, 1456, 714 cm^-1^. ESI-MS: m/z = 276 [M+H]^+^.

Yellow oil; ^1^H NMR (500 MHz, CDCl_3_) δ 7.44 – 7.33 (m, 5H), 6.76 (d, *J* = 8.5 Hz, 1H), 6.60 (s, 1H), 6.49 (d, *J* = 8.5 Hz, 1H), 5.61 (s, 1H), 3.67 (s, 3H), 3.55 (s, 1H), 3.43 (s, 1H), 2.09 – 1.96 (m, 4H), 1.72-1.64 (m, 1H), 1.42 – 1.33 (m, 1H), 0.94 (t, *J* = 7.5 Hz, 3H). ^13^C NMR (125 MHz, CDCl_3_) δ 150.4, 140.2, 139.2, 136.6, 129.2, 128.2, 127.9, 127.3, 123.06, 114.2, 112.8, 112.7, 64.2, 55.9, 47.8, 36.7, 31.5, 20.9, 8.4. IR (KBr): ν 3021, 2956, 2840, 1602, 1567, 1506, 1221, 808 cm^-1^. ESI-MS: m/z = 306 [M+H]^+^.

Yellow oil; ^1^H NMR (500 MHz, CDCl_3_) δ 7.45 – 7.31 (m, 5H), 6.75 (dd, *J* = 8.5, 2.5 Hz, 1H), 6.58 (d, *J* = 2.5 Hz, 1H), 6.47 (d, *J* = 8.5 Hz, 1H), 5.61 (s, 1H), 3.66 (s, 3H), 3.53 (s, 1H), 3.41 (s, 1H), 2.09 – 1.94 (m, 4H), 1.67-1.59 (m, 1H), 1.46-1.36 (m, 2H), 1.34-1.26 (m, 1H), 0.89 (t, *J* = 7.0 Hz, 3H). ^13^C NMR (125 MHz, CDCl_3_) δ 150.5, 140.2, 139.2, 136.2, 129.2, 128.2, 127.8, 127.2, 123.1, 114.2, 112.8, 112.7, 63.9, 55.9, 47.7, 41.5, 37.2, 20.8, 17.3, 14.8. IR(KBr): ν cm^-1^. IR(KBr): ν 3021, 2920, 2853, 1604, 1539, 1254, 805 cm^-1^. ESI-MS: m/z = 320 [M+H]^+^.

Yellow oil; ^1^H NMR (500 MHz, CDCl_3_) δ 7.44 – 7.31 (m, 5H), 6.75 (d, *J* = 8.5 Hz, 1H), 6.59 (s, 1H), 6.47 (d, *J* = 8.5 Hz, 1H), 5.61 (s, 1H), 3.66 (s, 3H), 3.60 – 3.32 (m, 2H), 2.10 – 1.94 (m, 4H), 1.68-1.56(m, 1H), 1.45 – 1.36 (m, 1H), 1.35 – 1.24 (m, 4H), 0.88 (t, *J* = 7.0 Hz, 3H). ^13^C NMR (125 MHz, CDCl_3_) δ 150.5, 140.2, 139.1, 136.2, 129.2, 128.2, 127.8, 127.2, 123.1, 114.2, 112.9, 112.7, 63.8, 55.9, 47.7, 38.5, 37.0, 26.1, 23.3, 20.9, 14.1. IR (KBr): ν 3021, 2956, 2849, 1602, 1481, 807 cm^-1^. ESI-MS: m/z = 334 [M+H]^+^.

Yellow solid; ^1^H NMR (500 MHz, CDCl_3_) δ 7.30 (d, *J* = 8.5 Hz, 2H), 6.94 (d, *J* = 8.5 Hz, 2H), 6.77 (dd, *J* = 8.5, 3.0 Hz, 1H), 6.64 (s, 1H), 6.48 (s, 1H), 5.71 (s, 1H), 3.87 (s, 3H), 3.69 (s, 3H), 3.44 (s, 2H), 2.16 – 2.03 (m, 3H), 2.02 – 1.90 (m, 1H), 1.11 (s, 3H). ^13^C NMR (125 MHz, CDCl_3_) δ 159.0, 150.8, 138.3, 135.2, 132.4, 130.2, 128.9, 123.6, 114.2, 113.6, 112.9, 112.8, 60.8, 55.9, 55.3, 45.8, 38.1, 22.6, 20.8. IR (KBr): ν 2959, 2919, 2851, 1507, 1496, 1488, 1244, 807 cm^-1^. ESI-MS: m/z = 322 [M+H]^+^.

Yellow oil; ^1^H NMR (500 MHz, CDCl_3_) δ 7.28 (d, *J* = 8.5 Hz, 2H), 7.21 (d, *J* = 7.5 Hz, 2H), 6.81 – 6.72 (m, 1H), 6.65 (s, 1H), 6.48 (d, *J* = 7.5 Hz, 1H), 5.72 (s, 1H), 3.69 (s, 3H), 3.52-3.36 (m, 2H), 2.42 (s, 3H), 2.16 – 2.02 (m, 3H), 2.02-1.92 (m, 1H), 1.13 (s, 3H). ^13^C NMR (125 MHz, CDCl_3_) δ 150.74, 138.30, 137.03, 136.91, 135.61, 129.04, 128.86, 123.46, 114.15, 112.91, 112.76, 60.88, 55.94, 45.84, 38.06, 22.58, 21.20, 20.74. IR (KBr): ν 3021, 2956, 2864, 2830, 1605, 1564, 1492, 1366, 1292, 1220, 1045, 810 cm^-1^. ESI-MS: m/z = 306 [M+H]^+^.

Yellow oil; ^1^H NMR (500 MHz, CDCl_3_) δ 7.34 (d, *J* = 8.5 Hz, 2H), 7.28 (d, *J* = 8.5 Hz, 2H), 6.76 (dd, *J* = 8.5, 3.0 Hz, 1H), 6.52 (d, *J* = 3.0 Hz, 1H), 6.46 (d, *J* = 8.5 Hz, 1H), 5.68 (s, 1H), 3.66 (s, 3H), 3.43-3.38 (m, 2H), 2.12 – 2.01 (m, 3H), 1.99-1.91 (m, 1H), 1.09 (s, 3H). ^13^C NMR (125 MHz, CDCl_3_) δ 150.8, 138.5, 138.2, 134.9, 133.2, 130.5, 129.6, 128.4, 122.9, 114.5, 113.0, 112.7, 60.9, 55.9, 45.8, 38.0, 22.6, 20.8. IR (KBr): ν 3039, 2970, 2923, 2893, 2843, 1607, 1563, 1461, 1226, 806 cm^-1^. ESI-MS: m/z = 326 [M+H]^+^.

Yellow oil; ^1^H NMR (600 MHz, CDCl_3_) δ 6.87 – 6.80 (m, 1H), 6.76 – 6.70 (m, 1H), 6.41-6.38 (m, 1H), 5.59 (s, 1H), 3.80 (s, 3H), 3.45-3.37 (m, 1H), 3.36-3.29 (m, 1H), 2.45 – 2.30 (m, 2H), 2.05-1.96 (m, 3H), 1.92-1.87 (m, 1H), 1.60 – 1.50 (m, 2H), 1.48 – 1.38 (m, 2H), 1.03 (s, 3H), 0.99-0.93 (m, 3H). ^13^C NMR (150 MHz, CDCl_3_) δ 150.8, 138.3, 132.3, 127.2, 123.4, 113.1, 112.4, 110.7, 60.7, 56.0, 46.1, 38.3, 31.9, 30.79, 23.3, 22.7, 20.6, 14.1. IR(KBr): ν 2959, 2928, 2862, 1603, 1492, 1258, 1040, 807 cm^-1^. ESI-MS: m/z = 272 [M+H]^+^.

Yellow oil; ^1^H NMR (600 MHz, CDCl_3_) δ 7.35-7.23 (m, 5H), 6.78 (s, 1H), 6.73 (s, 1H), 6.41 (s, 1H), 5.50 (s, 1H), 3.87 – 3.73 (m, 2H), 3.70 (s, 3H), 3.45 (s, 1H), 3.36 (s, 1H), 2.03 (s, 3H), 1.91 (s, 1H), 1.10 (s, 3H). ^13^C NMR (150 MHz, CDCl_3_) δ 150.8, 139.8, 138.2, 131.2, 129.7, 128.8, 128.5, 126.1, 123.1, 113.8, 112.5, 111.0, 61.0, 55.9, 46.3, 38.8, 38.3, 23.6, 20.7. IR (KBr): ν 3021, 2956, 2847, 1601, 1503, 805 cm^-1^. ESI-MS: m/z = 306 [M+H]^+^.

Yellow oil; ^1^H NMR (600 MHz, CDCl_3_) δ 7.34-7.28 (m, 2H), 7.24 – 7.18 (m, 3H), 6.86 (d, *J* = 2.4 Hz, 1H), 6.74 (dd, *J* = 9.0, 2.4 Hz, 1H), 6.40 (d, *J* = 9.0 Hz, 1H), 5.50 (s, 1H), 3.77 (s, 3H), 3.45-3.38 (m, 1H), 3.34-3.29 (m, 1H), 2.89-2.86 (m, 2H), 2.68-2.65 (m, 2H), 2.05-1.90 (m, 3H), 1.88-1.84 (m, 1H), 0.98 (s, 3H). ^13^C NMR (150 MHz, CDCl_3_) δ 150.9, 142.2, 138.3, 131.4, 128.6, 128.3, 127.7, 125.9, 123.1, 113.5, 112.6, 110.4, 60.6, 56.1, 46.3, 38.2, 34.9, 34.2, 23.4, 20.7. IR (KBr): ν 3025, 2956, 2856,1603, 1566, 1496, 1368, 803, 700 cm^-1^. ESI-MS: m/z = 320 [M+H]^+^.

Yellow solid; ^1^H NMR (500 MHz, CDCl_3_) δ 6.93 (d, *J* = 9.0 Hz, 2H), 6.88 – 6.80 (m, 3H), 6.75 (dd, *J* = 8.5, 2.5 Hz, 1H), 6.41 (s, 1H), 5.85 (s, 1H), 4.79 (d, *J* = 12.0 Hz, 1H), 4.73 (d, *J* = 12.0 Hz, 1H), 3.78 (s, 4H), 3.72 (s, 3H), 3.44 (s, 1H), 3.31 (s, 1H), 2.05 – 1.95 (m, 3H), 1.94 – 1.87 (m, 1H), 1.04 (s, 3H). ^13^C NMR (125 MHz, CDCl_3_) δ 154.0, 152.9, 150.8, 138.1, 129.6, 128.1, 121.3, 116.2, 114.59, 114.3, 112.7, 110.3, 69.5, 60.8, 56.0, 55.7, 46.4, 38.1, 23.6, 20.6. IR(KBr): ν 3025, 2956, 2857, 1499, 1225, 810 cm^-1^. ESI-MS: m/z = 335 [M+H]^+^.

Yellow solid; ^1^H NMR (500 MHz, CDCl_3_) δ 6.93 (d, *J* = 9.0 Hz, 2H), 6.84 (d, *J* = 9.0 Hz, 2H), 6.83 (s, 1H), 6.75 (dd, *J* = 8.5, 2.5 Hz, 1H), 6.42 (s, 1H), 5.86 (s, 1H), 4.79 (d, *J* = 12.0 Hz, 1H), 4.73 (d, *J* = 12.0 Hz, 1H), 3.78 (s, 3H), 3.73 (s, 3H), 3.44 (s, 1H), 3.32 (s, 1H), 2.04 – 1.94 (m, 3H), 1.94-1.86 (m, 1H), 1.04 (s, 3H). ^13^C NMR (125 MHz, CDCl_3_) δ 154.0, 152.9, 150.8, 138.1, 129.6, 128.1, 121.2, 116.2, 114.6, 114.3, 112.7, 110.3, 69.5, 60.8, 56.0, 55.7, 46.4, 38.1, 23.7, 20.6. IR(KBr): ν 3046, 2955, 2876, 2829, 1565, 1505, 1219, 805 cm^-1^. ESI-MS: m/z = 352 [M+H]^+^.

**Procedure for the isolation of compound C:**

To a 5 mL Biotage Microwave vial equipped with a magnetic stir bar, CuCl (0.0100 mmol), aminoalkyne **1** (0.100 mmol), alkyne **2** (0.300 mmol) and DMF (3.00 mL) were added. The resulting mixture in sealed vial was stirred at 150 ^o^C under for 2 h, and water (10.0 mL) was added to the vial to stop the reaction. The mixture wasthen extracted with AcOEt (3×10.0 mL), and the combined organic layers was washed with small amounts of water (5×5.00 mL) and dried with Na_2_SO_4_. The solvent was evaporated in vacuo and the residue was purified by silica gel column chromatography using n-hexane/EA as eluent to give the desired products.

Yellow oil; ^1^H NMR (500 MHz, CDCl_3_) δ 7.44 – 7.39 (m, 2H), 7.31 (dd, *J* = 6.5, 2.5 Hz, 3H), 7.11 (d, *J* = 8.5 Hz, 2H), 7.00 (d, *J* = 8.5 Hz, 2H), 3.8-3.52 (m, 7.0 Hz, 1H), 3.51 – 3.42 (m, 1H), 2.58-2.51 (m, 1H), 2.31 (s, 3H), 2.23 – 2.15 (m, 2H), 2.07 – 2.01 (m, 1H), 1.71 (s, 3H). HRMS (ESI) [M+H] calculated for [C_20_H_22_N]^+^ 276.1747, found 276.1748.

**Table S1**  Cytotoxic activities of compounds 3aa, 3ag, 3ha, 3ai, 3ab and 3ah against cancer cells (CFPAC1 and CAPAN2).

| Compd. | CFPAC1 | | CAPAN2 | |
| --- | --- | --- | --- | --- |
|  | Concentration (μM) | Inhibition rate (%) | Concentration (μM) | Inhibition rate (%) |
| 3aa | 0.4 | 4.61 | 0.4 | -0.06 |
|  | 20 | 4.45 | 20 | -1.20 |
|  | 100 | 8.22 | 100 | 35.3 |
| 3ag | 0.4 | 2.42 | 0.4 | -3.07 |
|  | 20 | 5.39 | 20 | 8.76 |
|  | 100 | 14.4 | 100 | 59.0 |
| 3ha | 0.4 | 7.32 | 0.4 | -4.54 |
|  | 20 | 5.47 | 20 | -0.279 |
|  | 100 | 27.8 | 100 | 68.26 |
| 3ai | 0.4 | 4.90 | 0.4 | -0.93 |
|  | 20 | 4.39 | 20 | 1.62 |
|  | 100 | 5.84 | 100 | 41.2 |
| 3ab | 0.4 | 2.00 | 0.4 | 0.158 |
|  | 20 | 4.09 | 20 | 0.119 |
|  | 100 | 4.31 | 100 | 12.9 |
| 3ah | 0.4 | 4.08 | 0.4 | 0.49 |
|  | 20 | 4.06 | 20 | 5.354 |
|  | 100 | 5.52 | 100 | 50.1 |


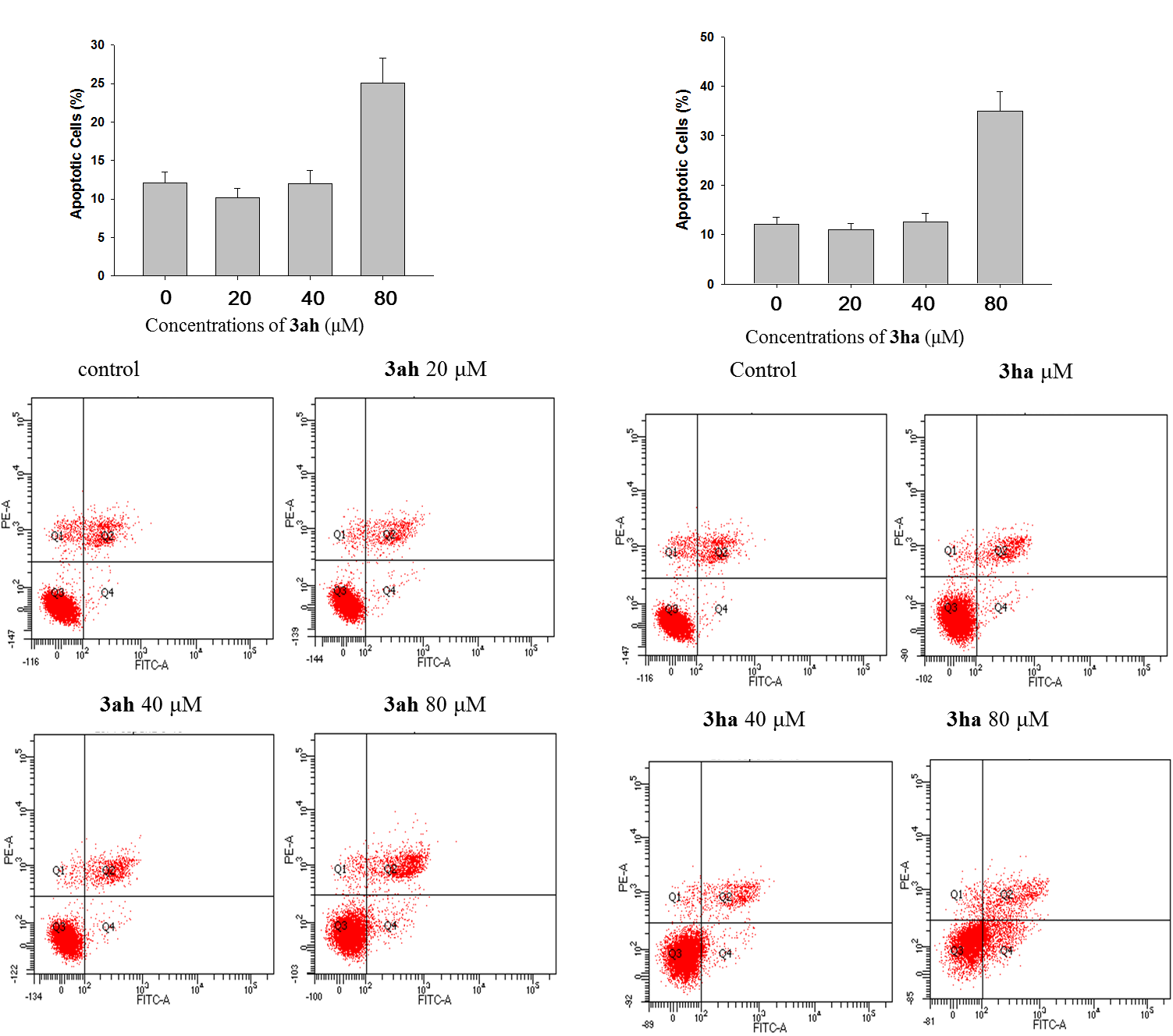


**Figure S1** **3ha** and **3ah** mitochondrion-dependent apoptosis in CAPAN2 cells. CAPAN2 cells were seeded in 6-well plate and cultured for 24 h. Cells were treated with **3ha or 3ah** for 48 h before staining and followed by flow cytometric analysis. Three independent experiments were quantitatively analyzed. Each bar represented the mean ± SD. *** P<0.001.


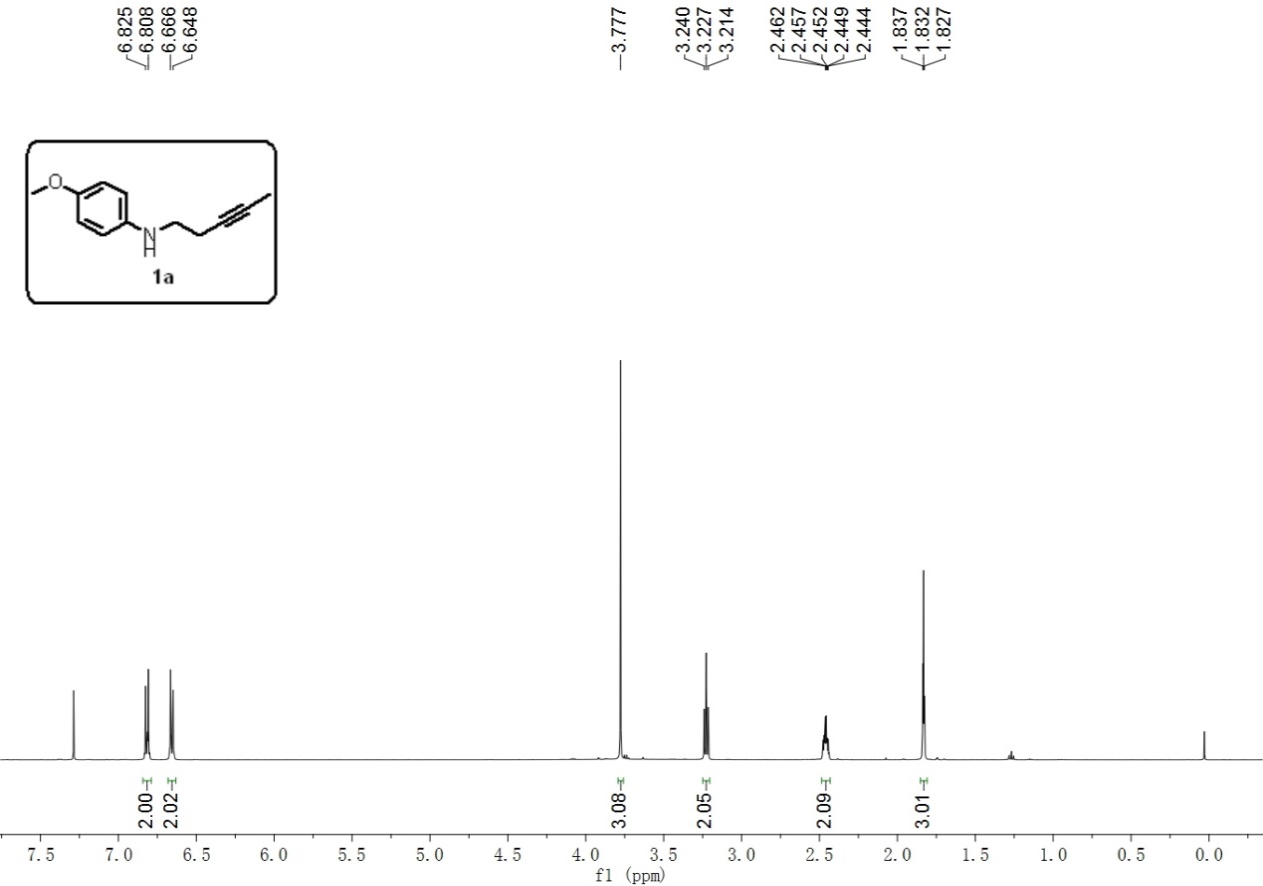


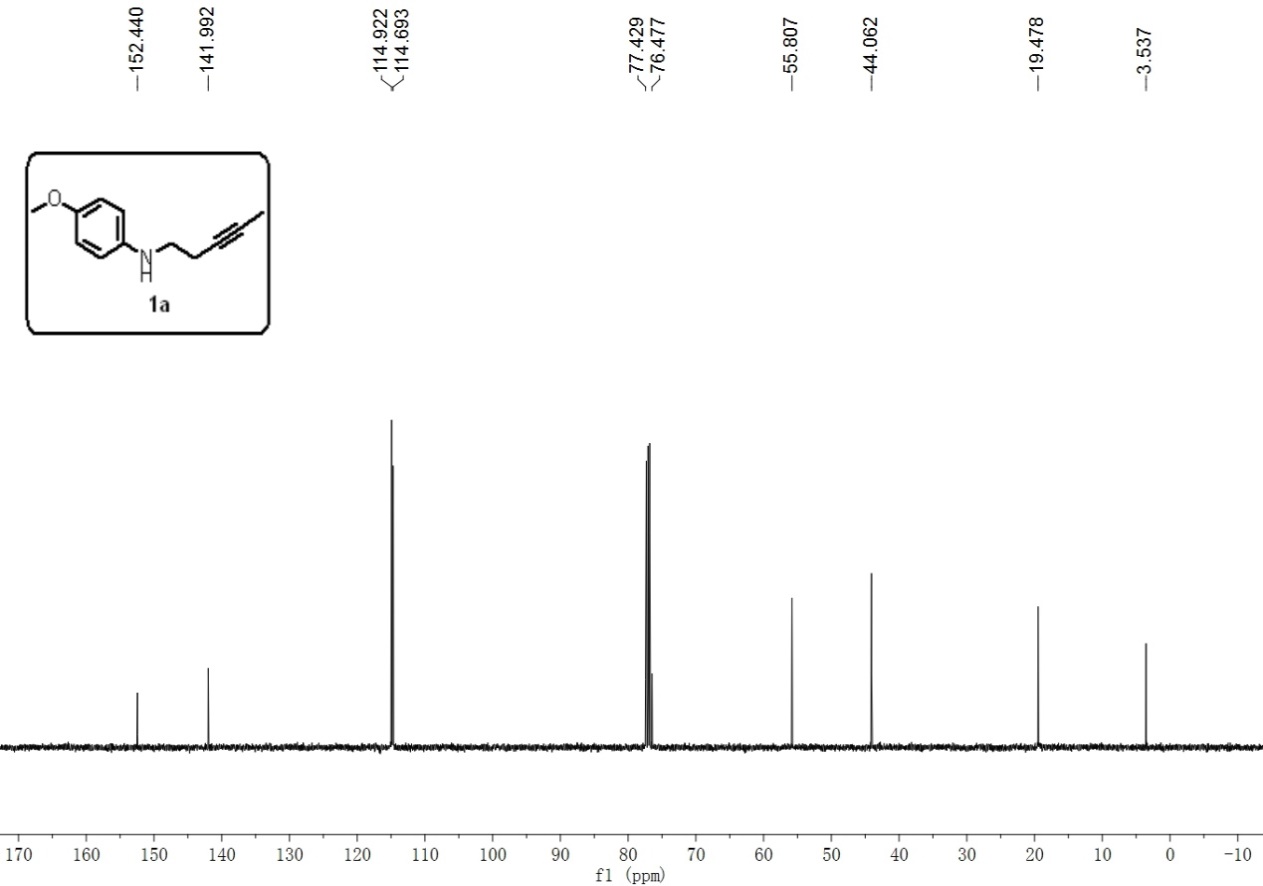


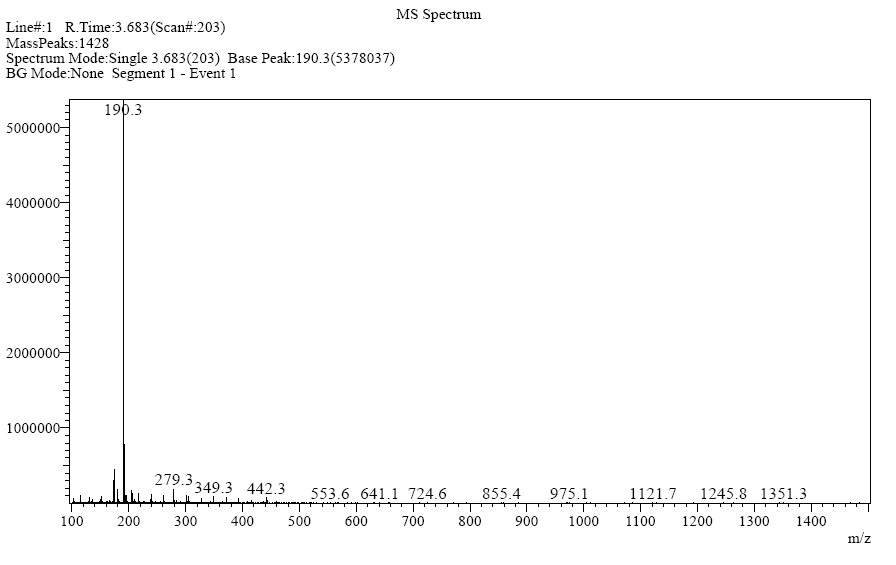


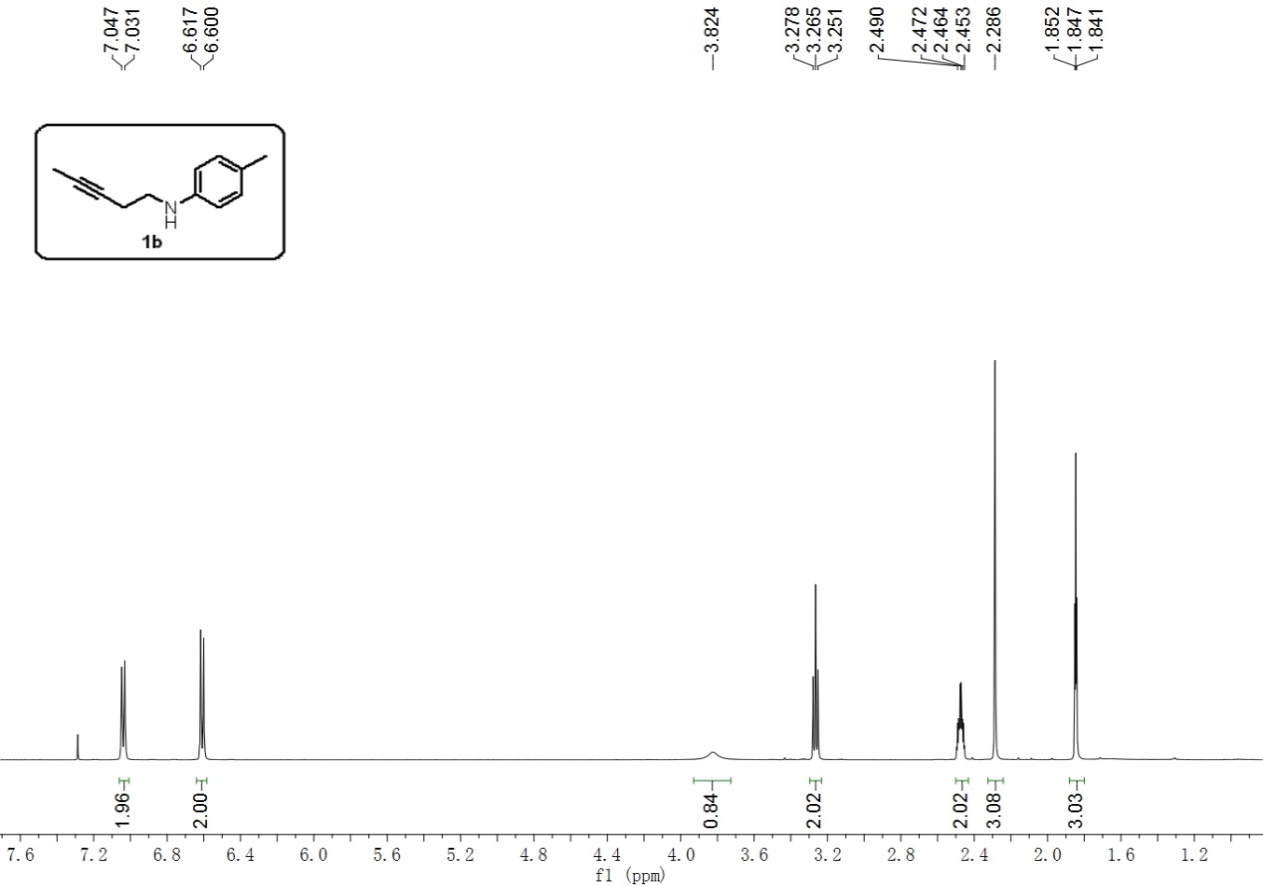


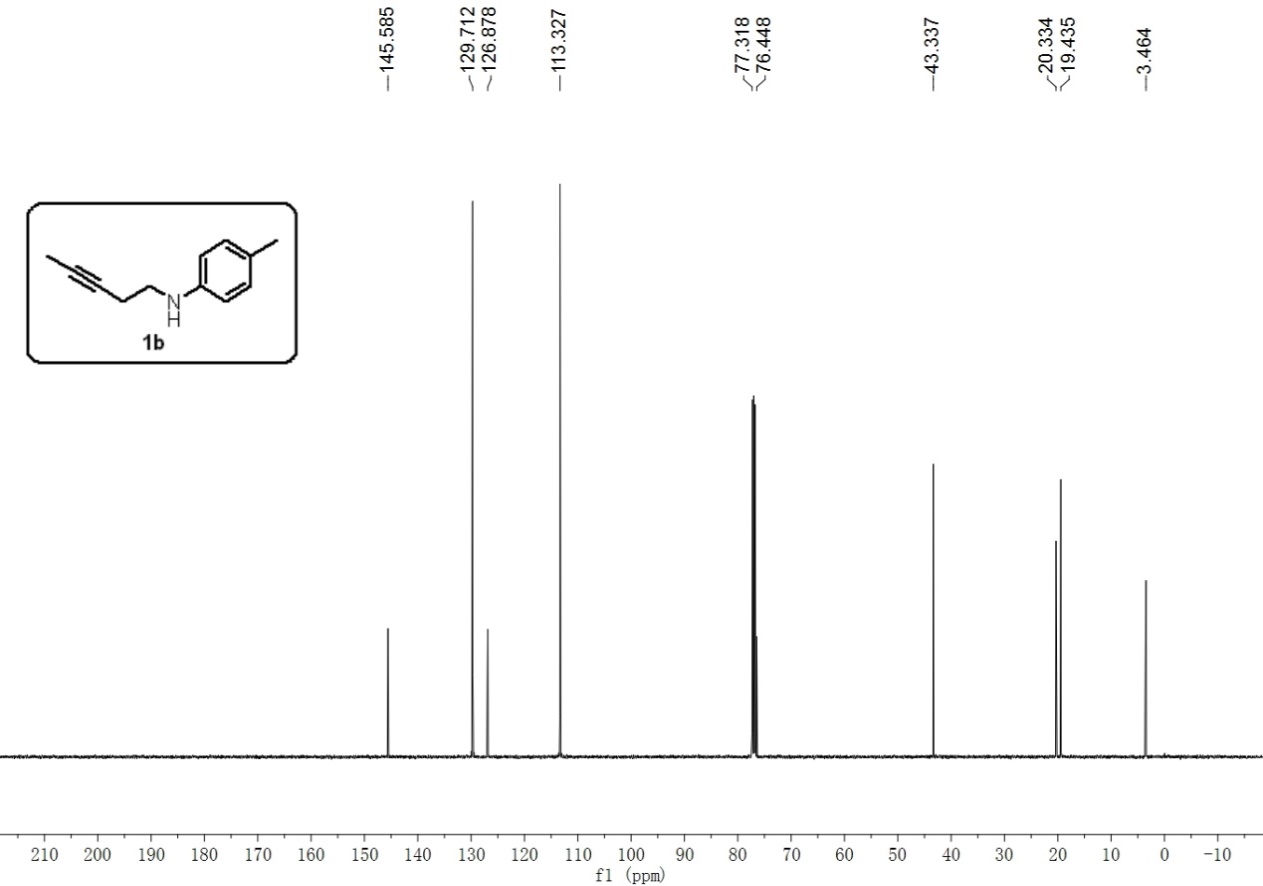


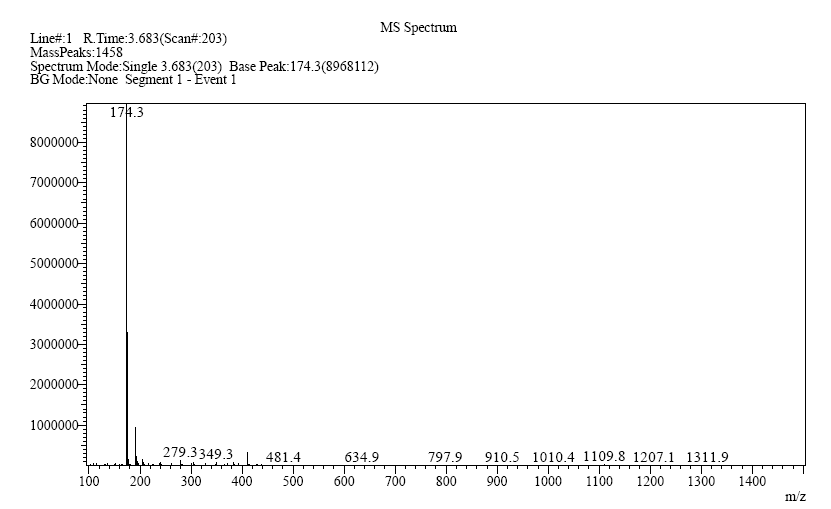


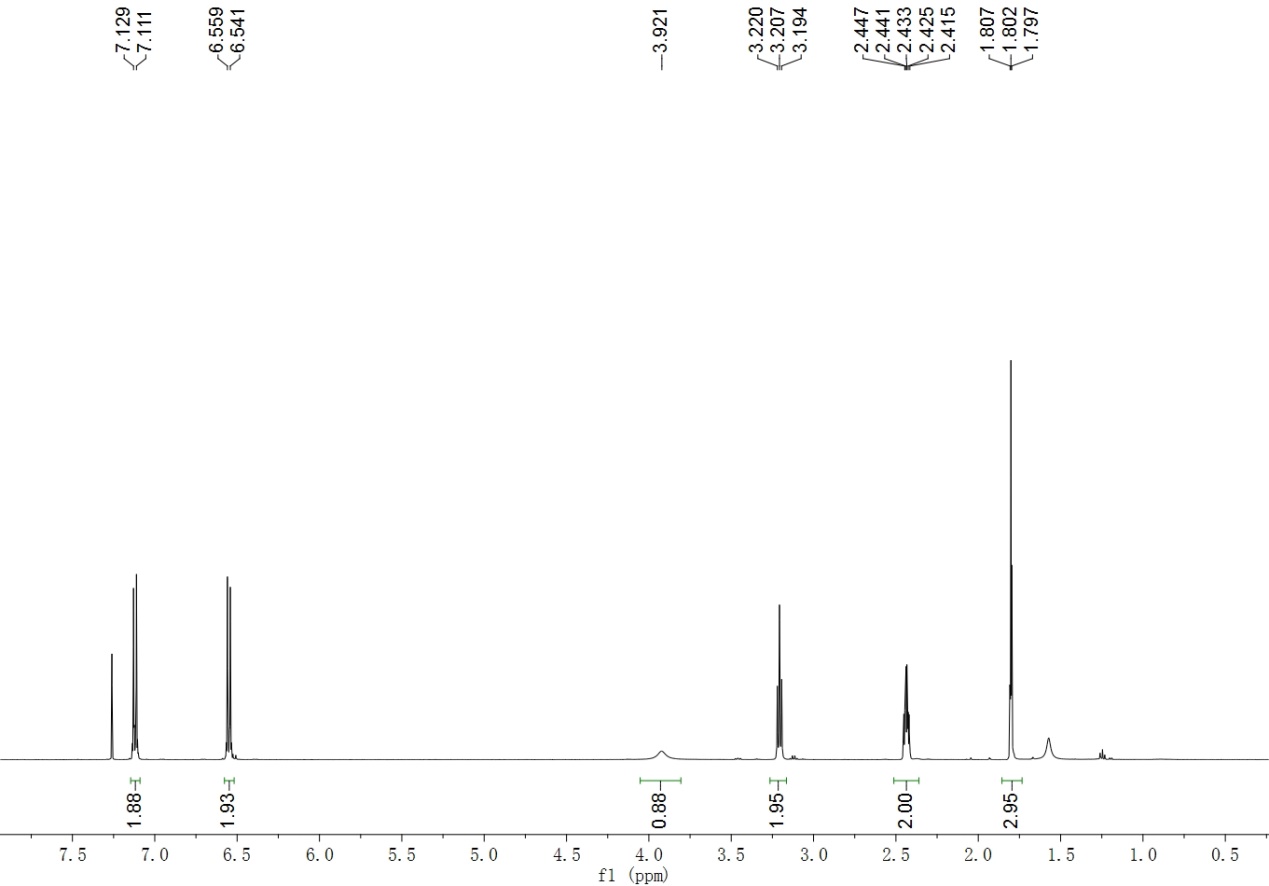


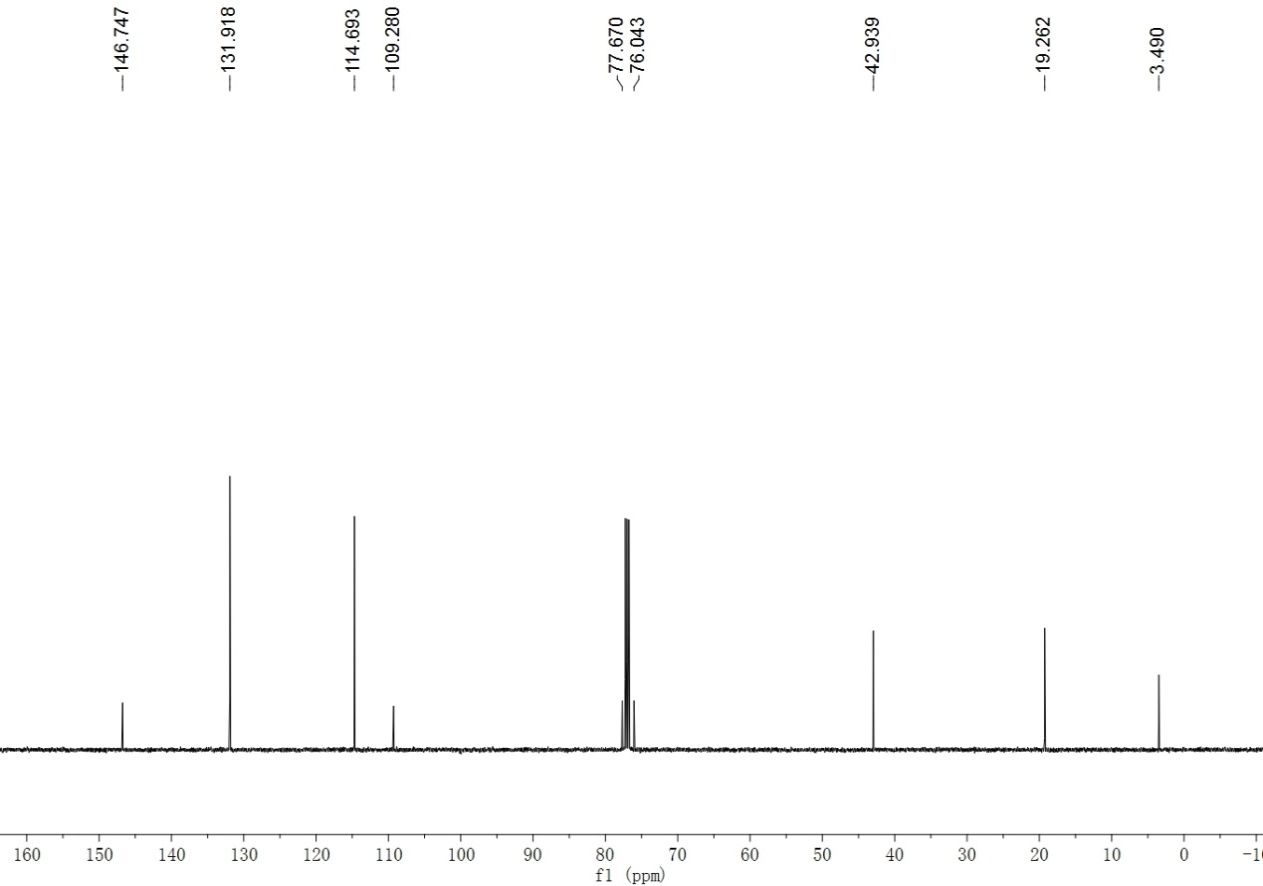


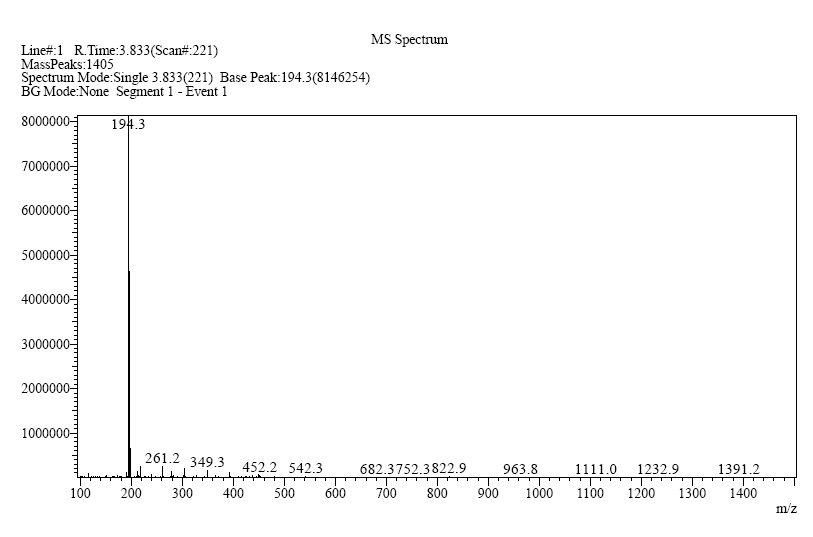


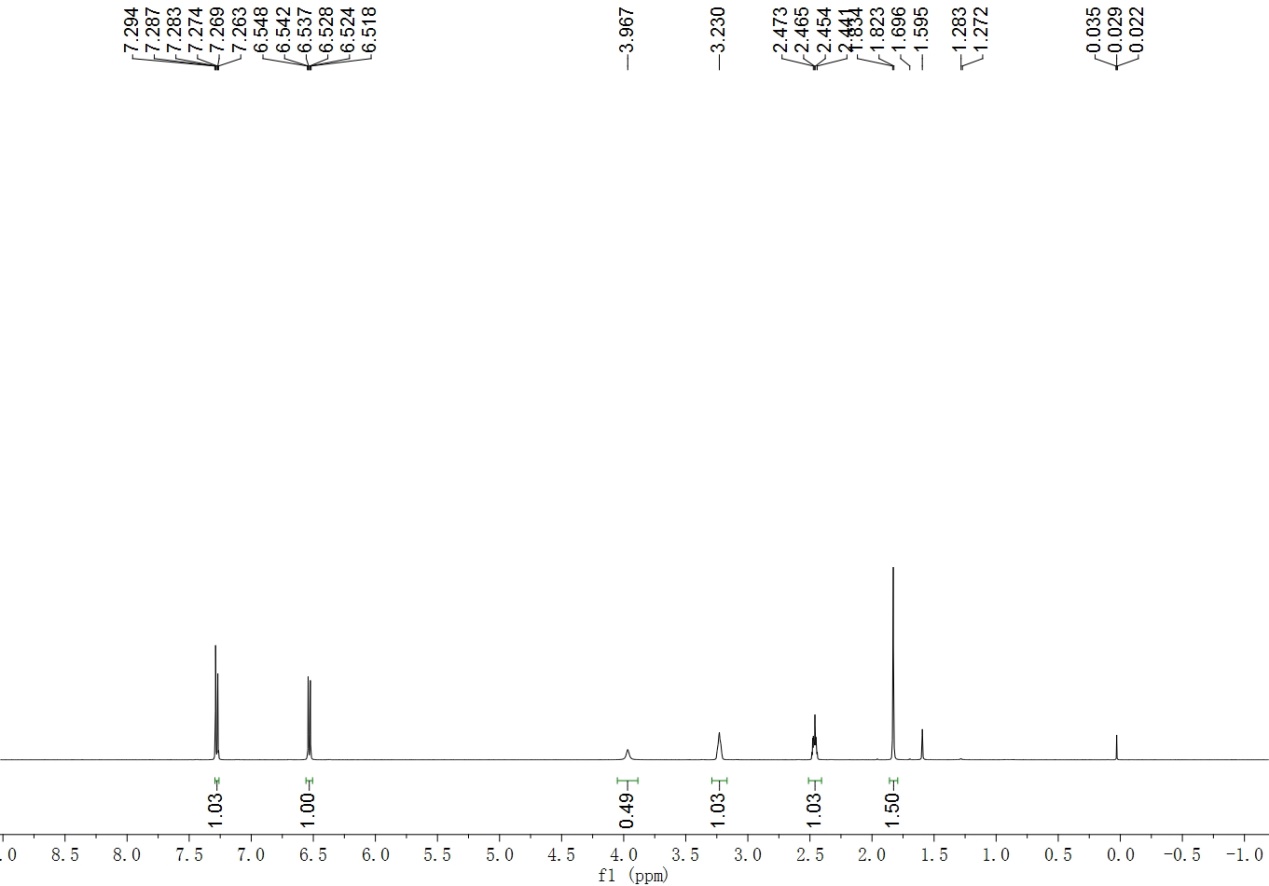


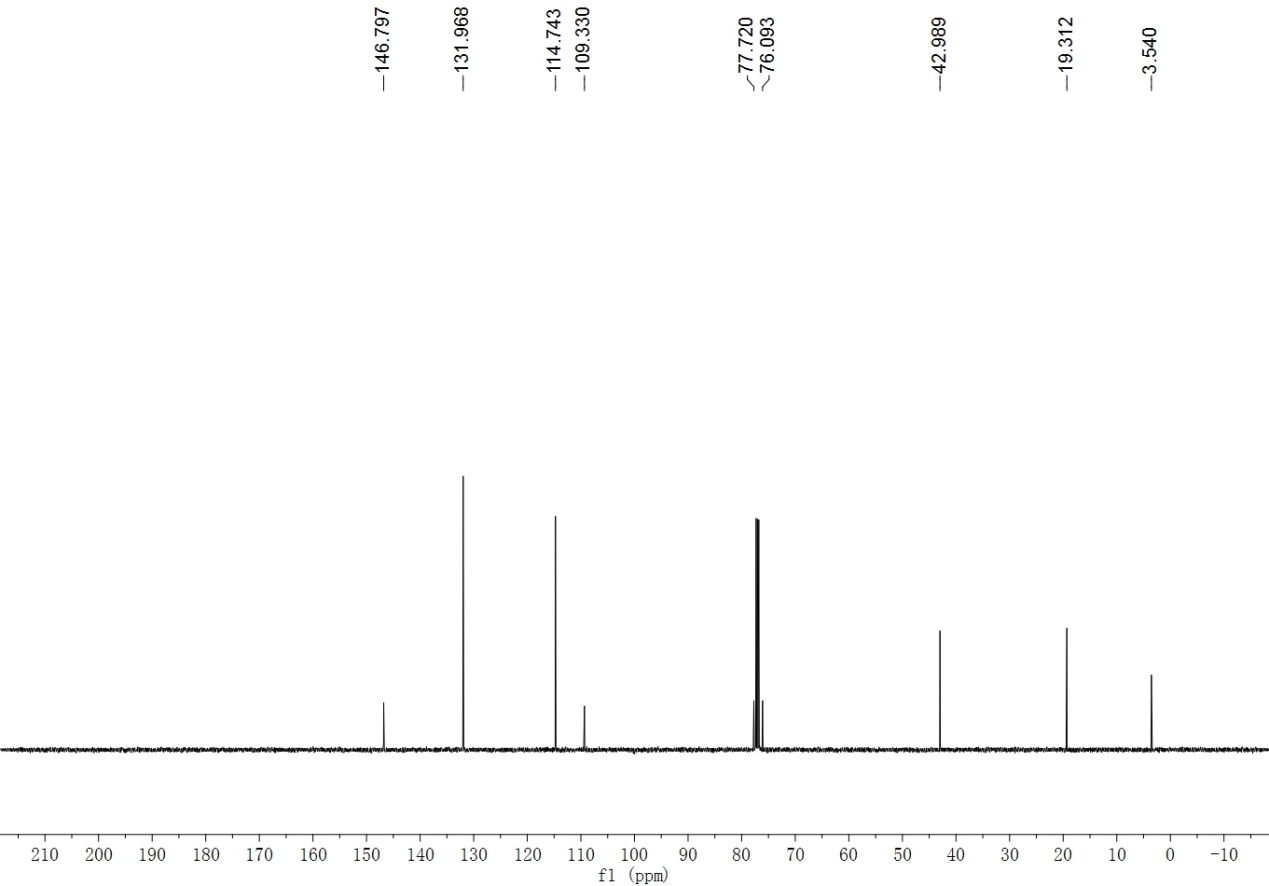


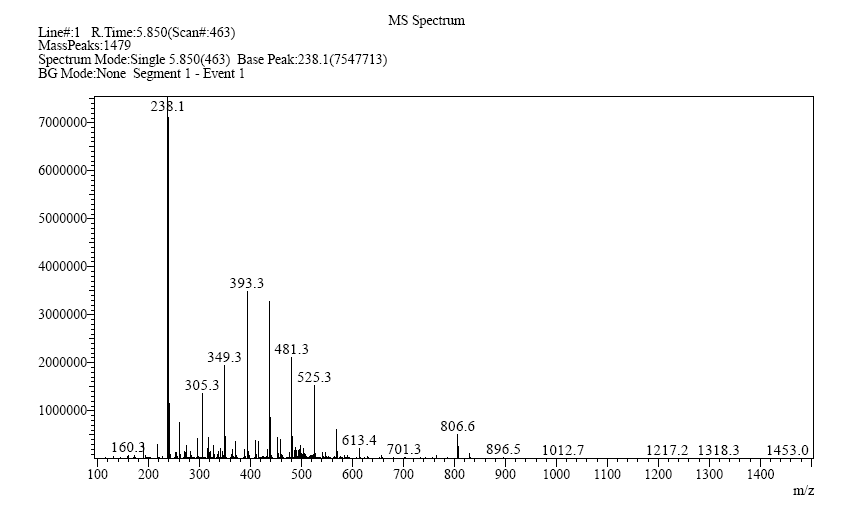


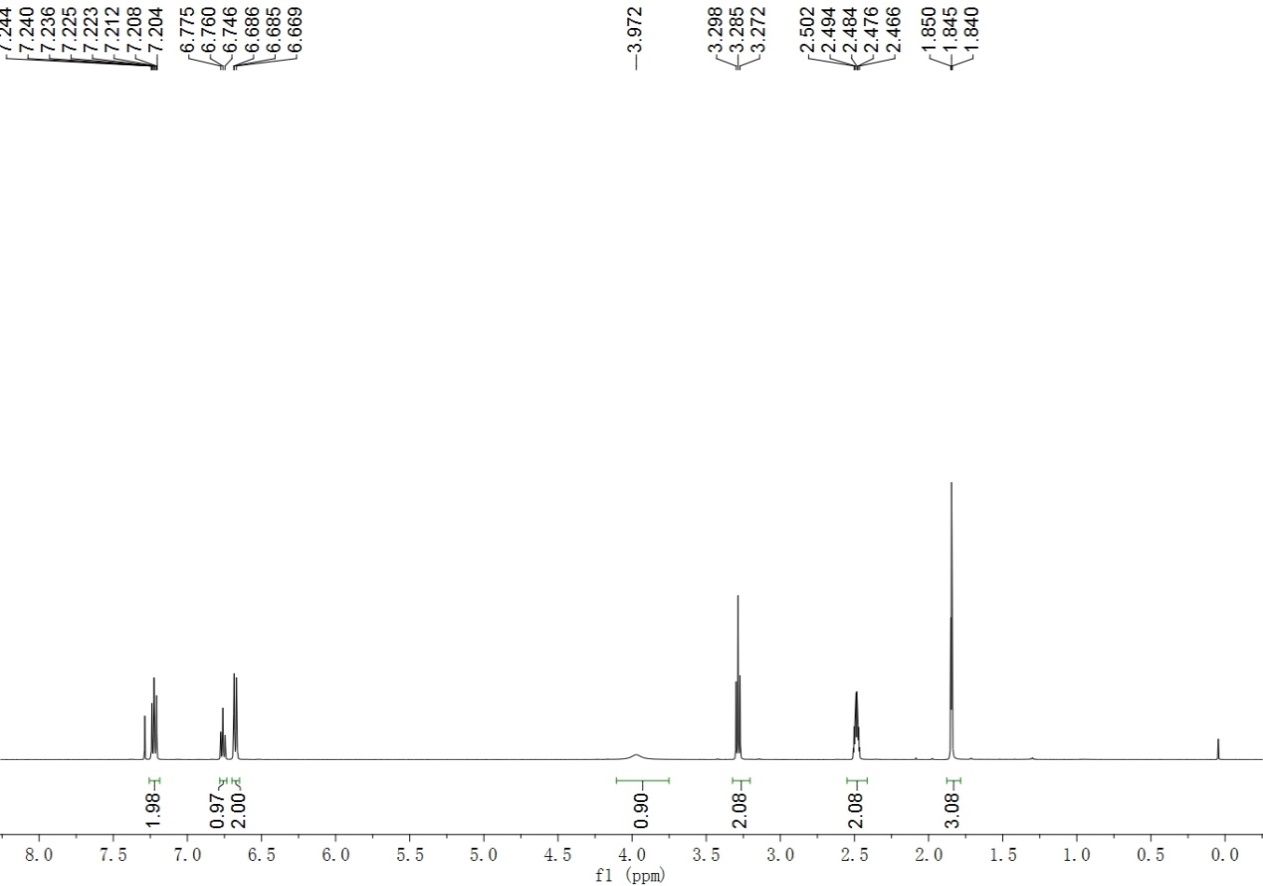


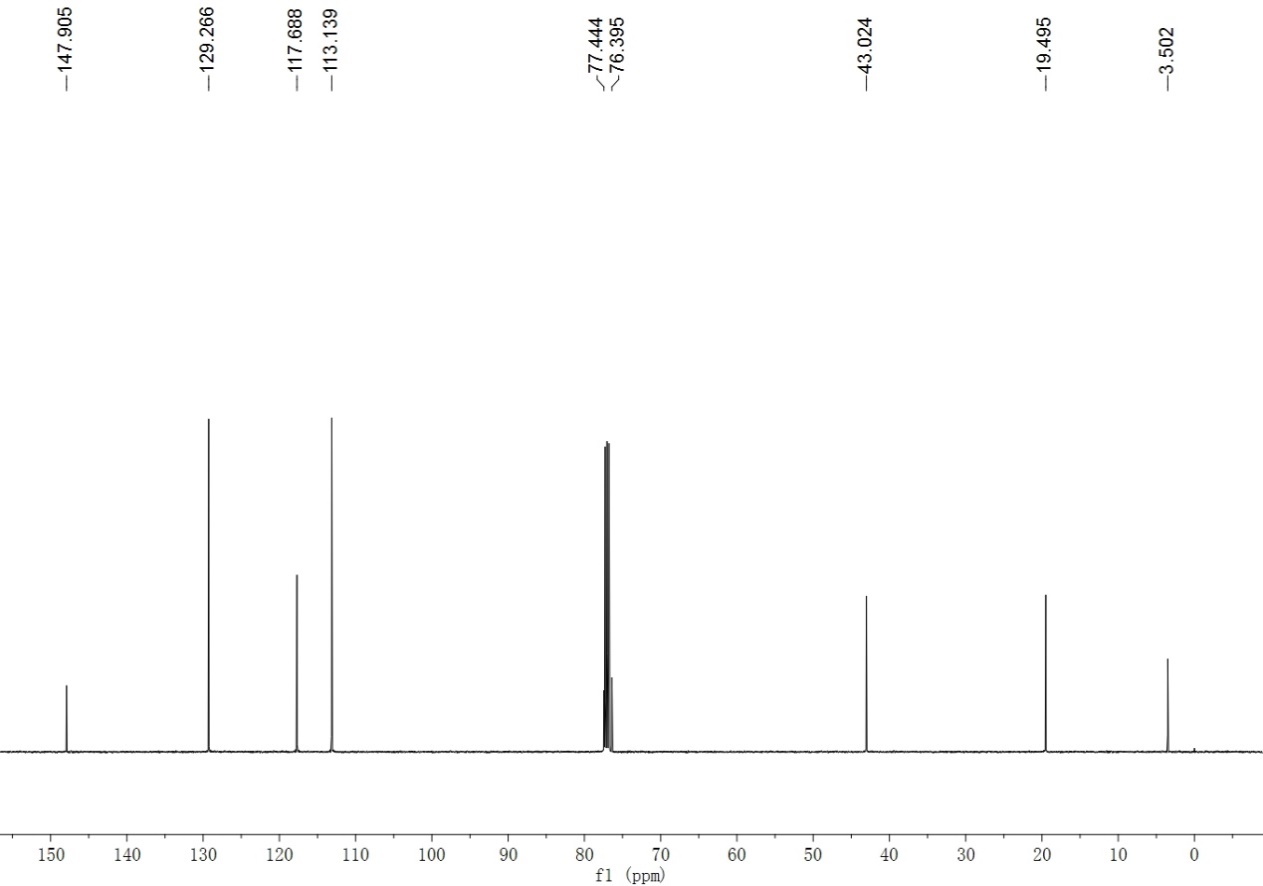


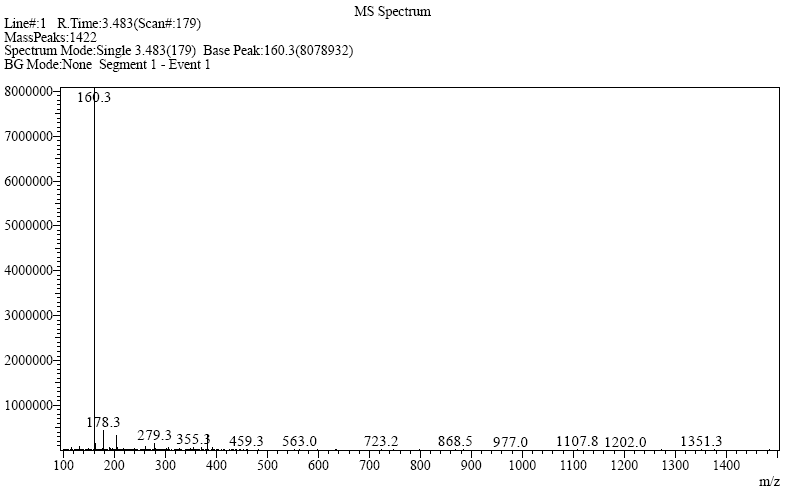


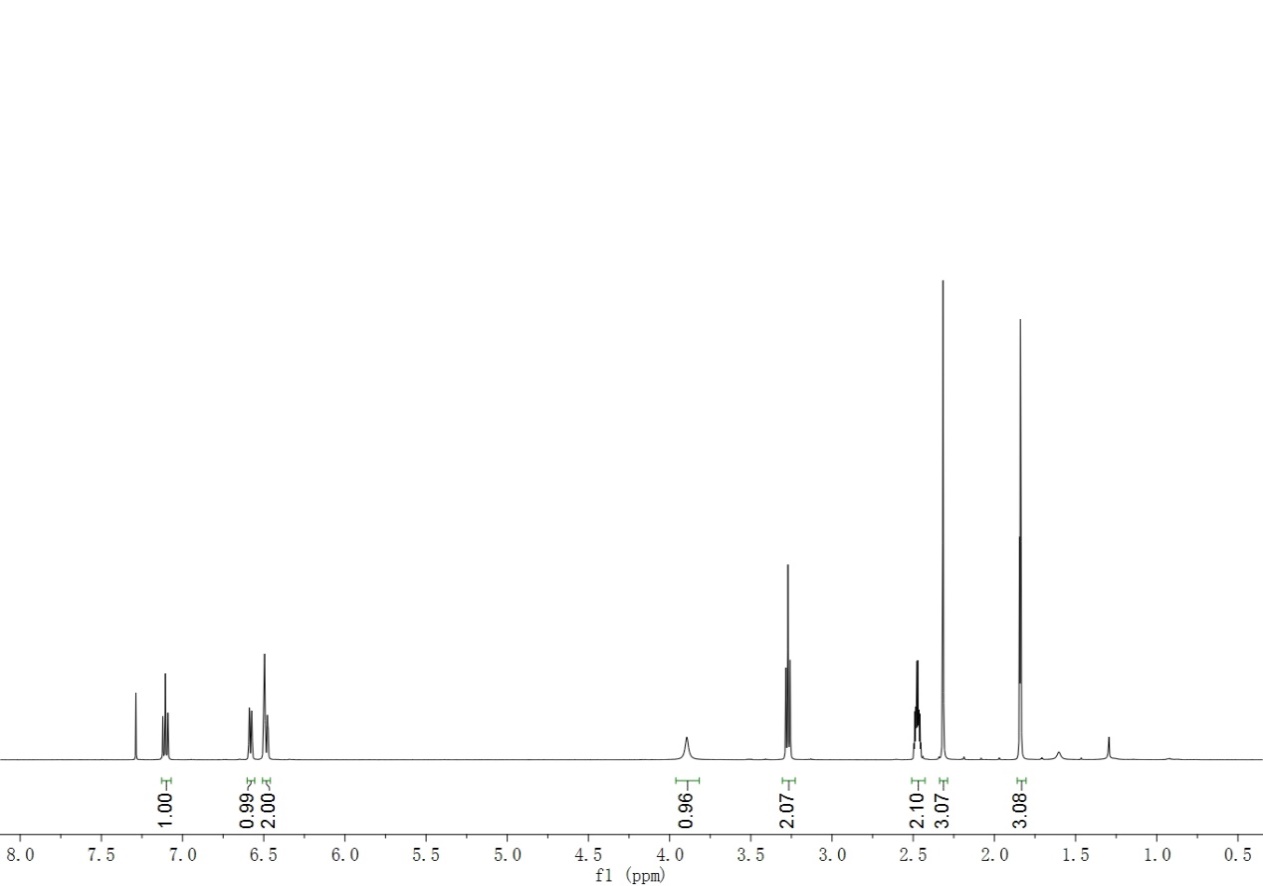


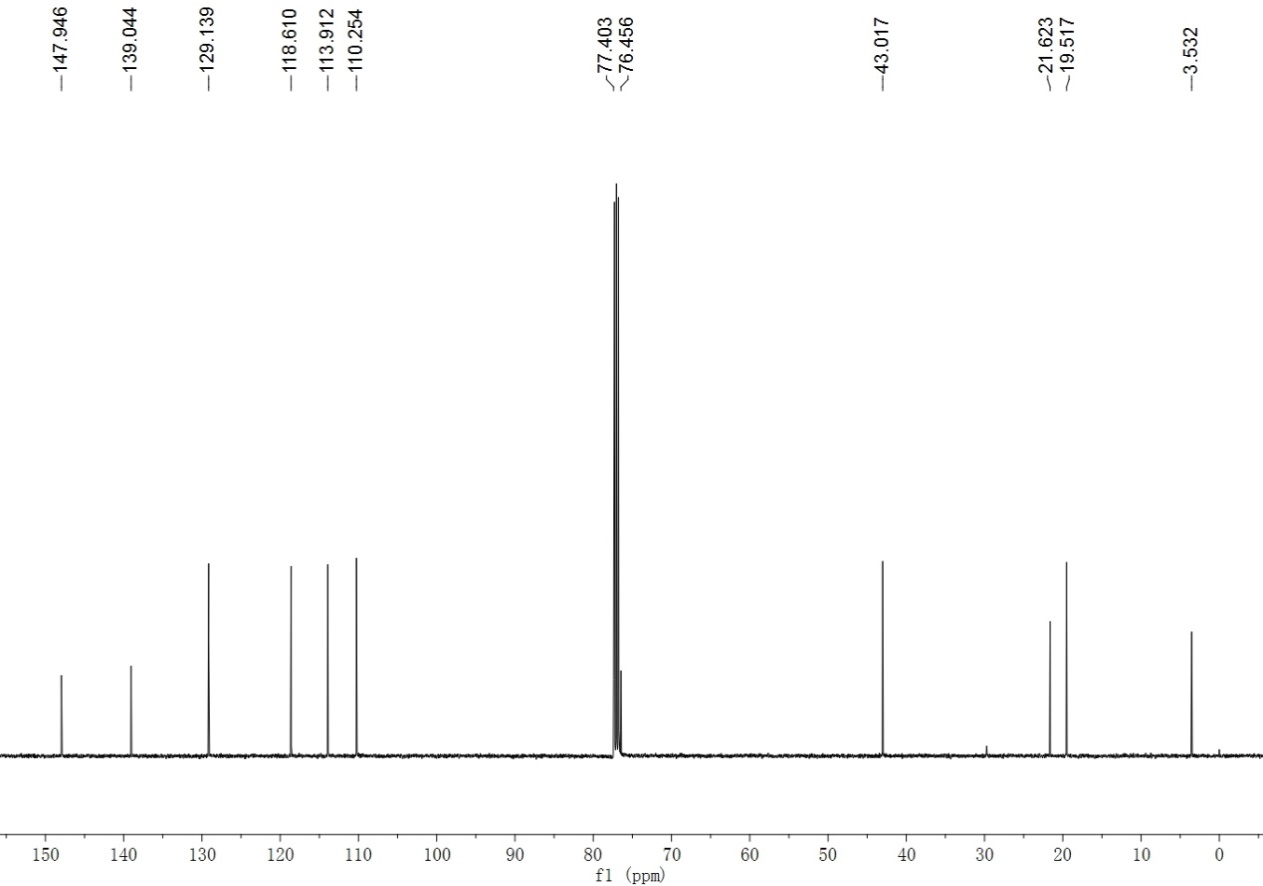


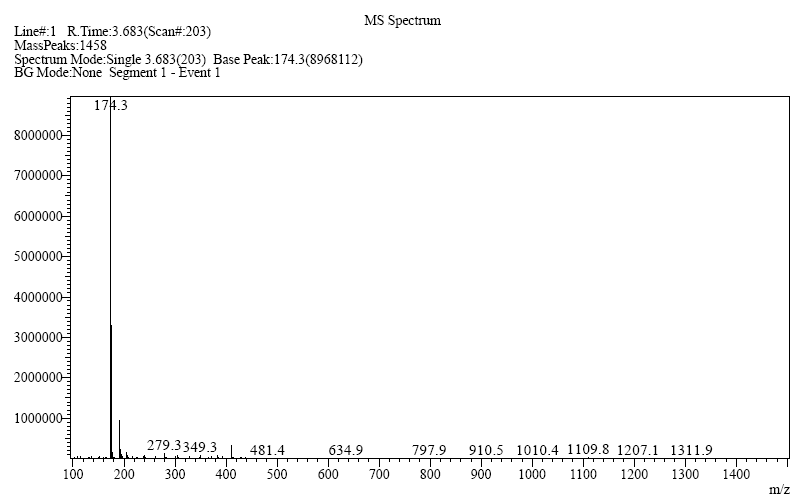


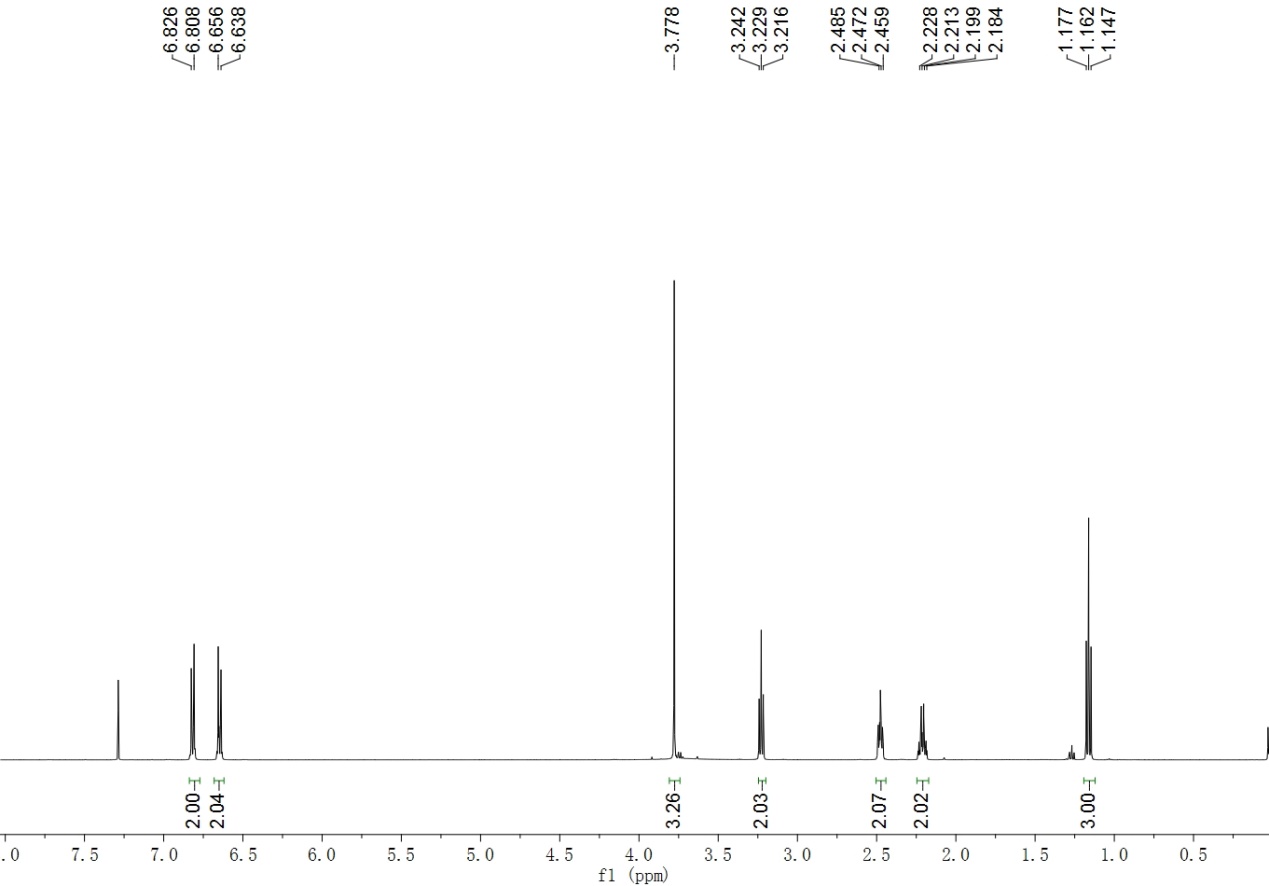


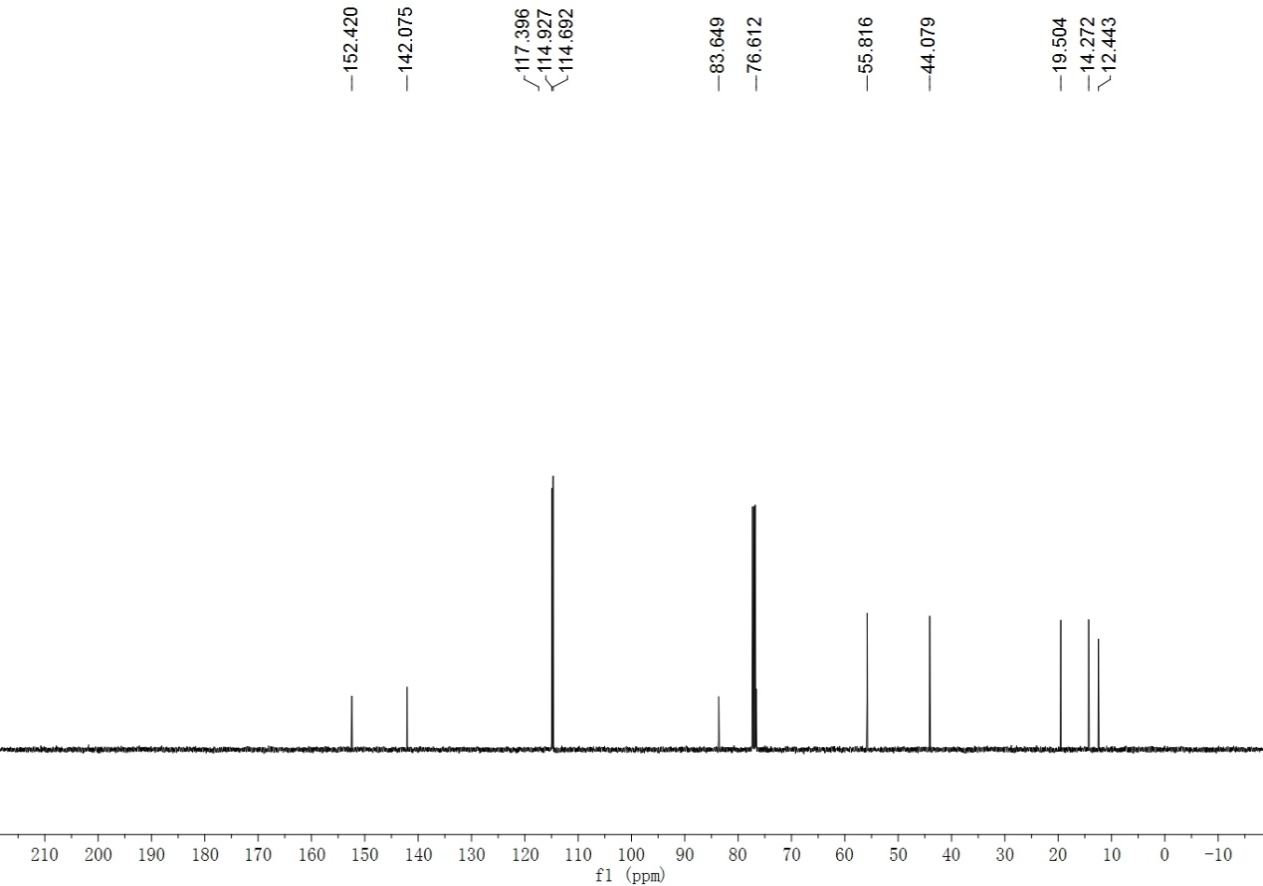


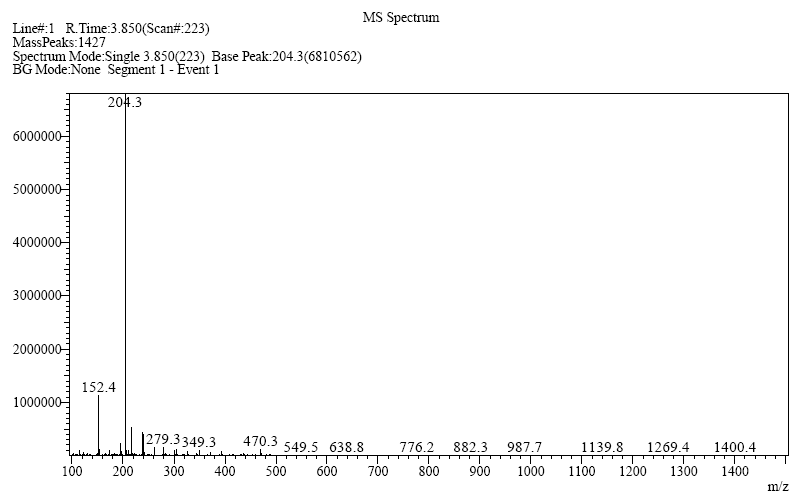


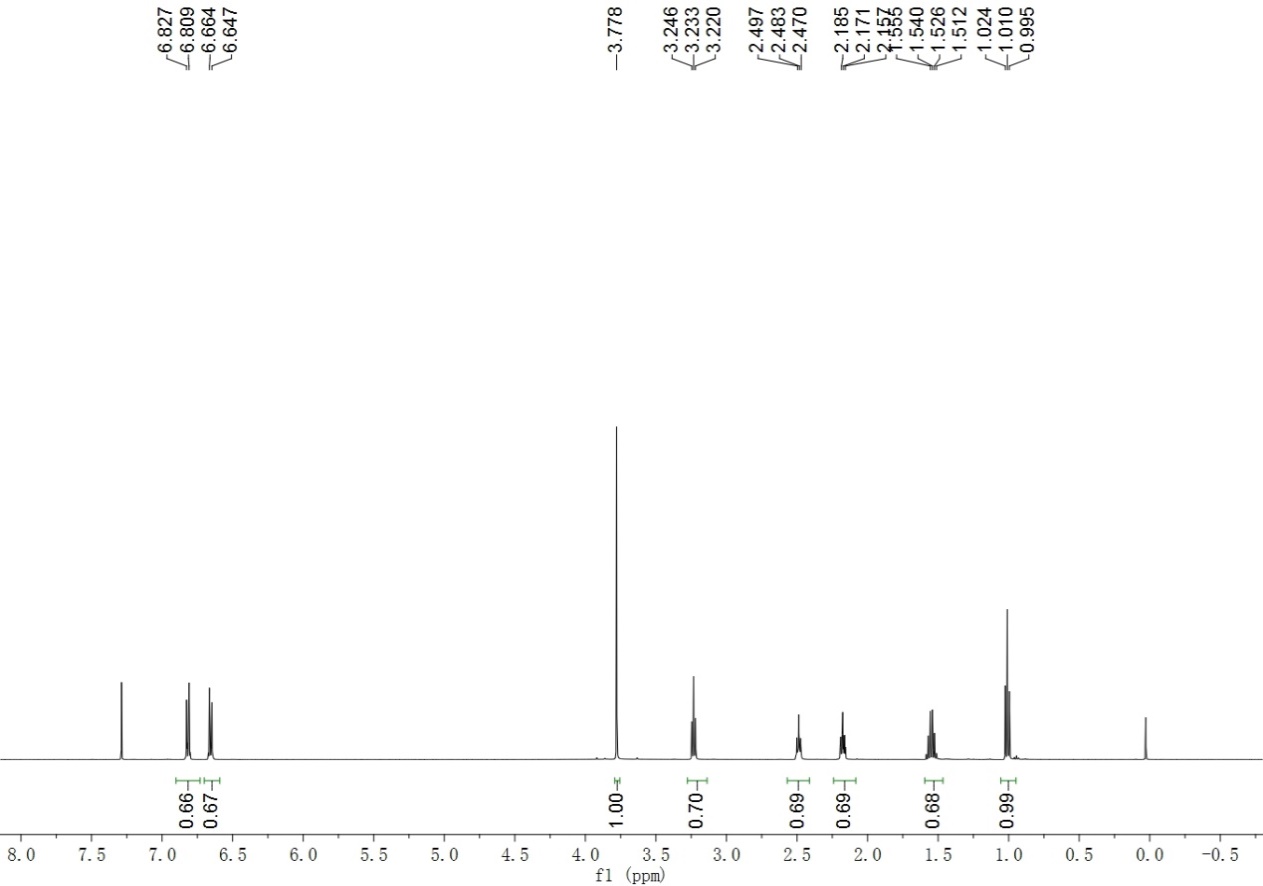


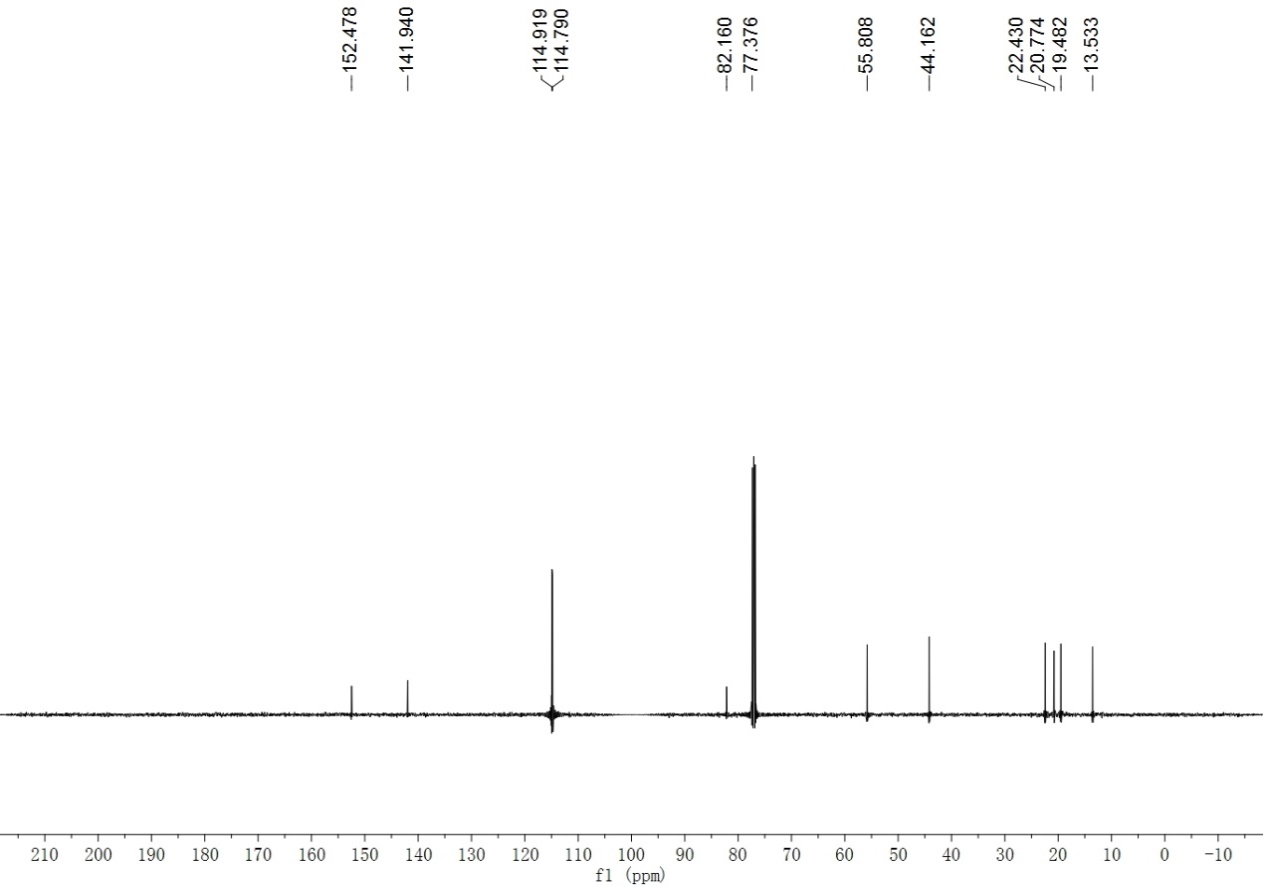


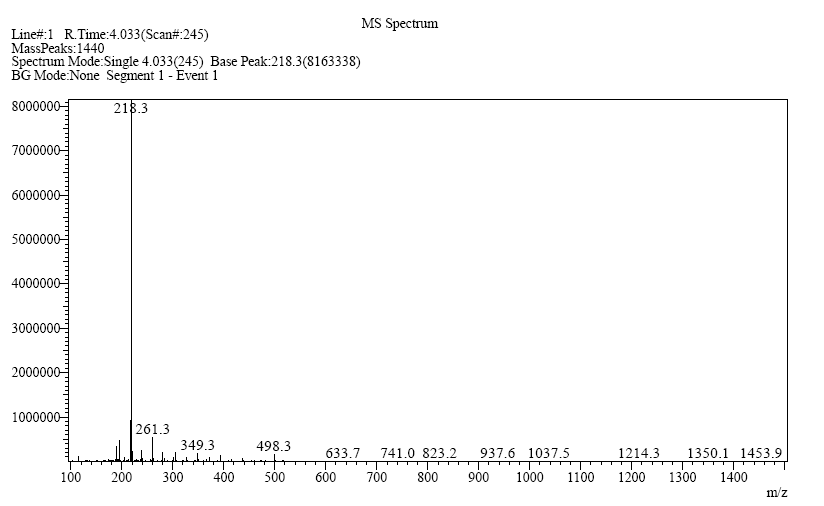


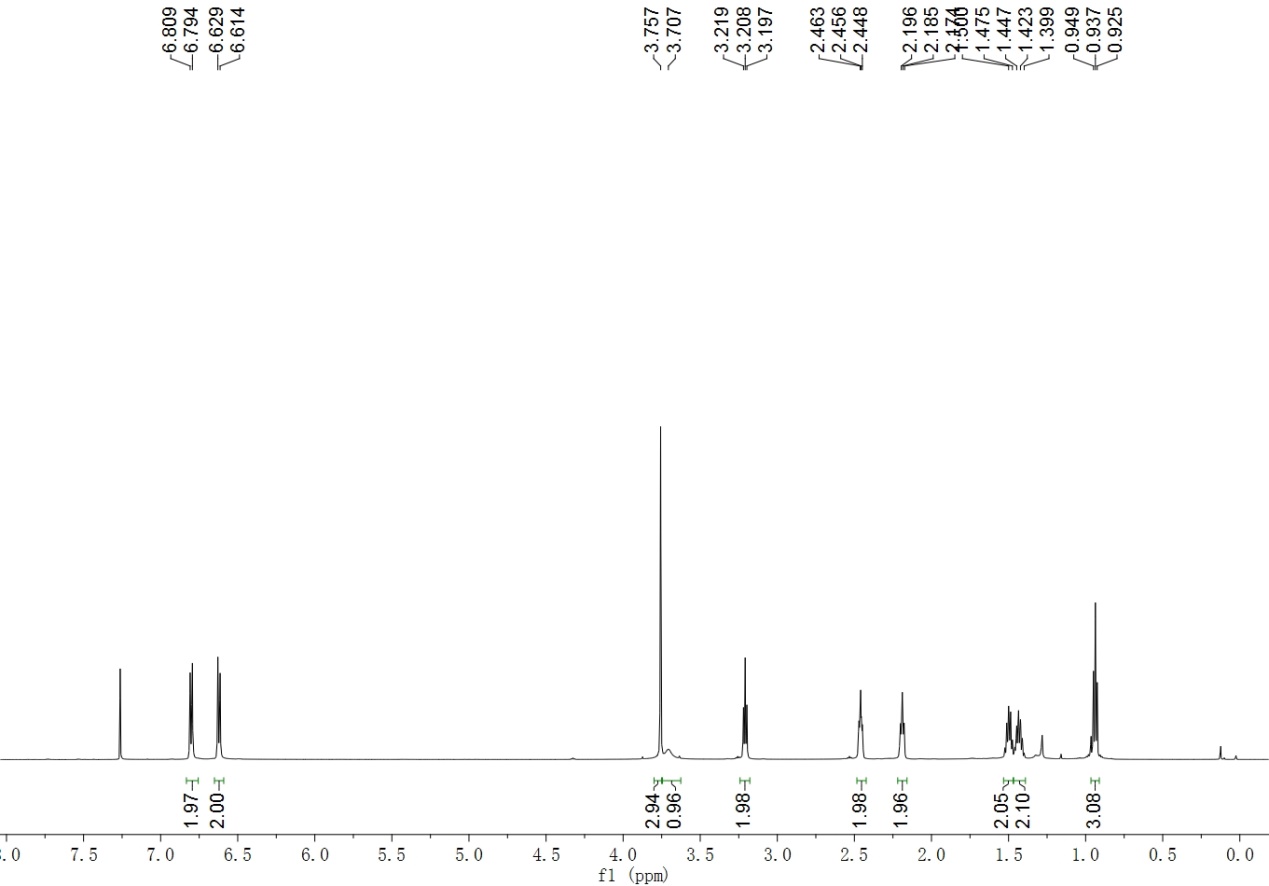


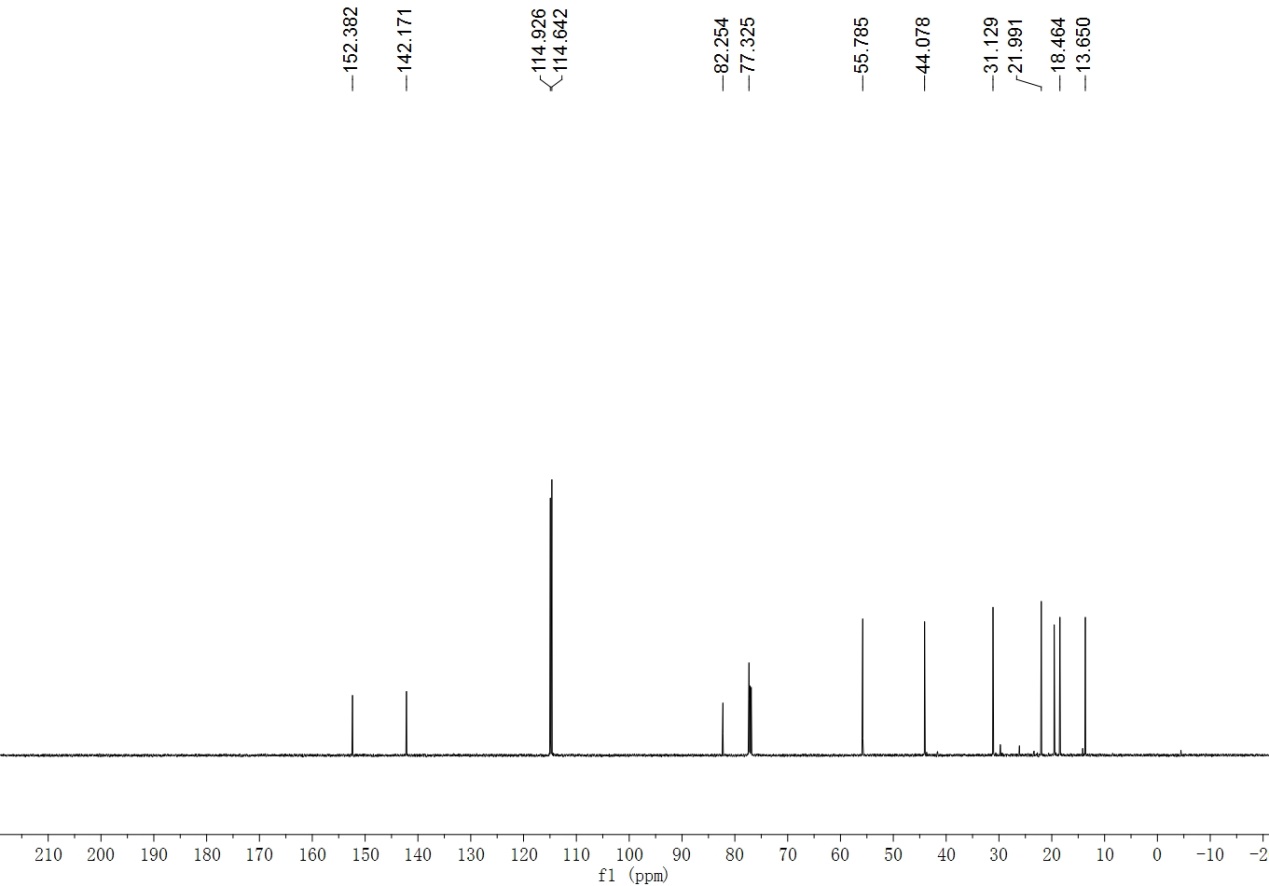


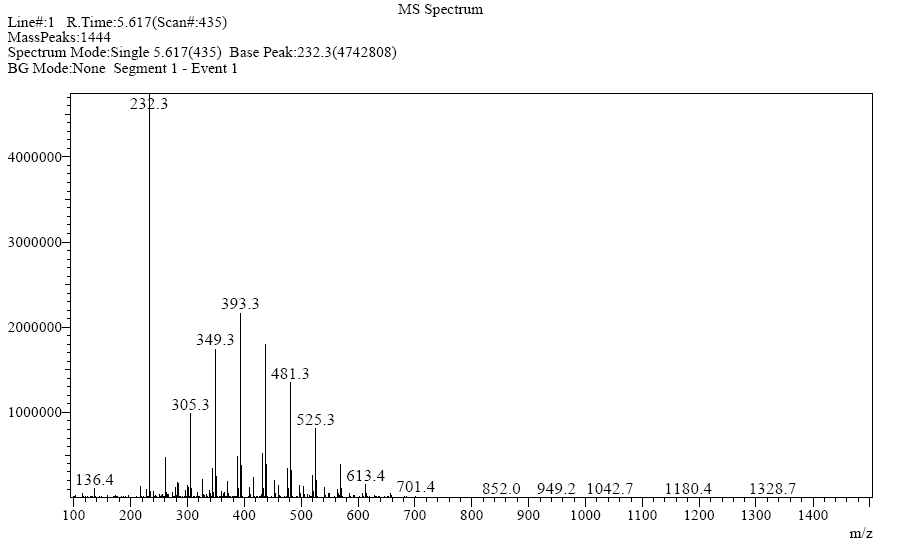


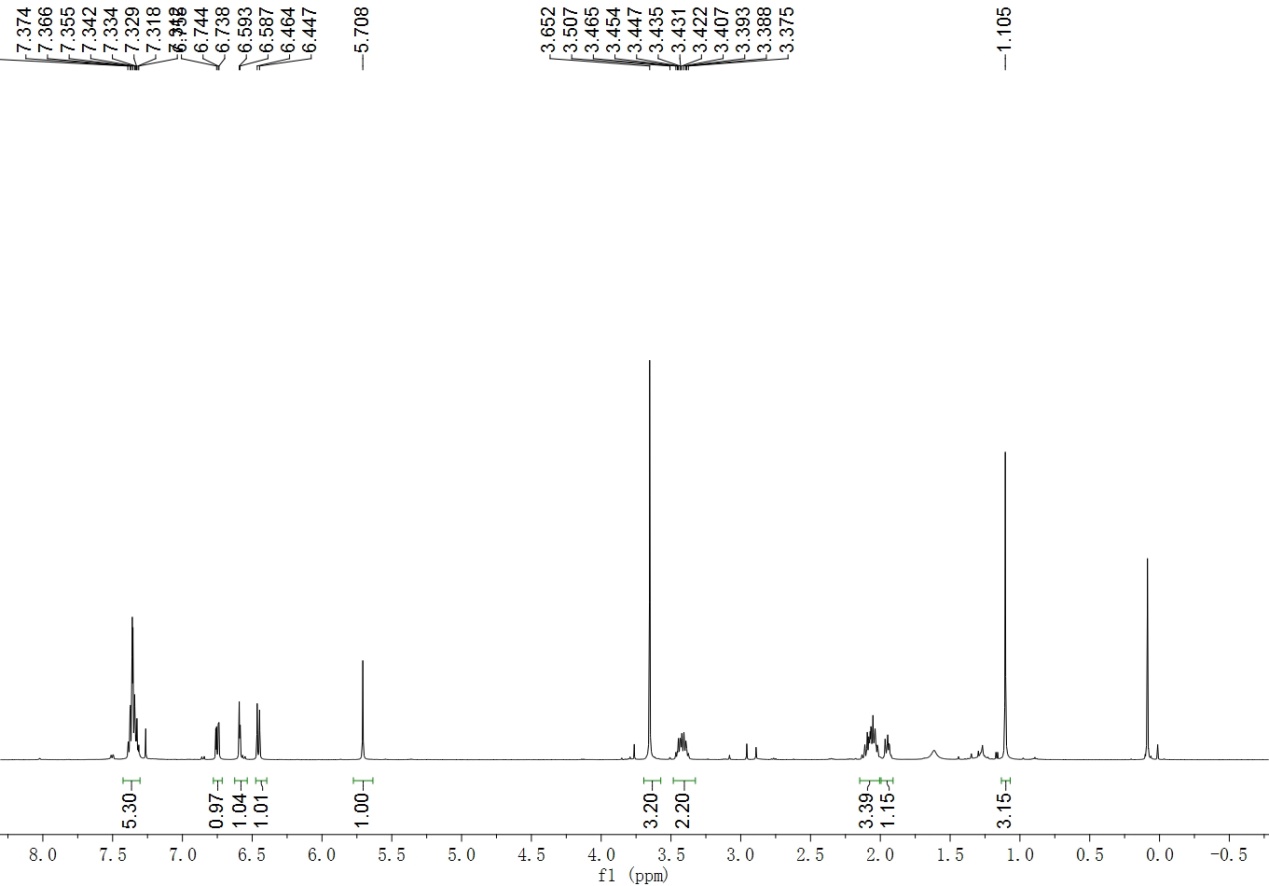


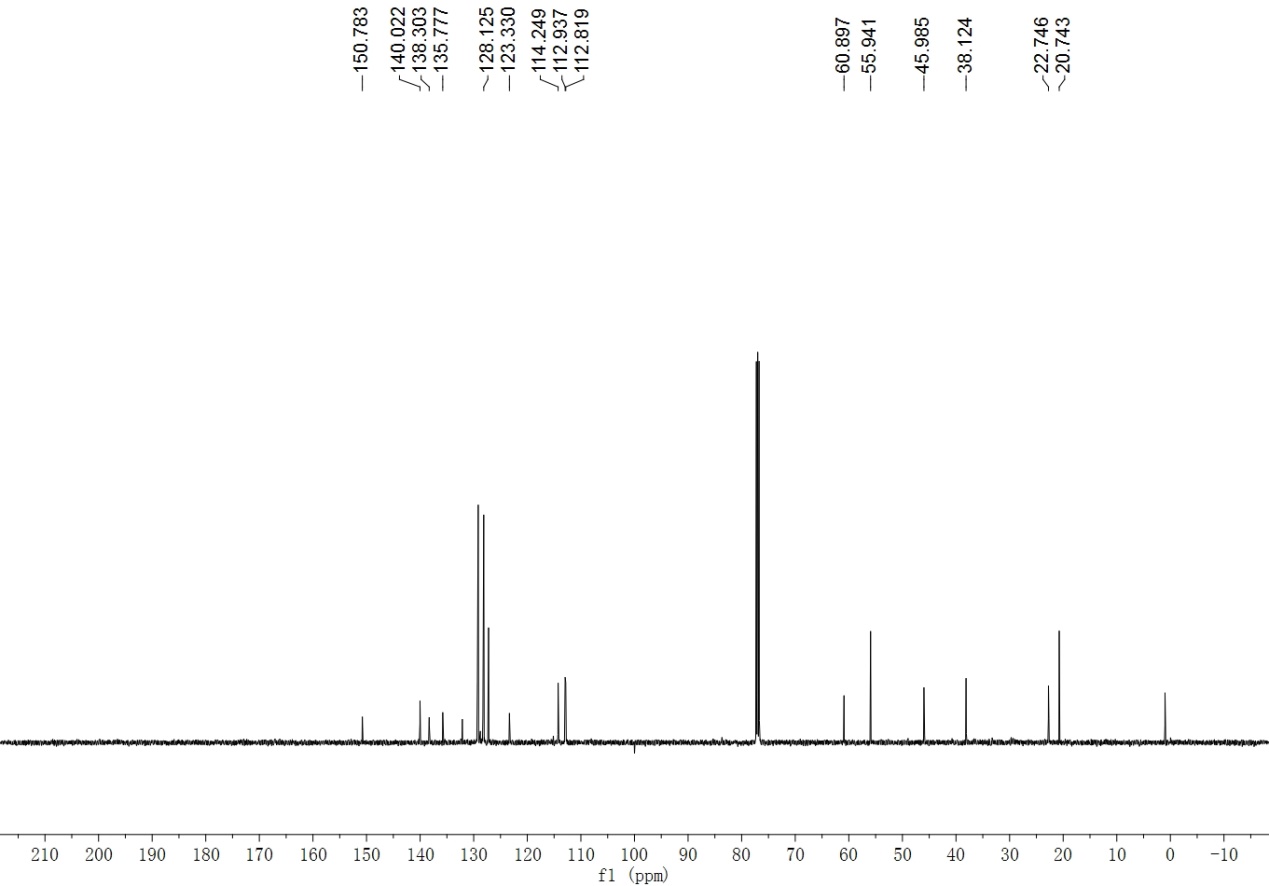


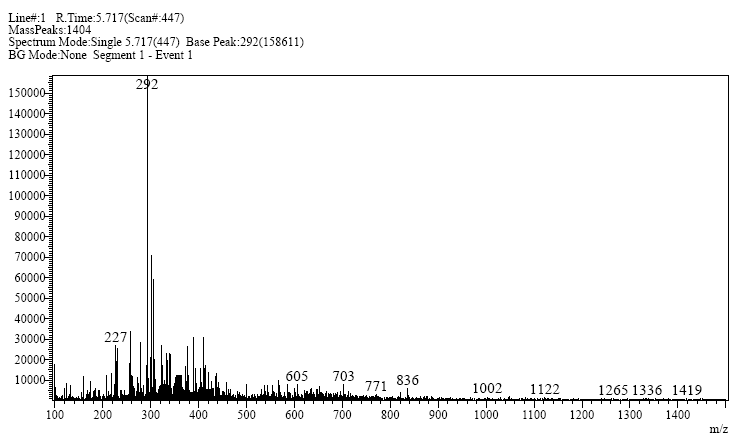


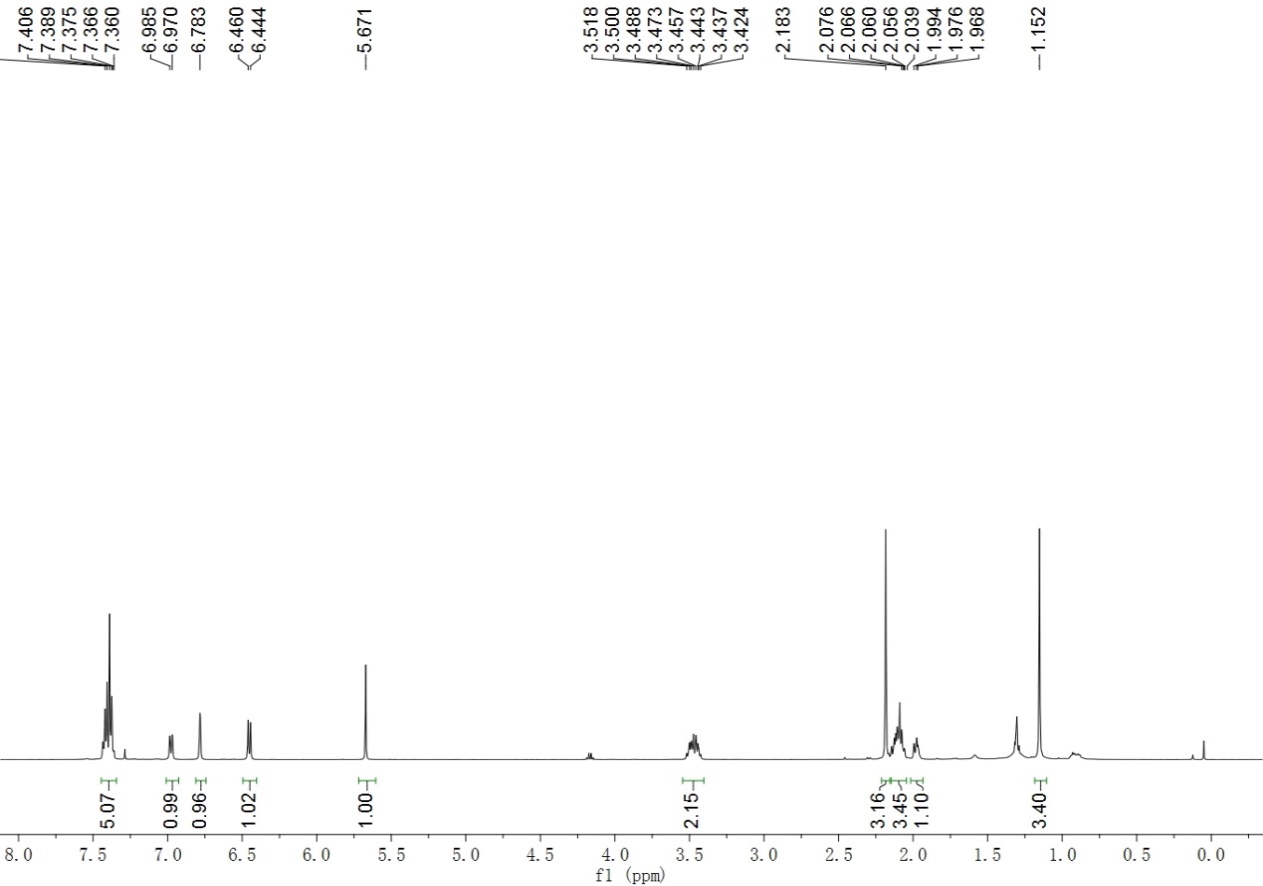


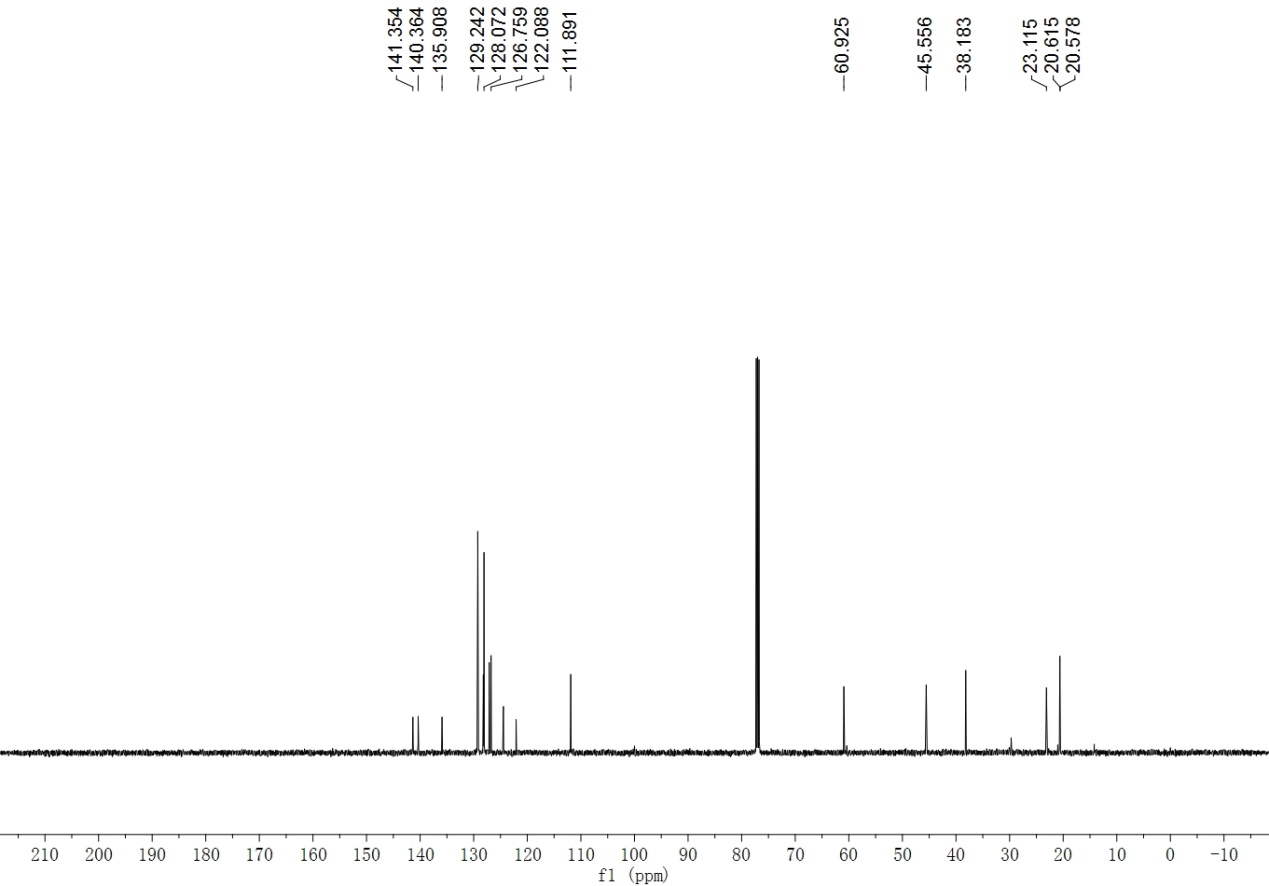


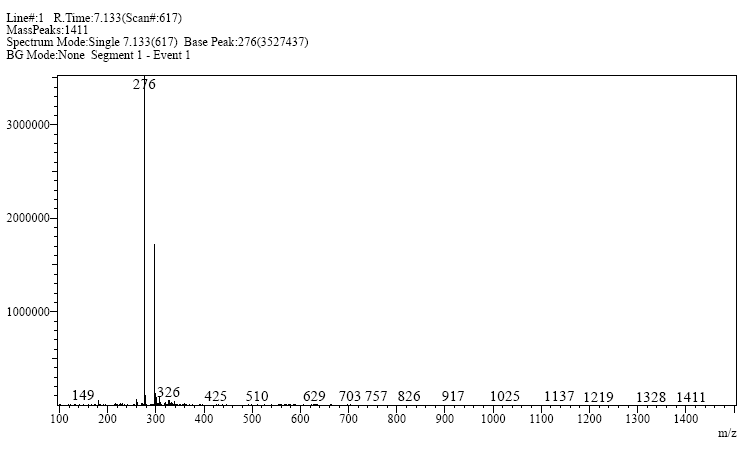


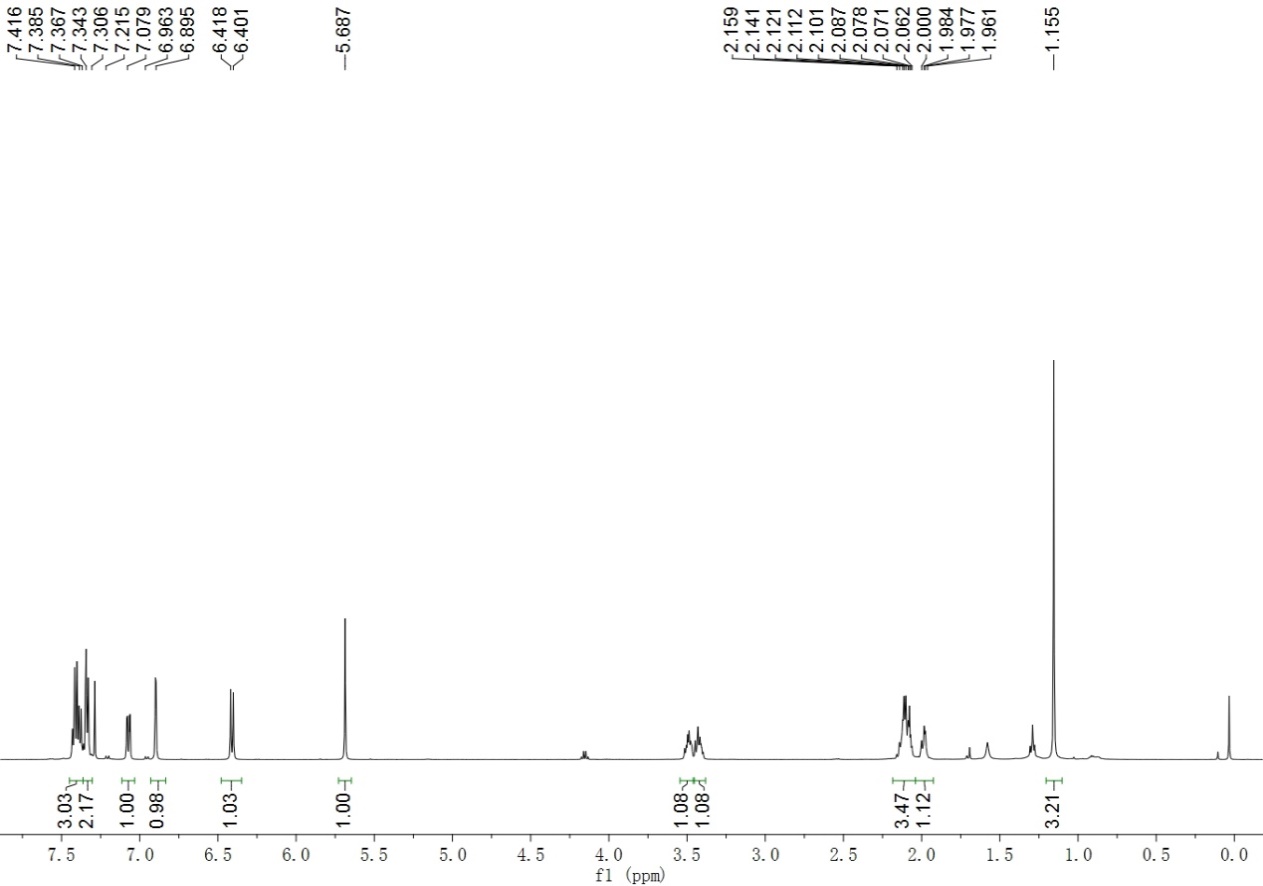


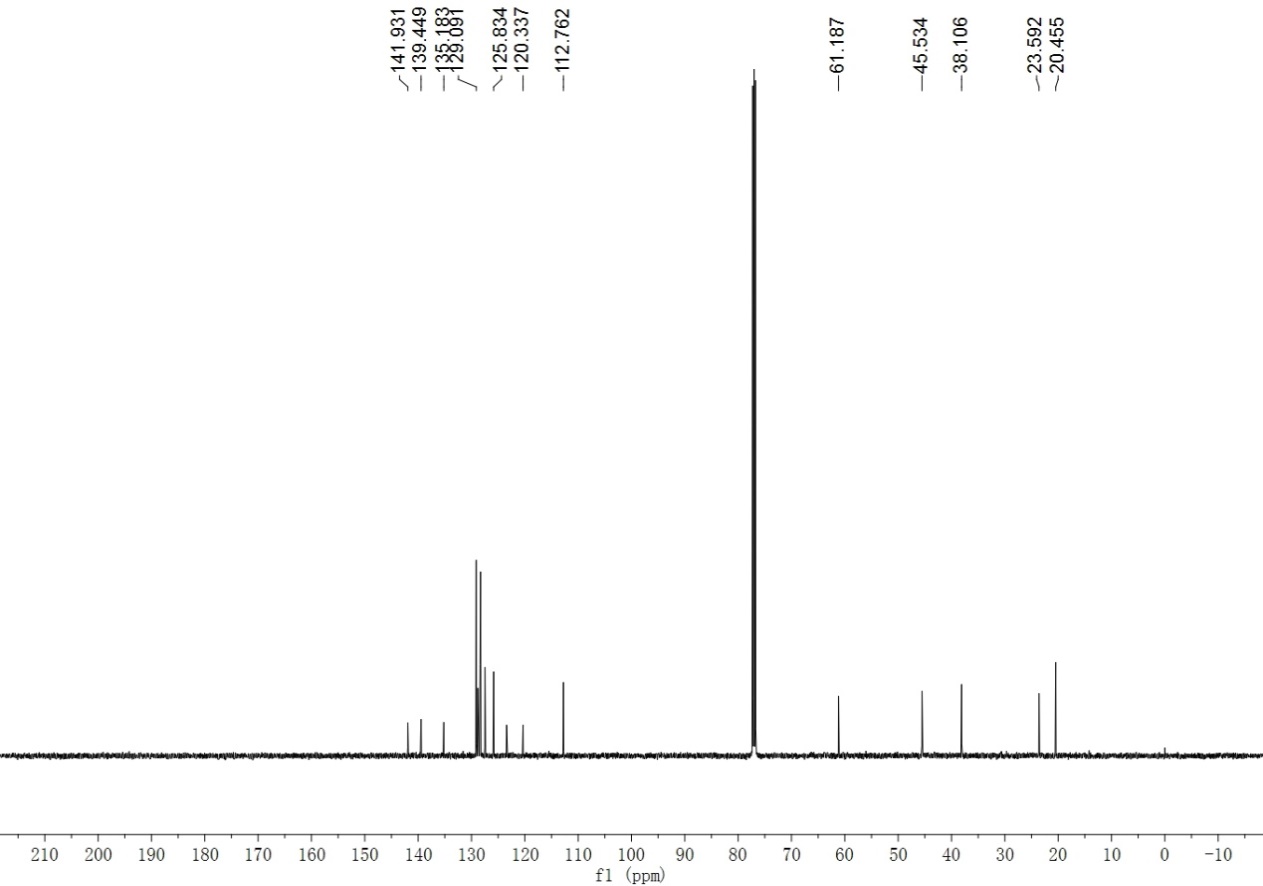


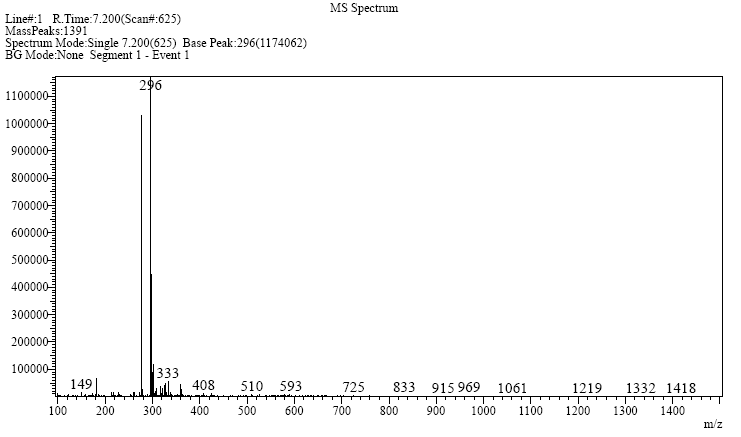


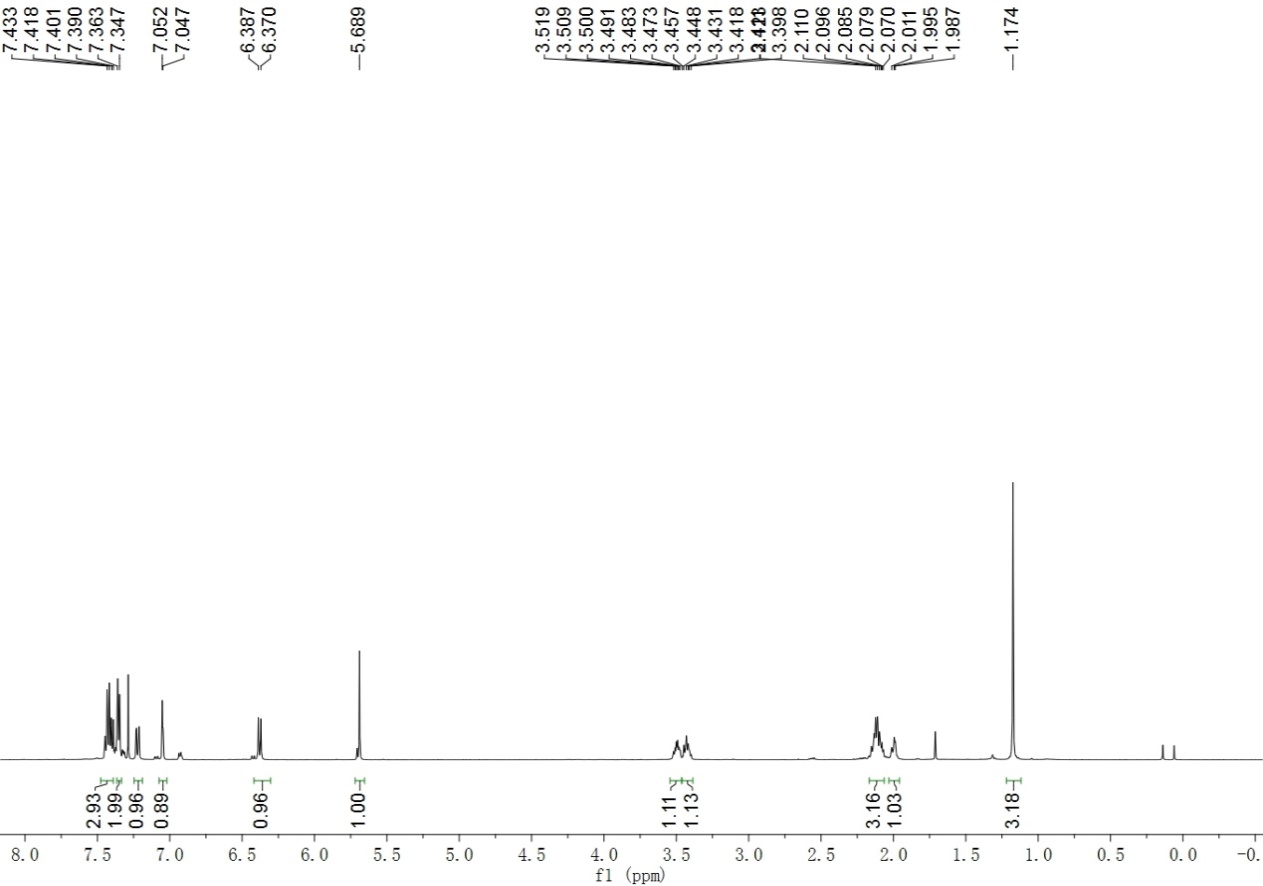


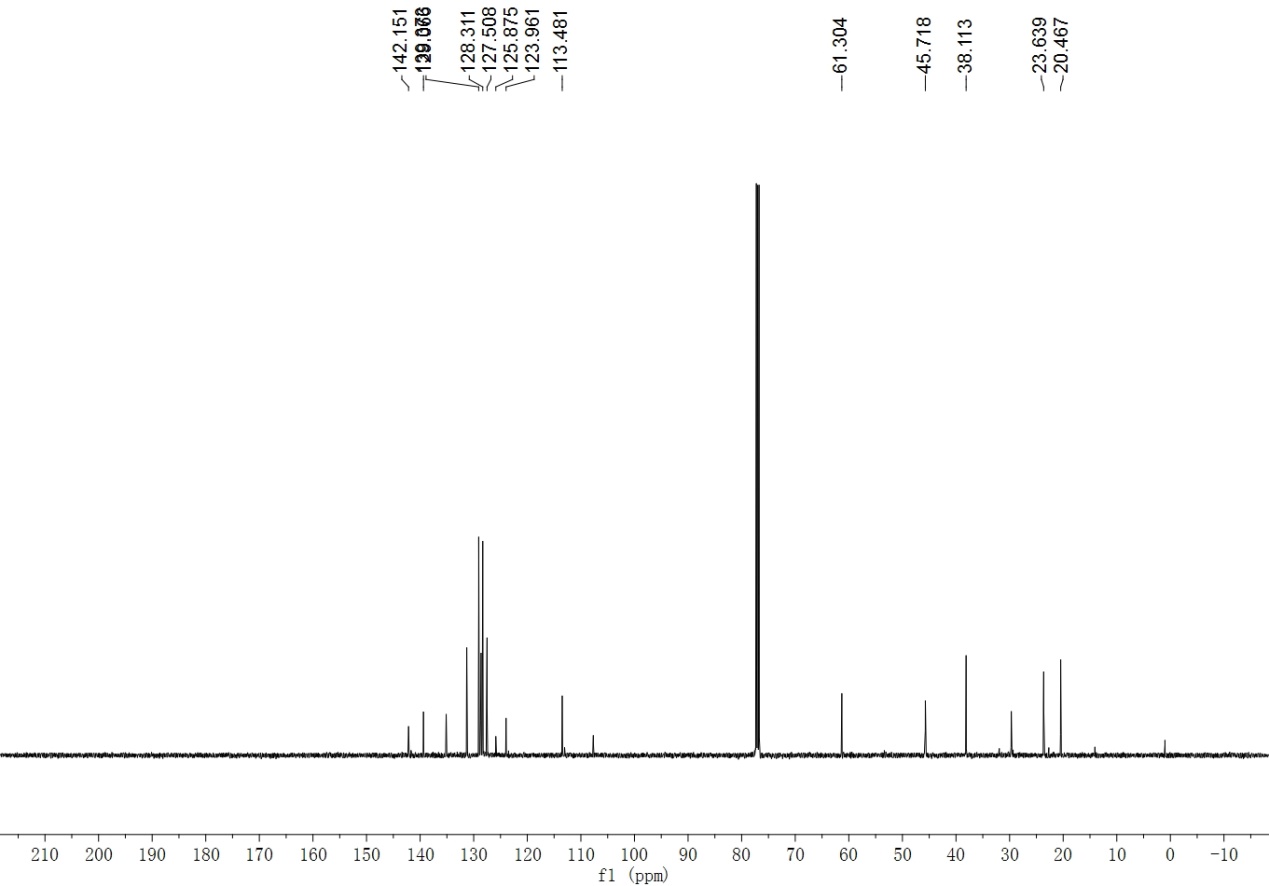


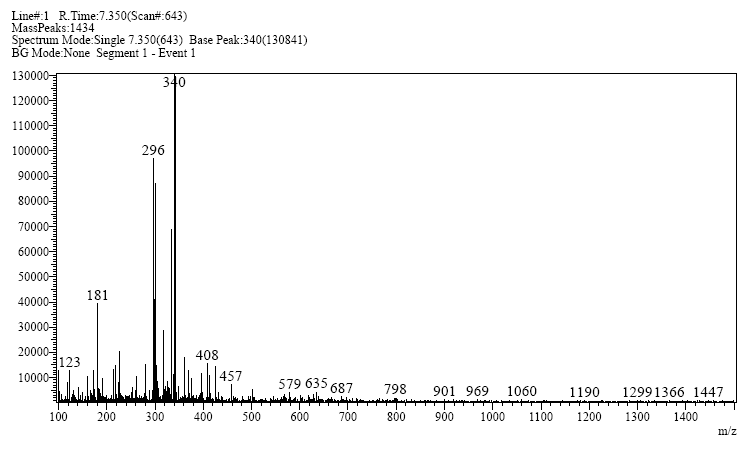


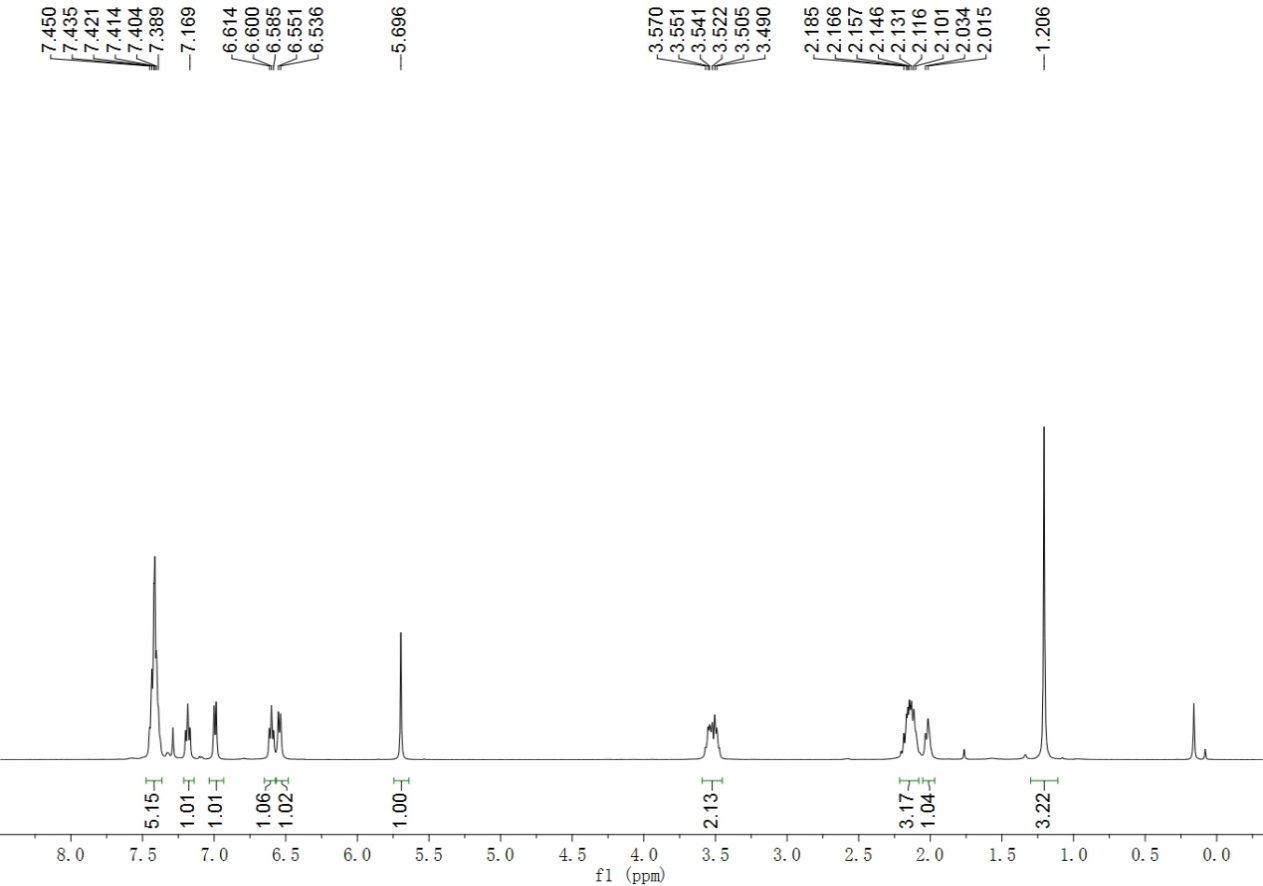


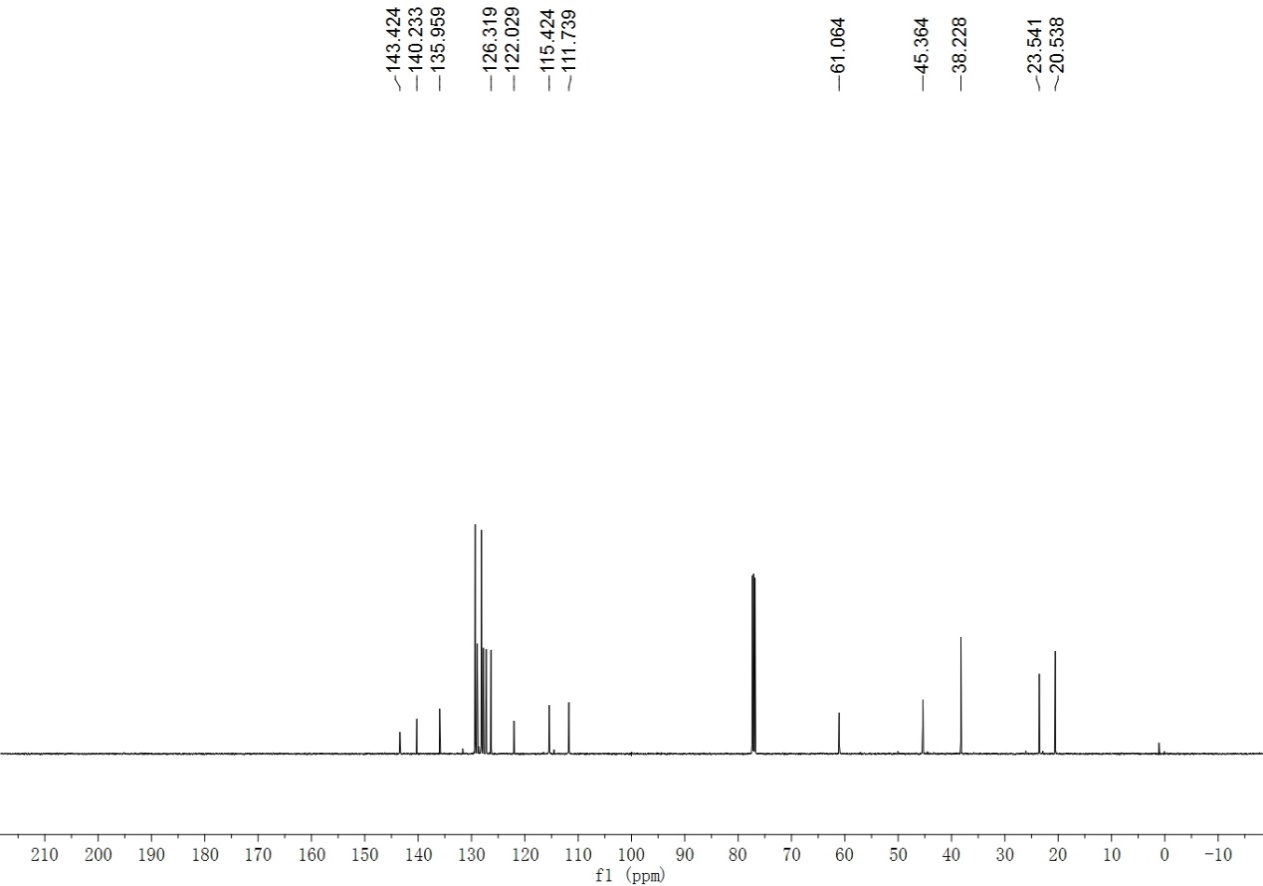


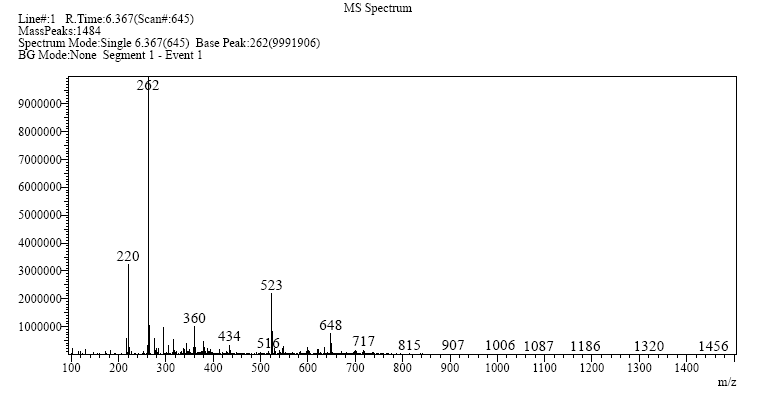


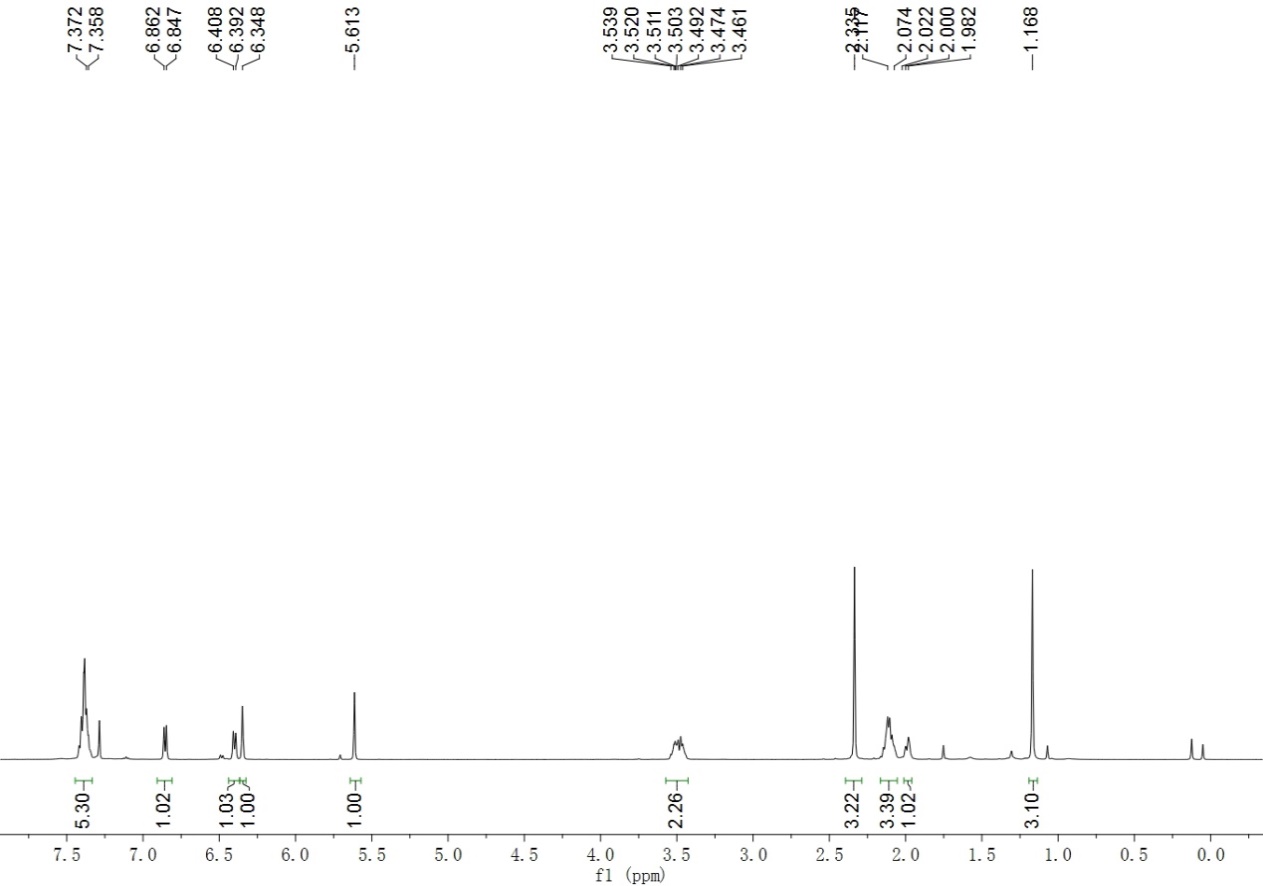


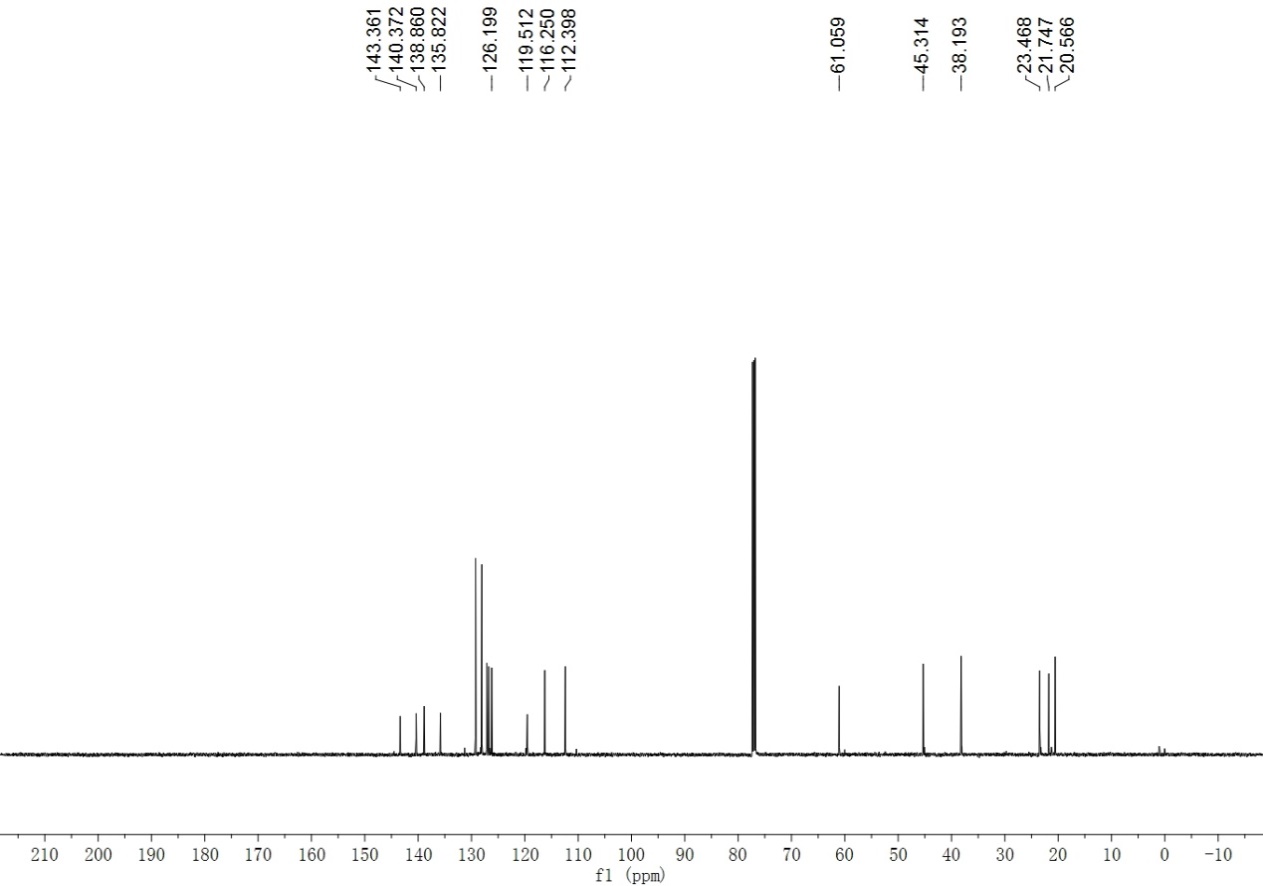


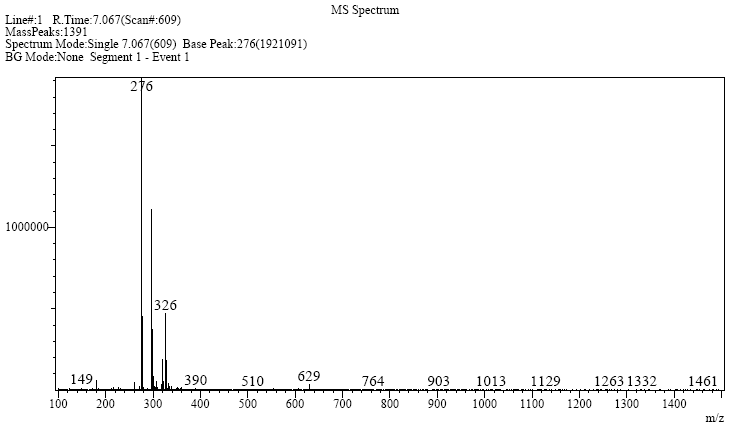


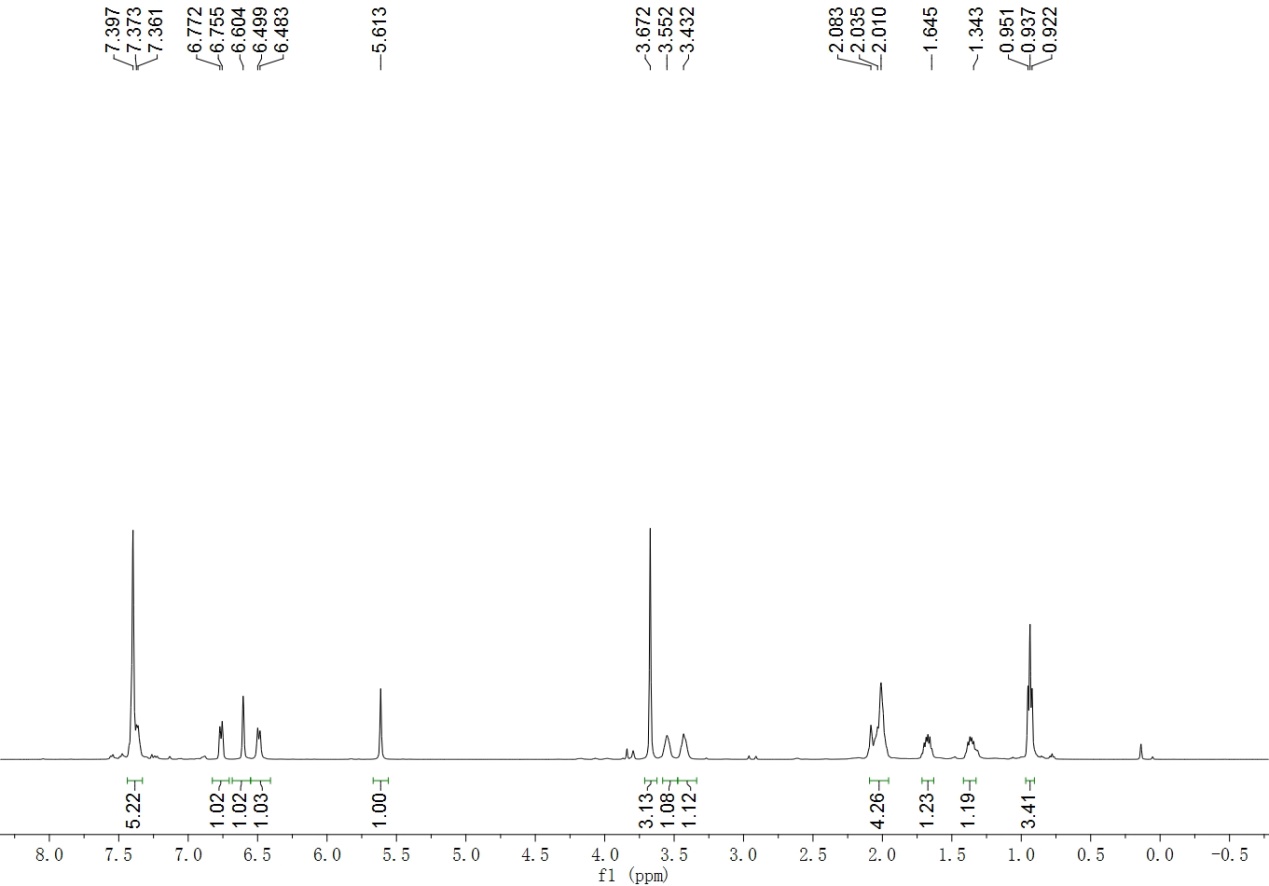


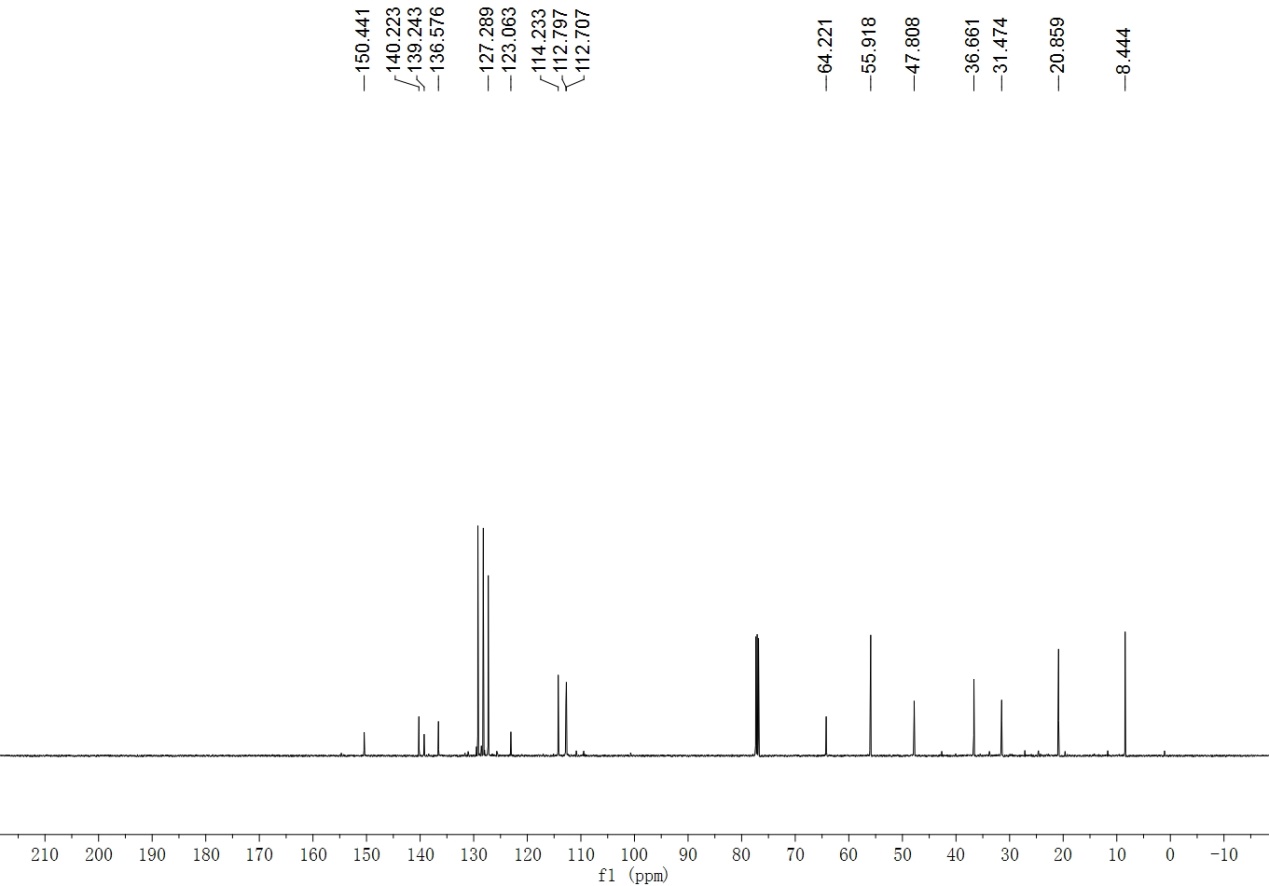


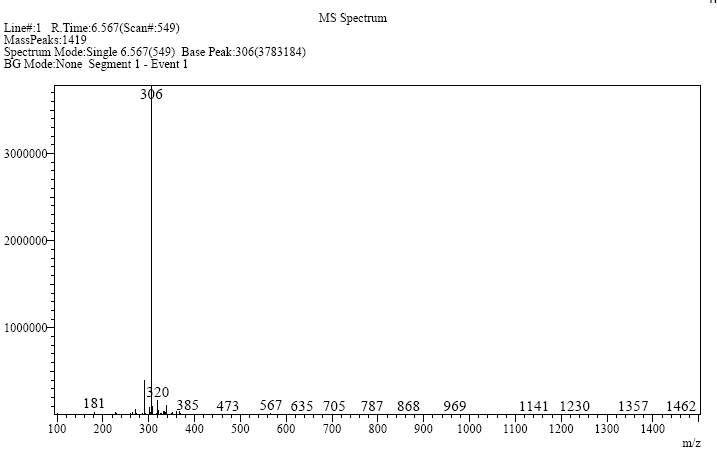


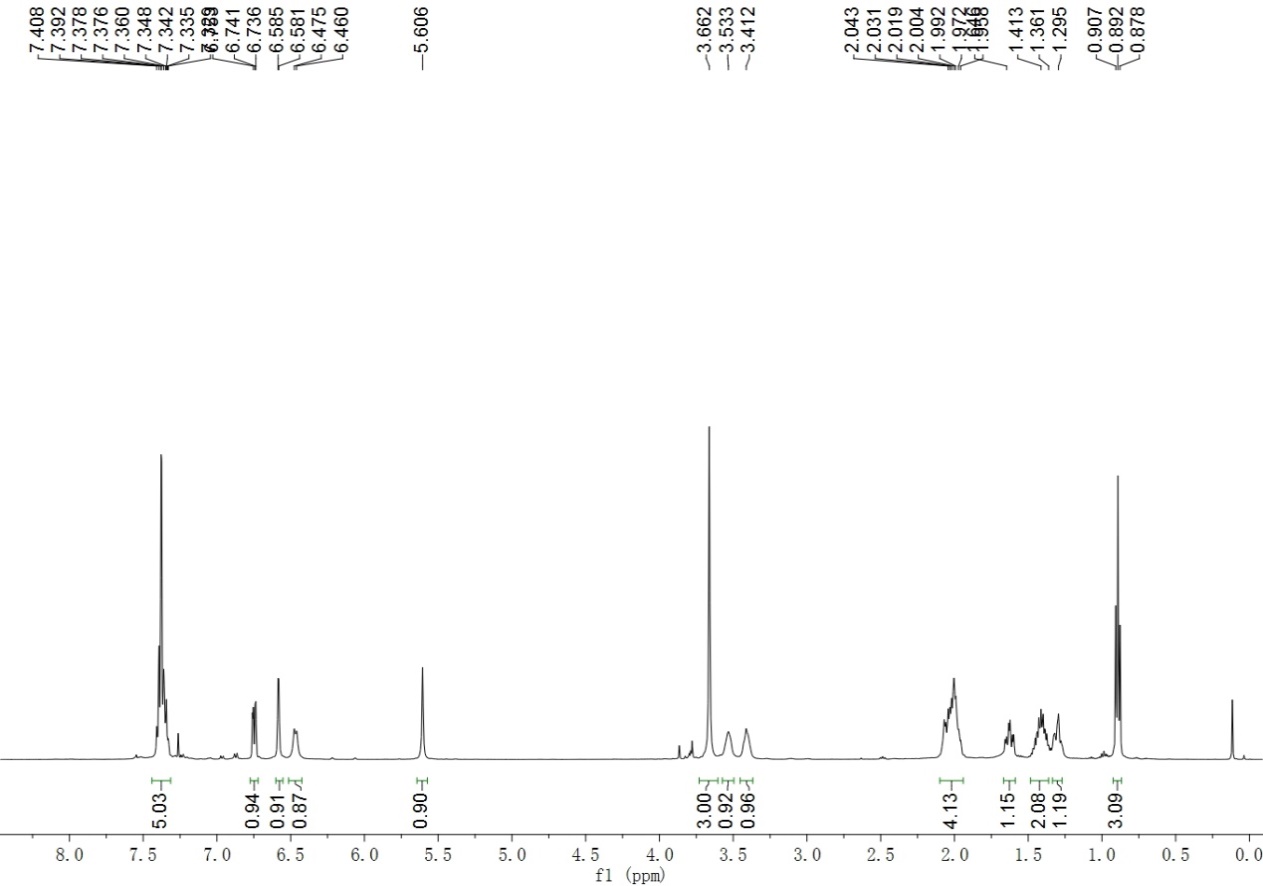


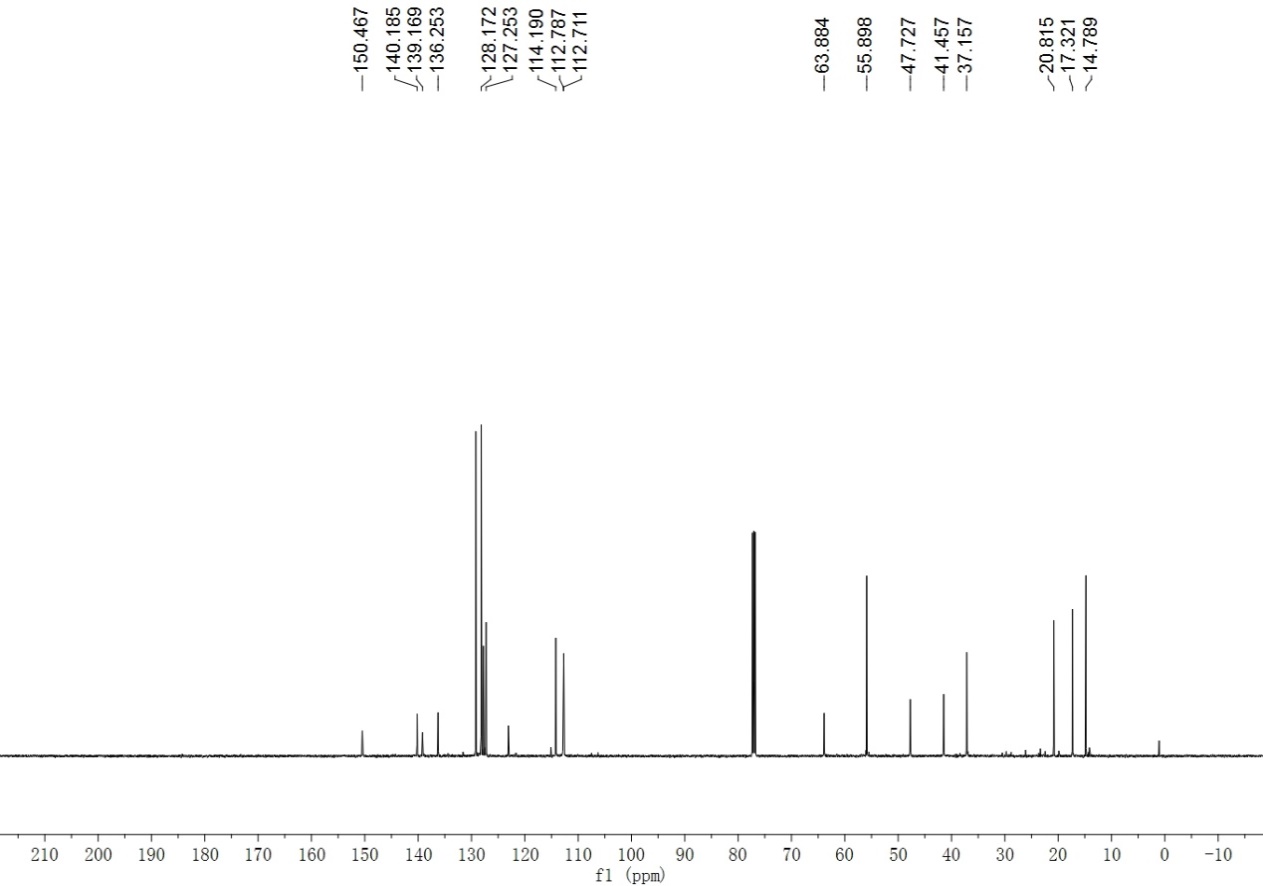


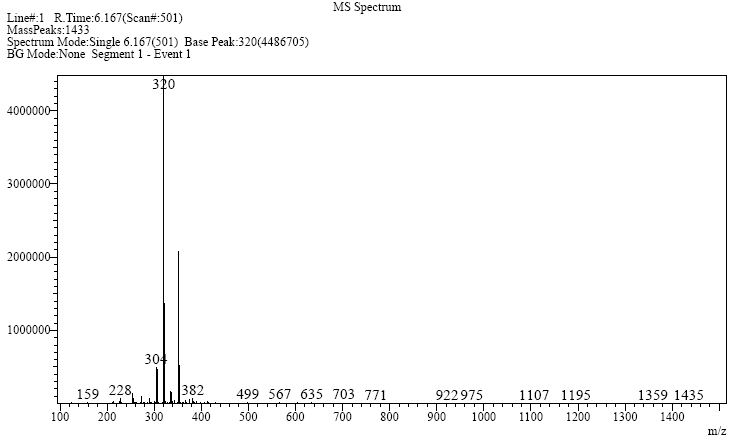


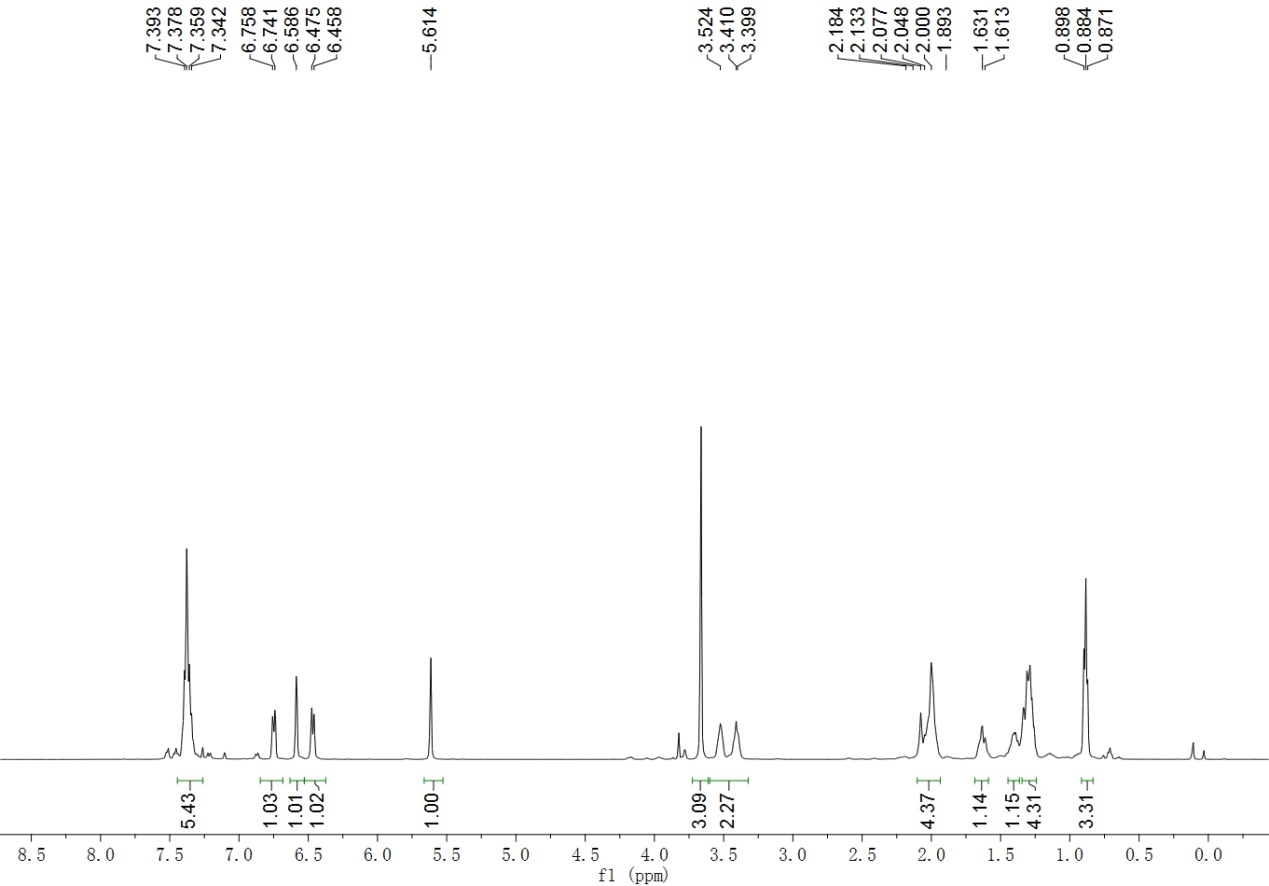


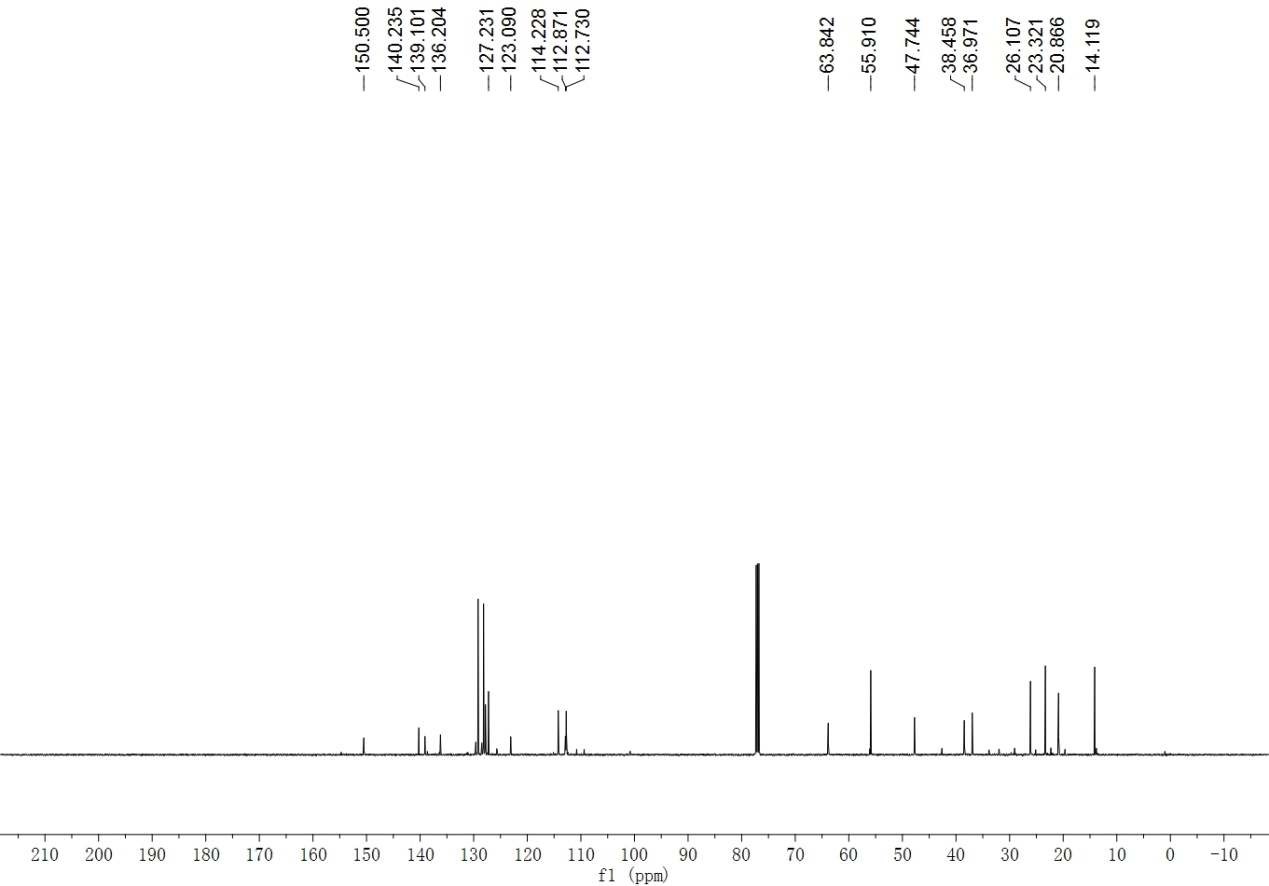


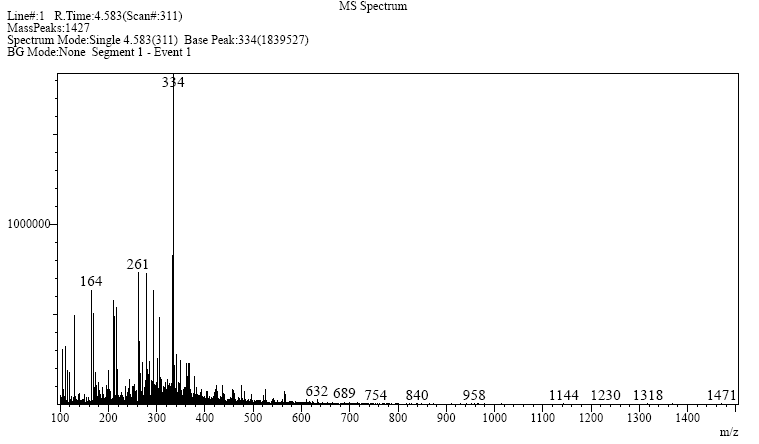


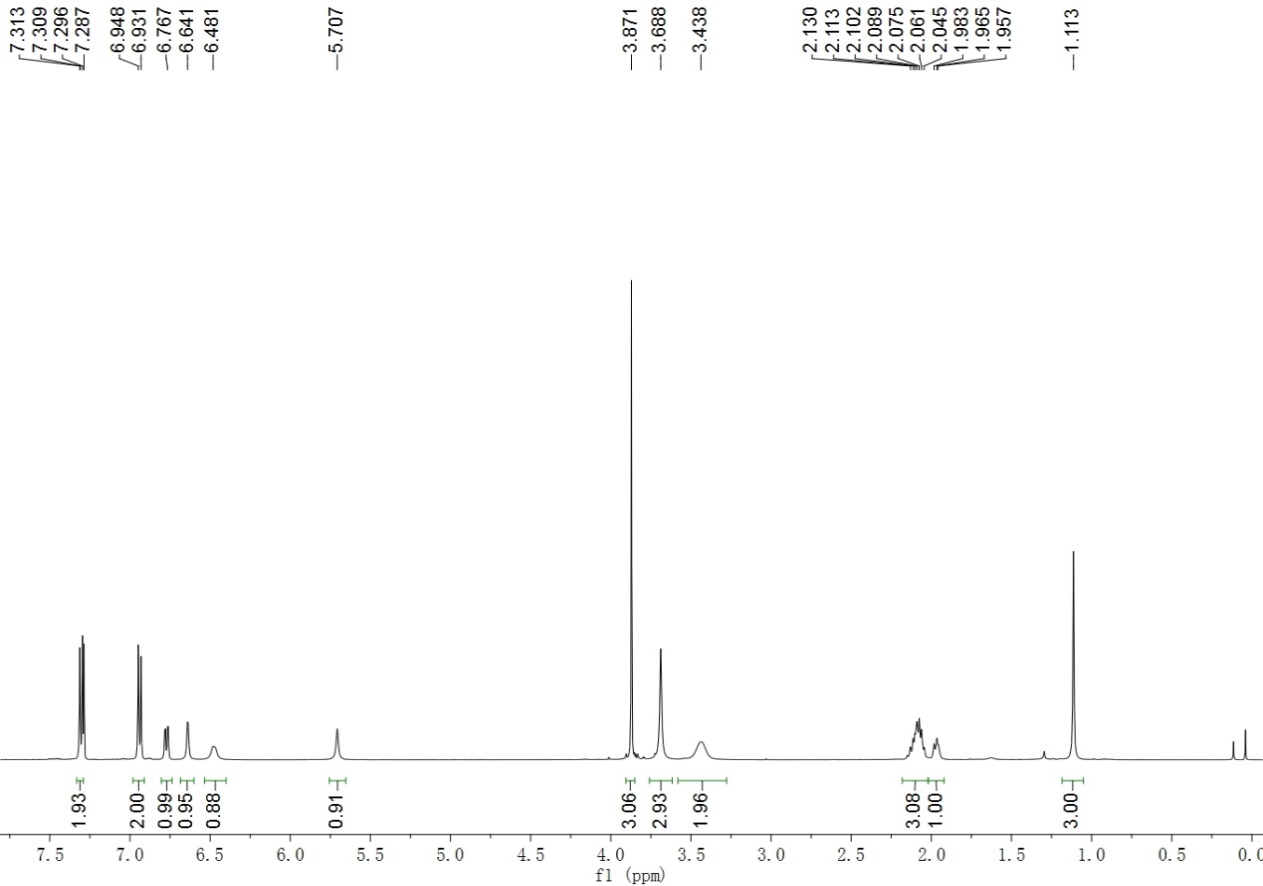


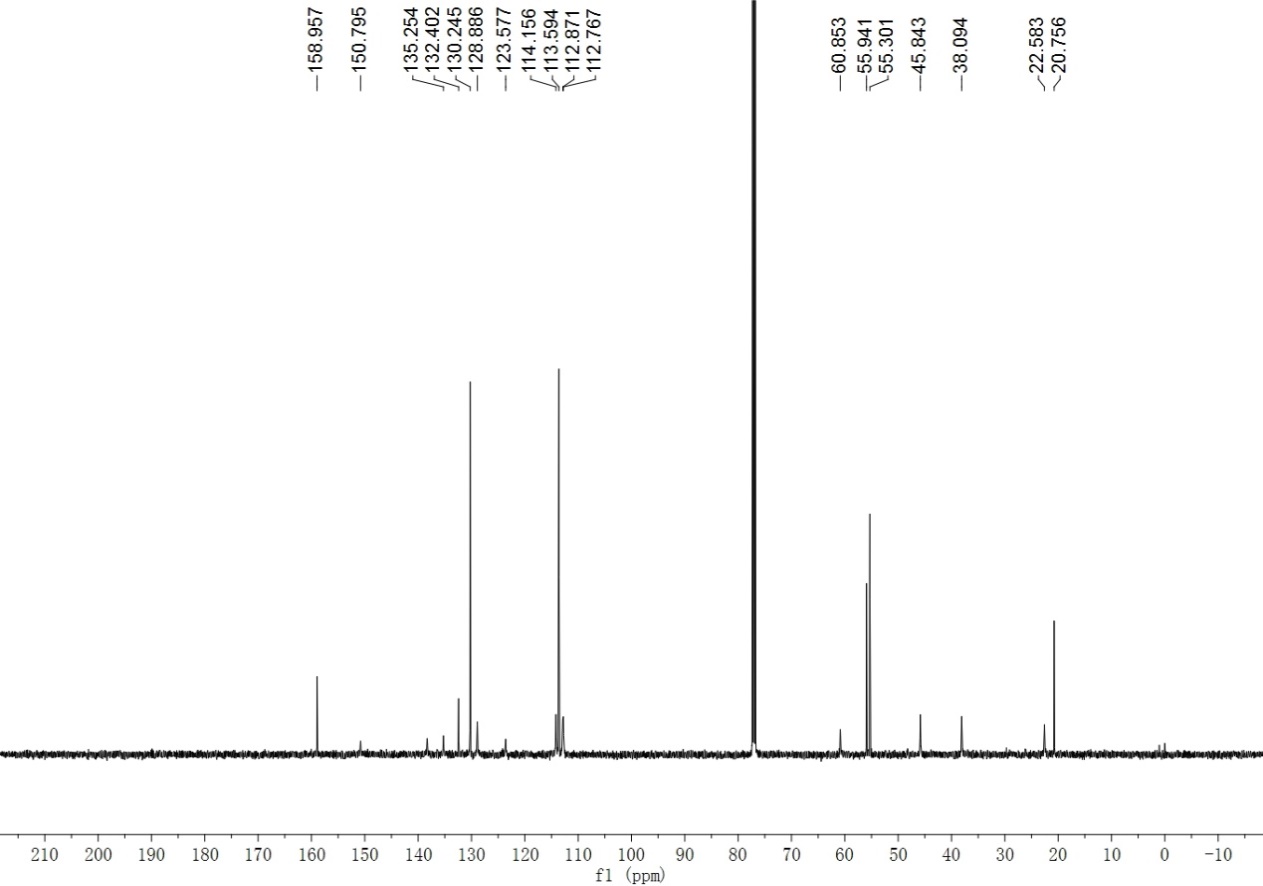


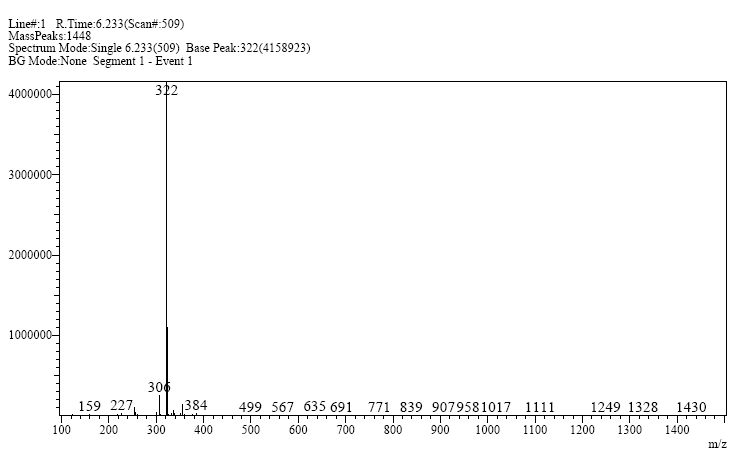


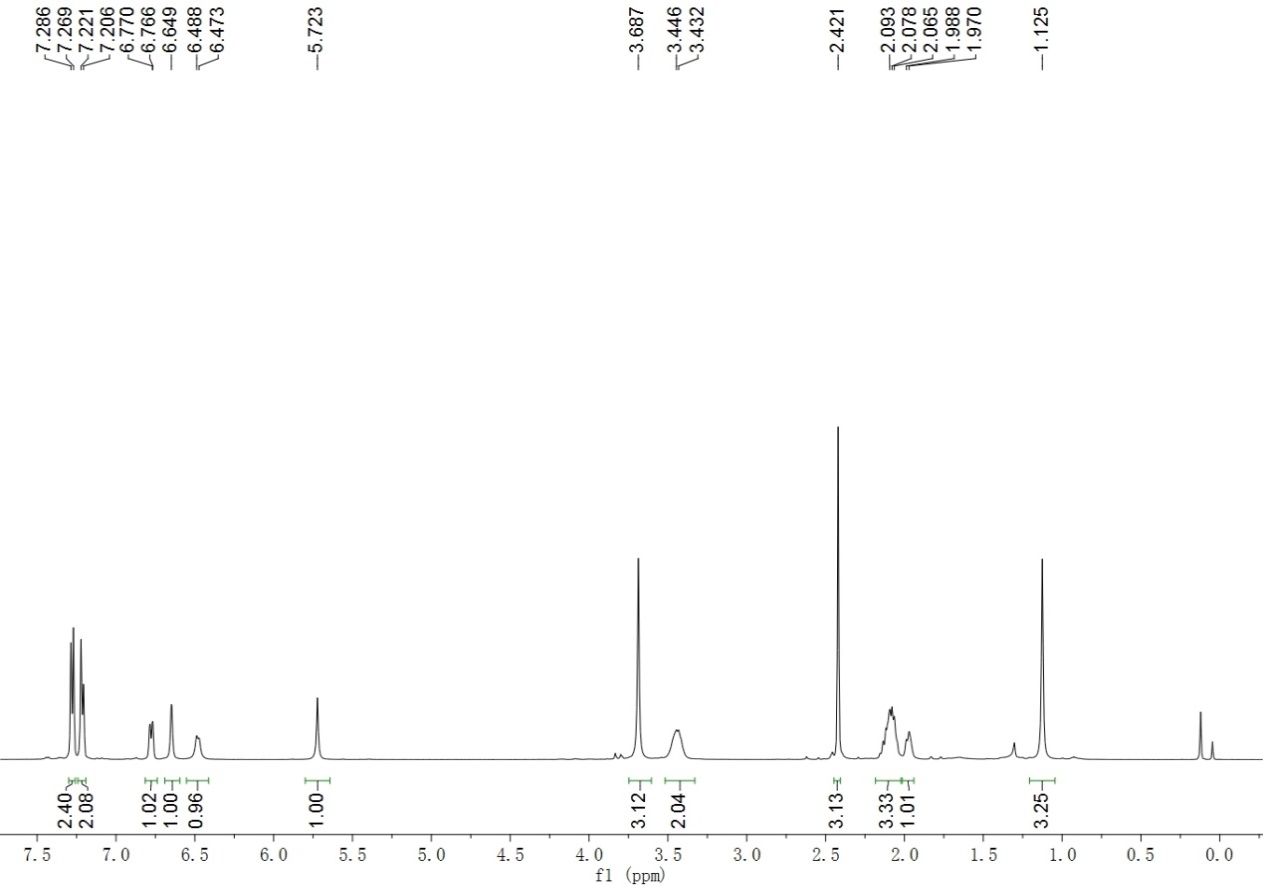


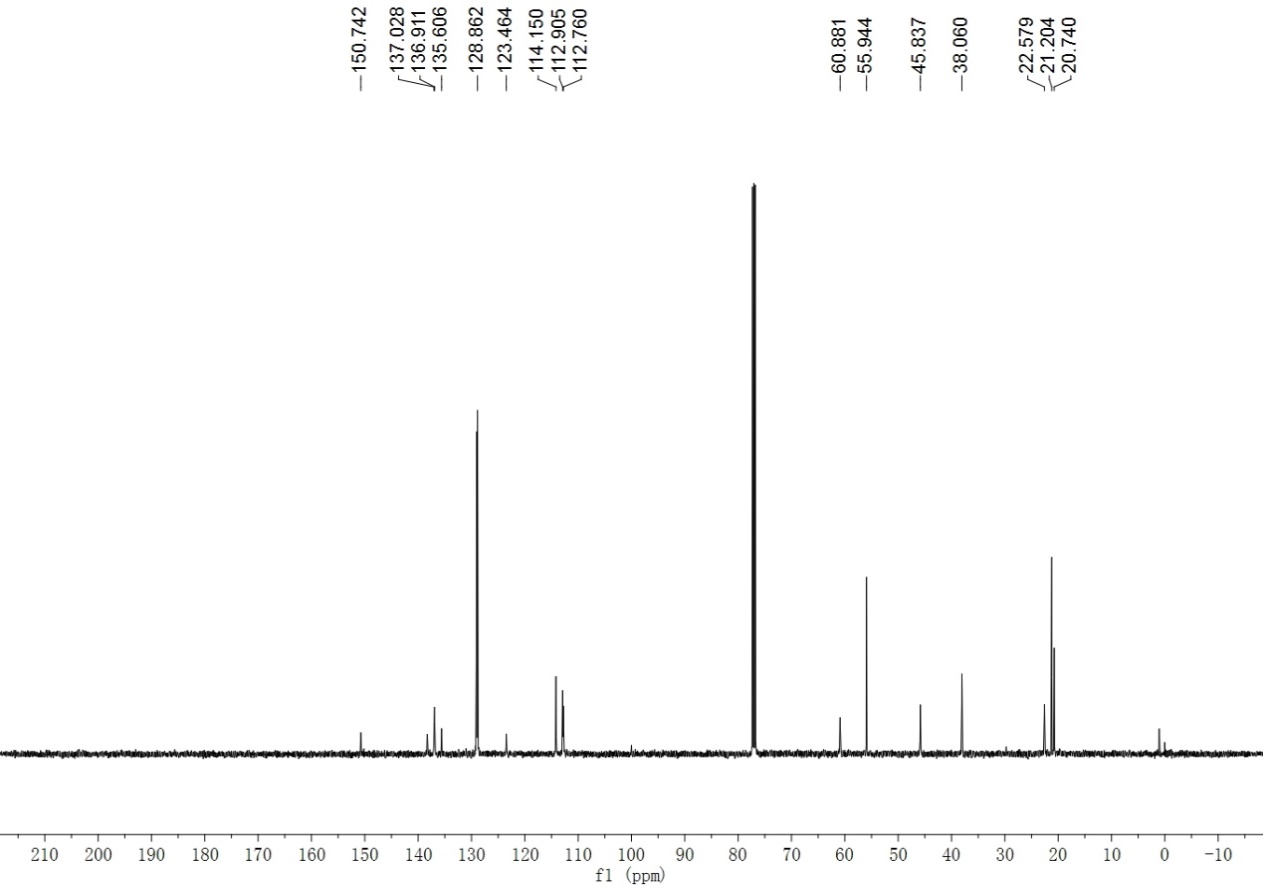


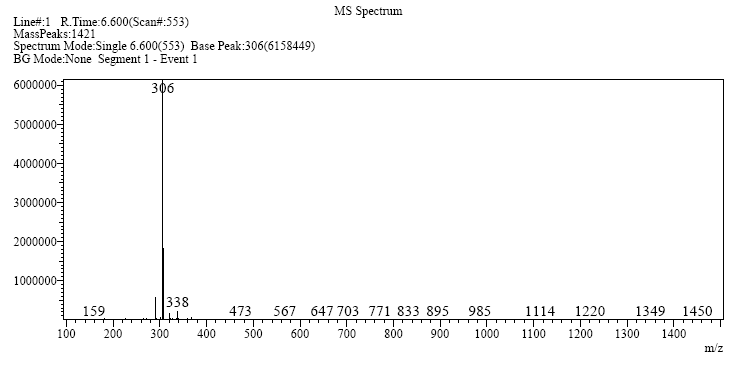


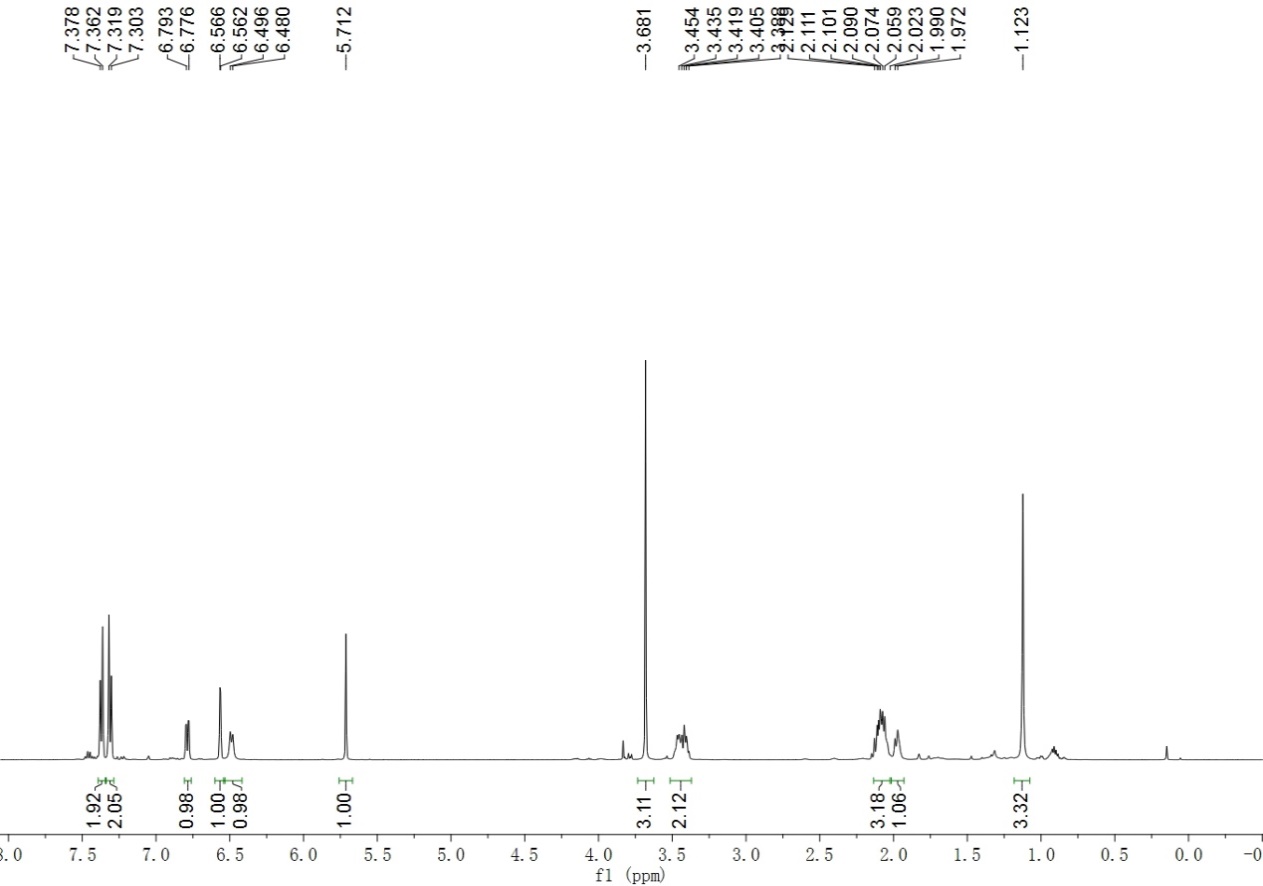


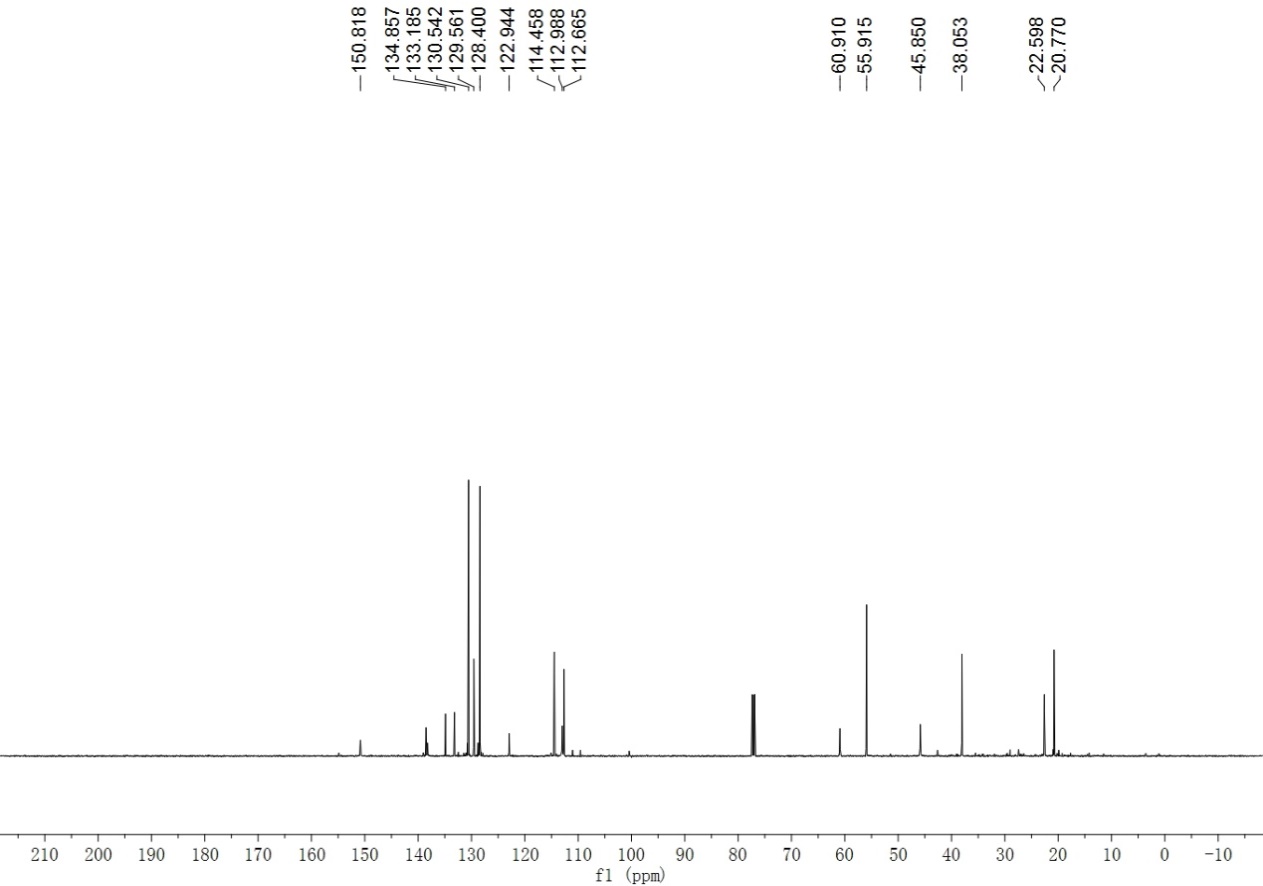


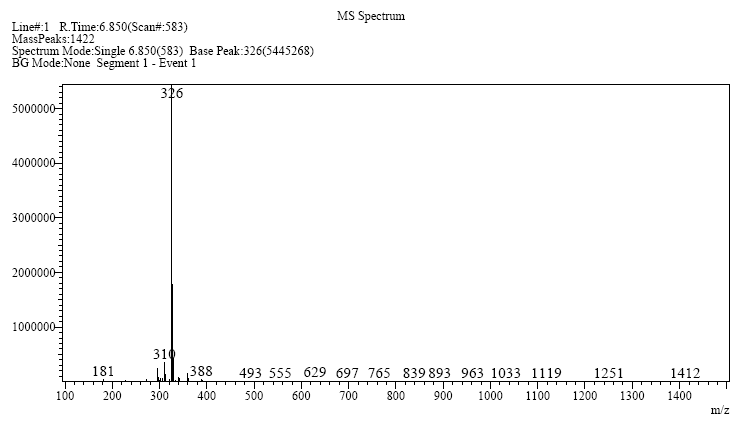


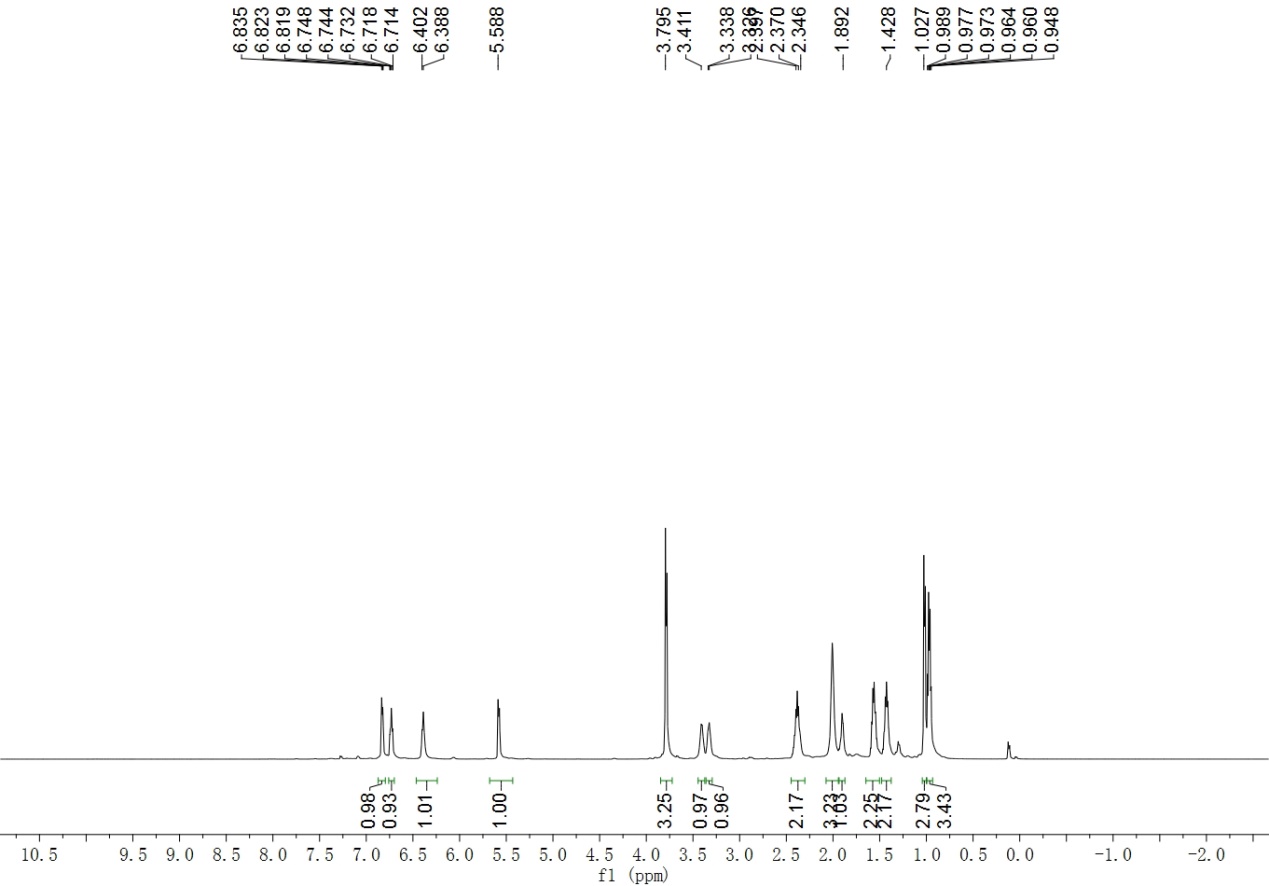


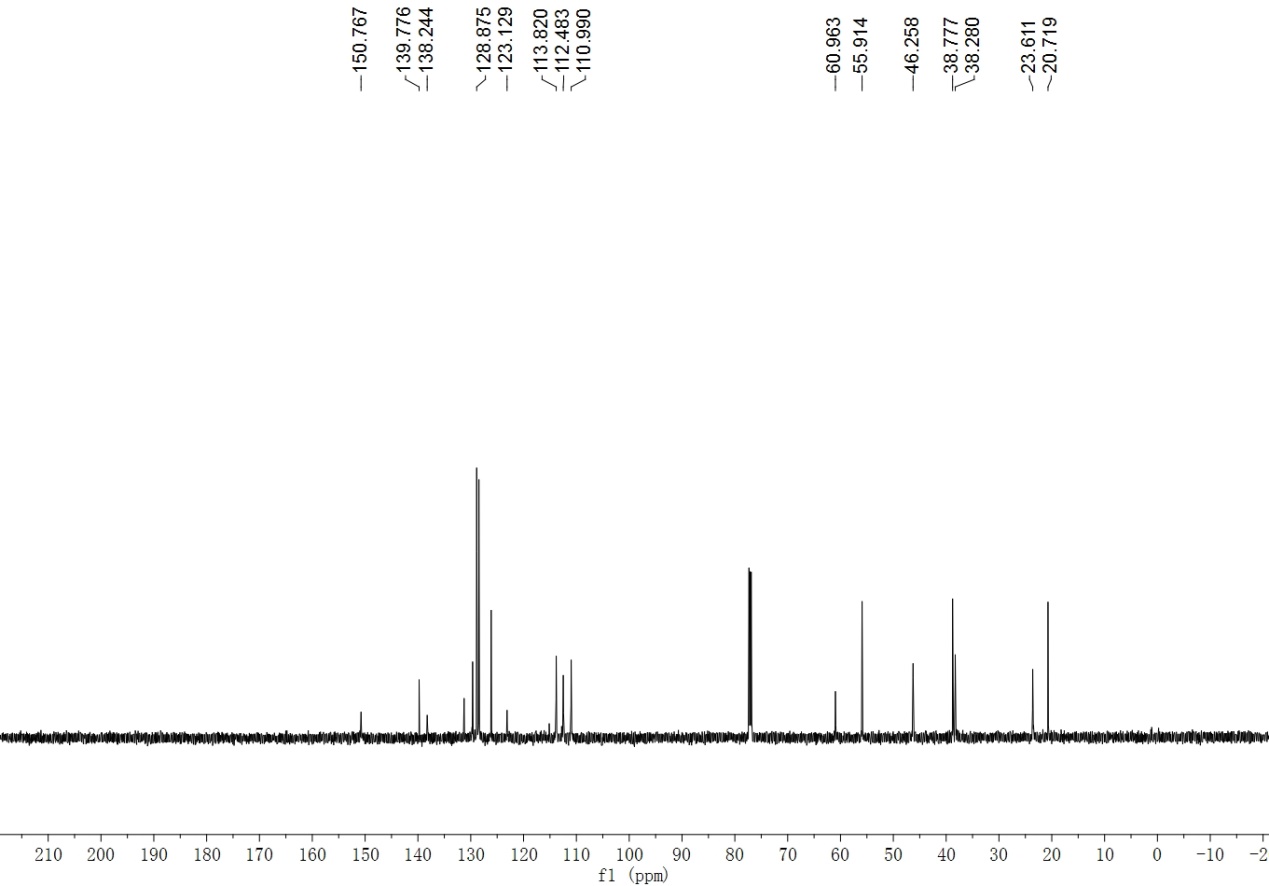


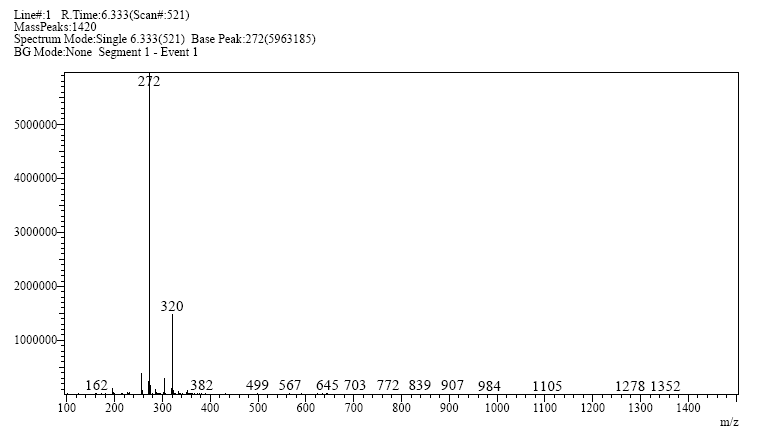


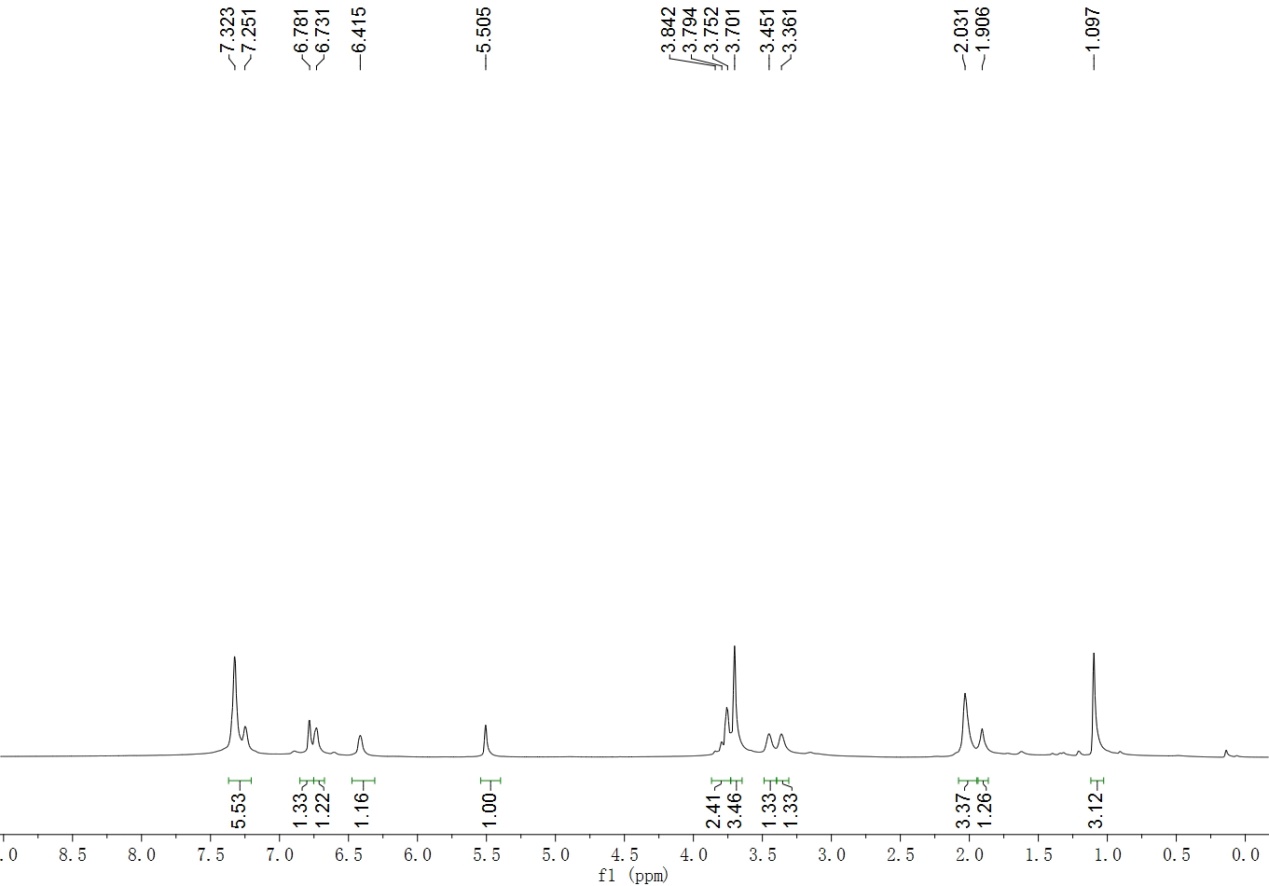


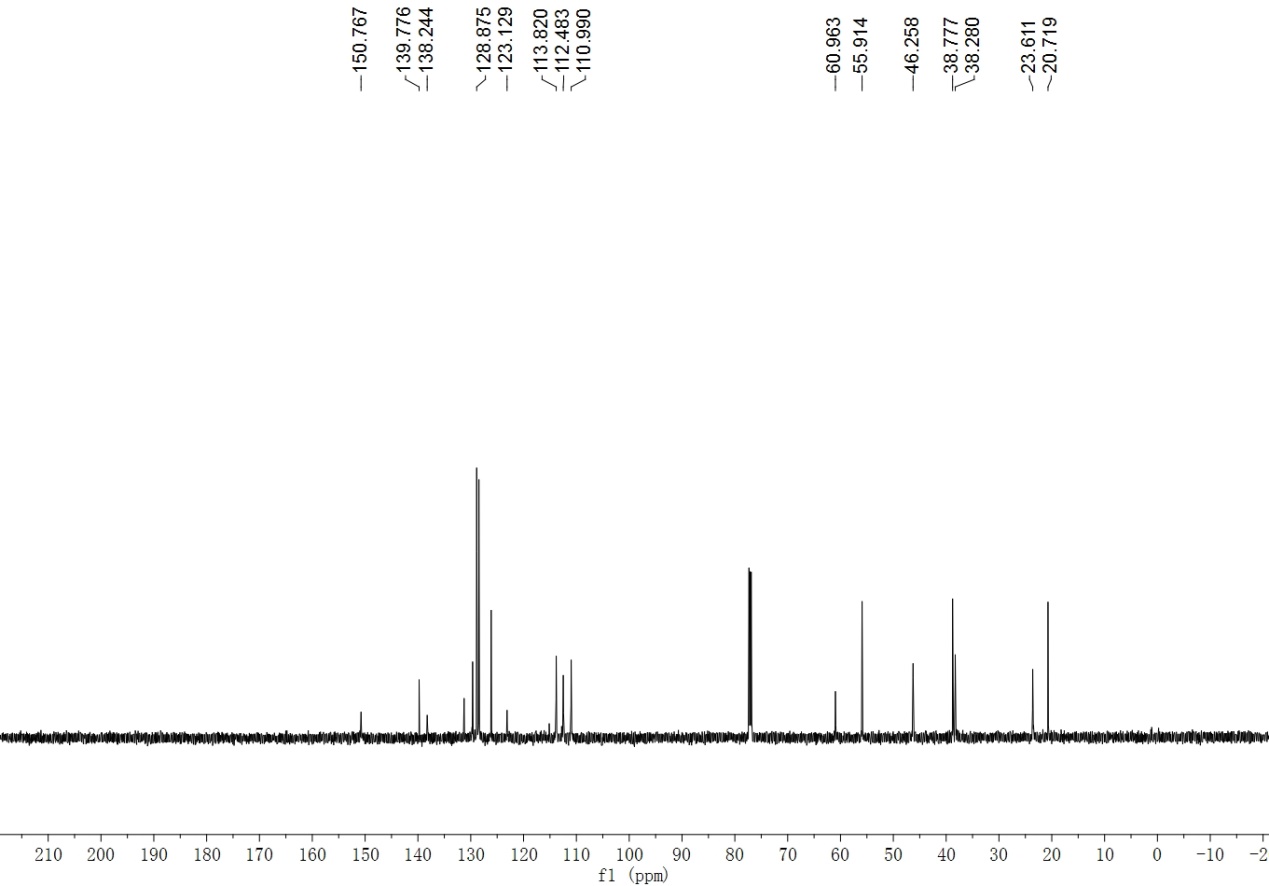


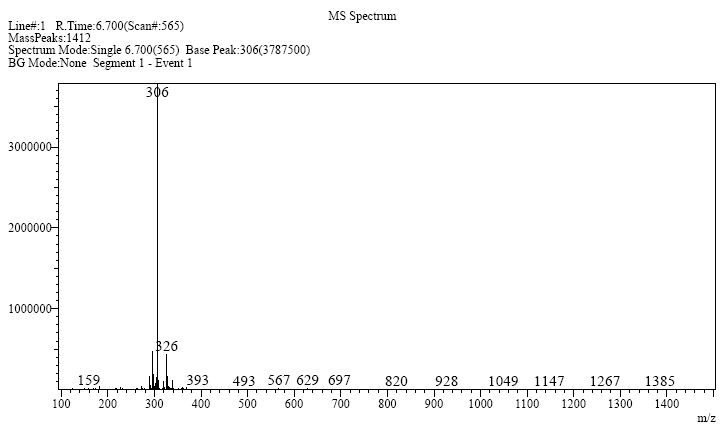


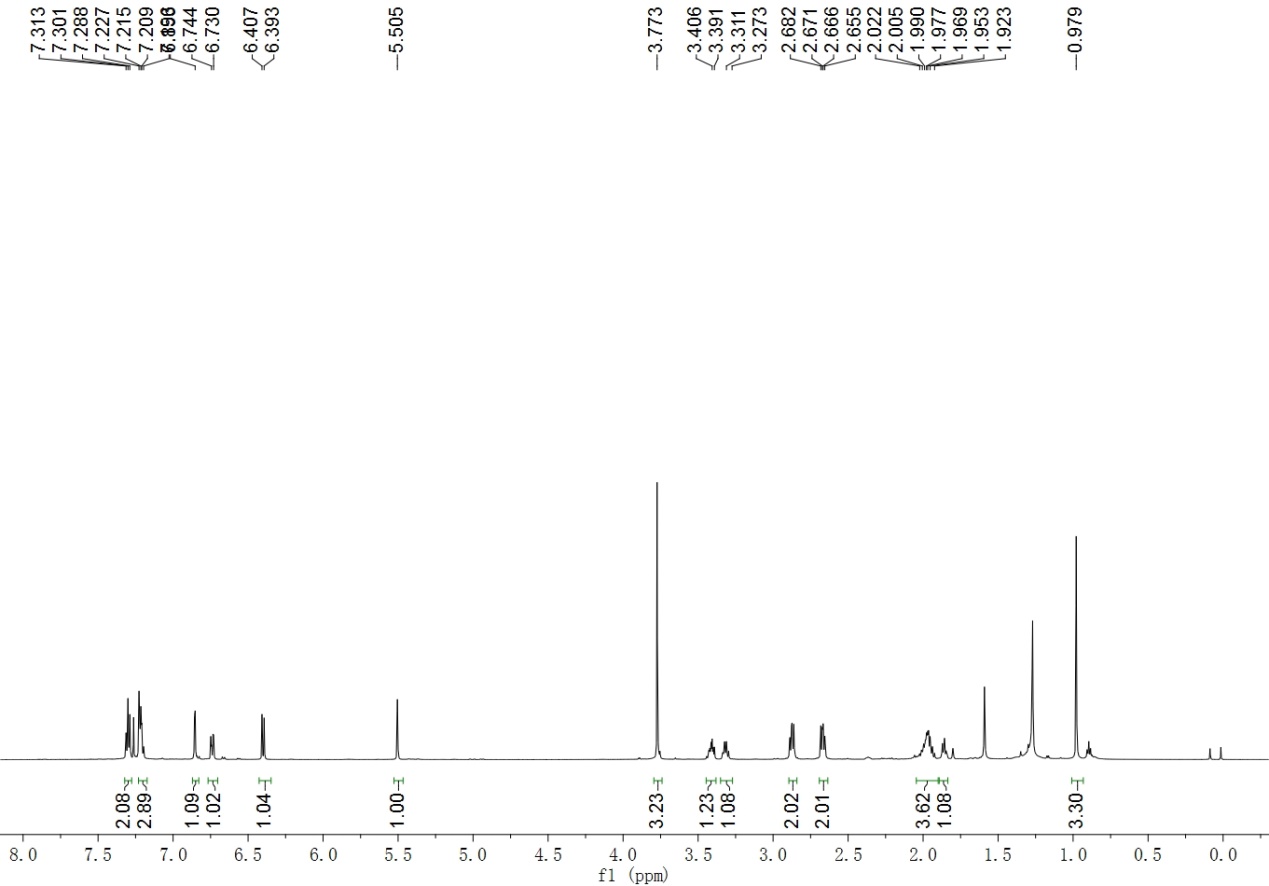

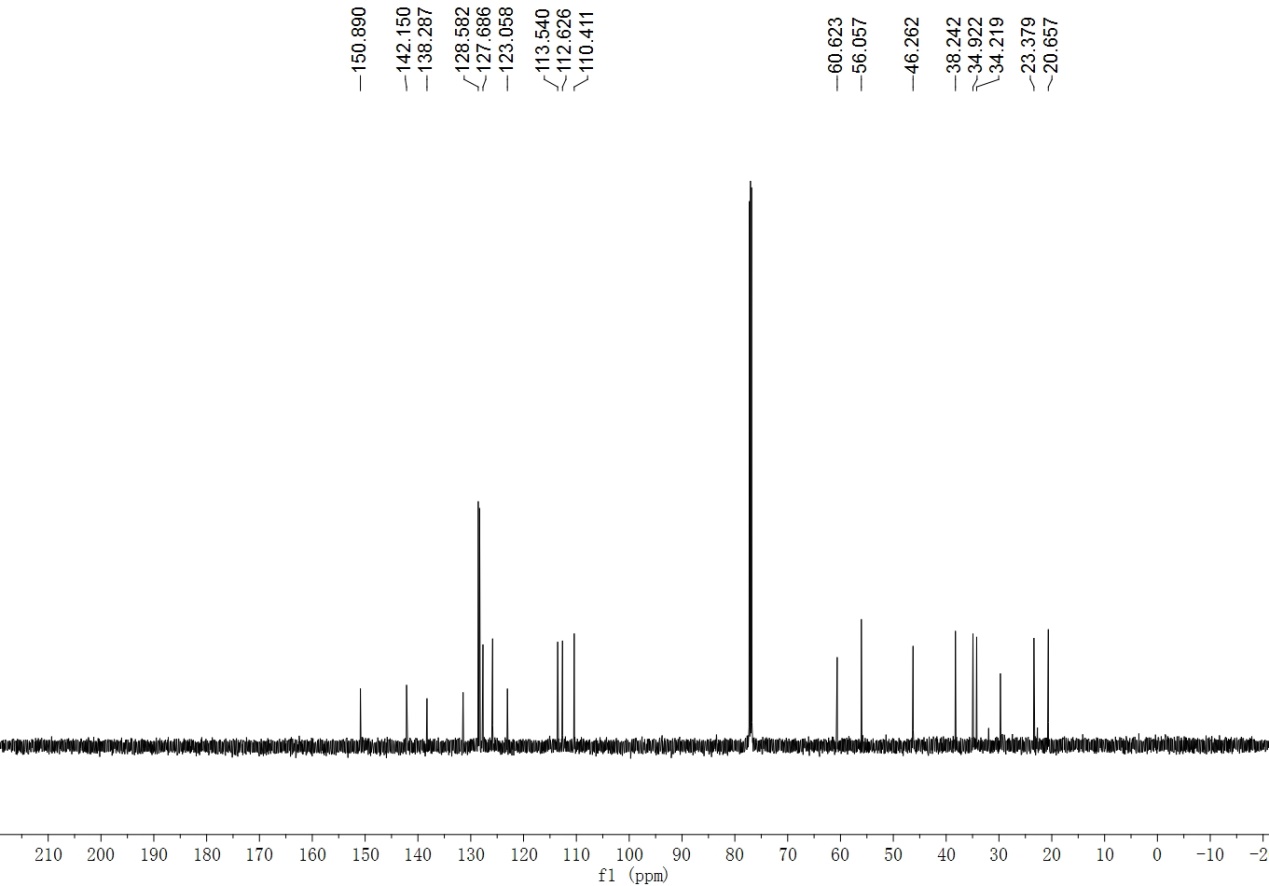


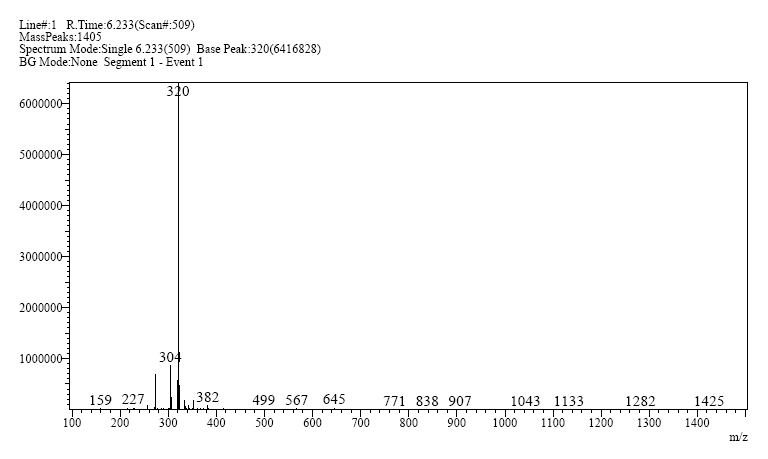


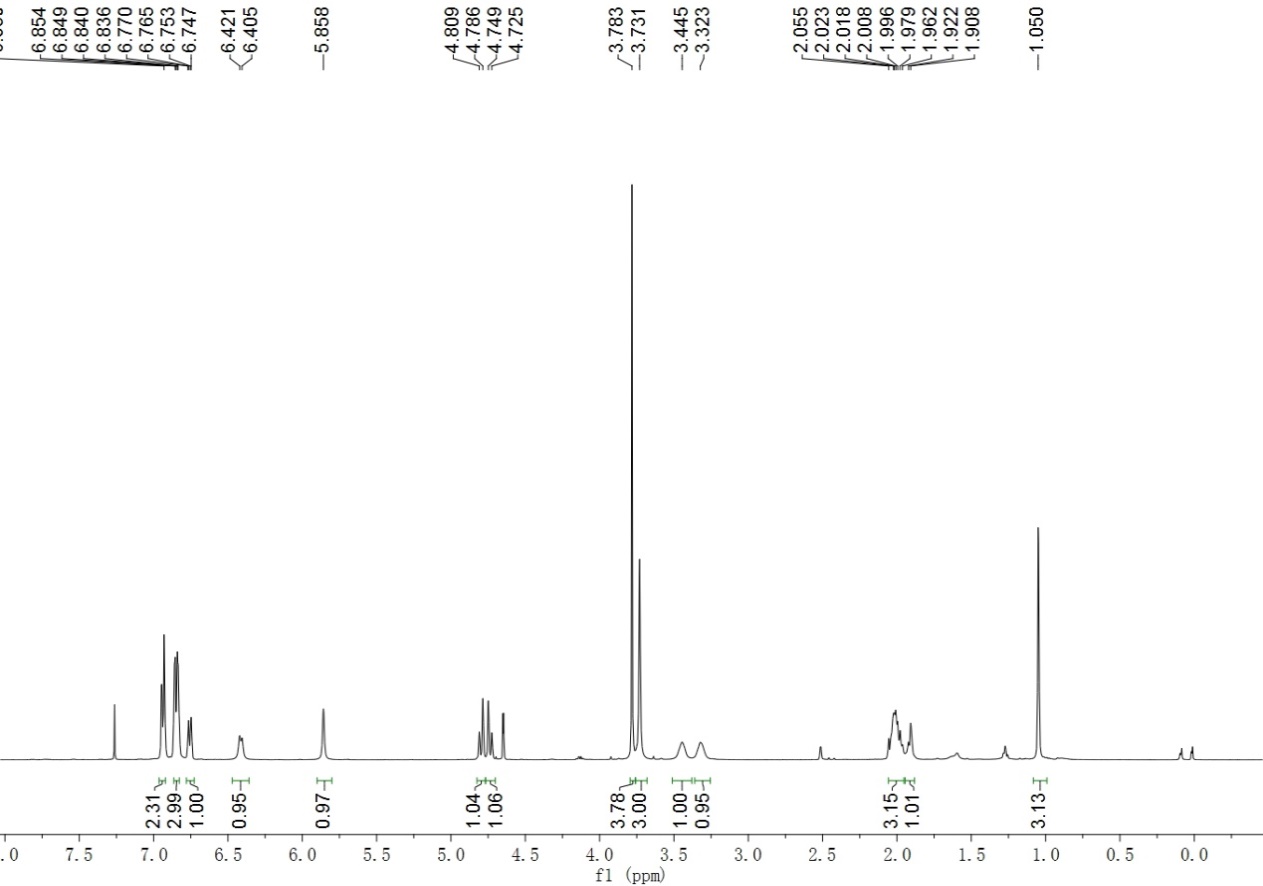


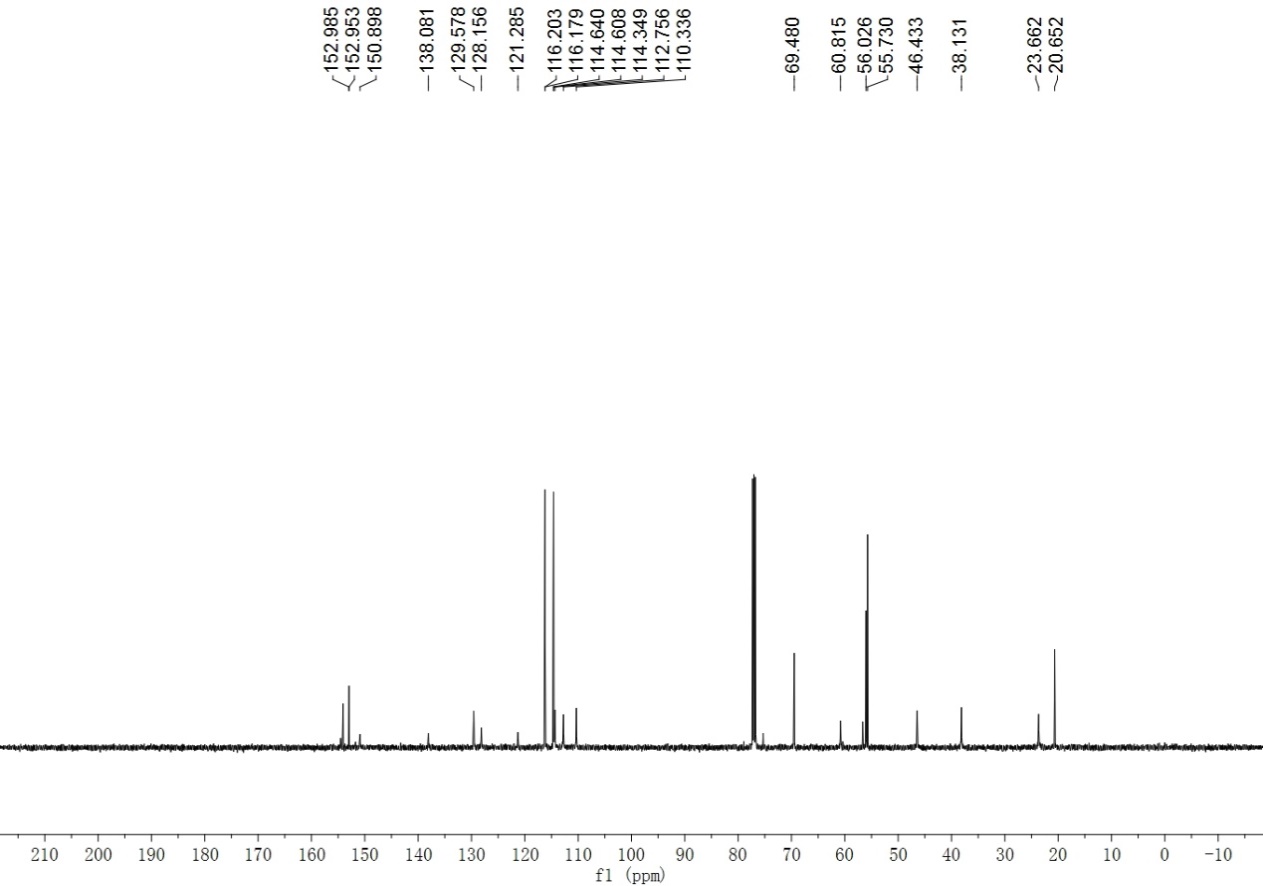


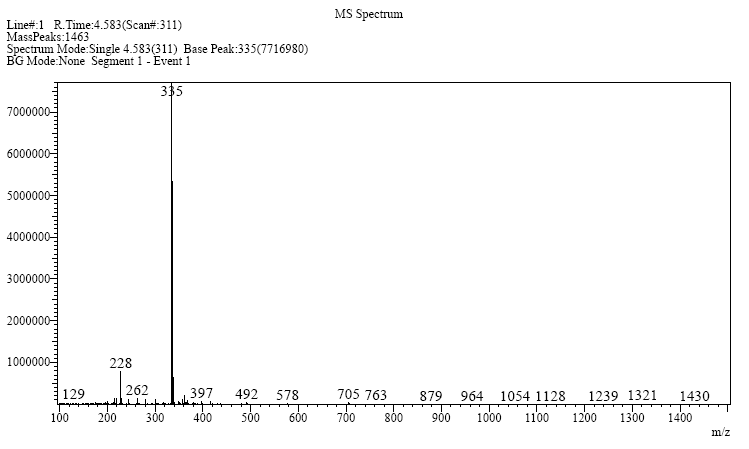


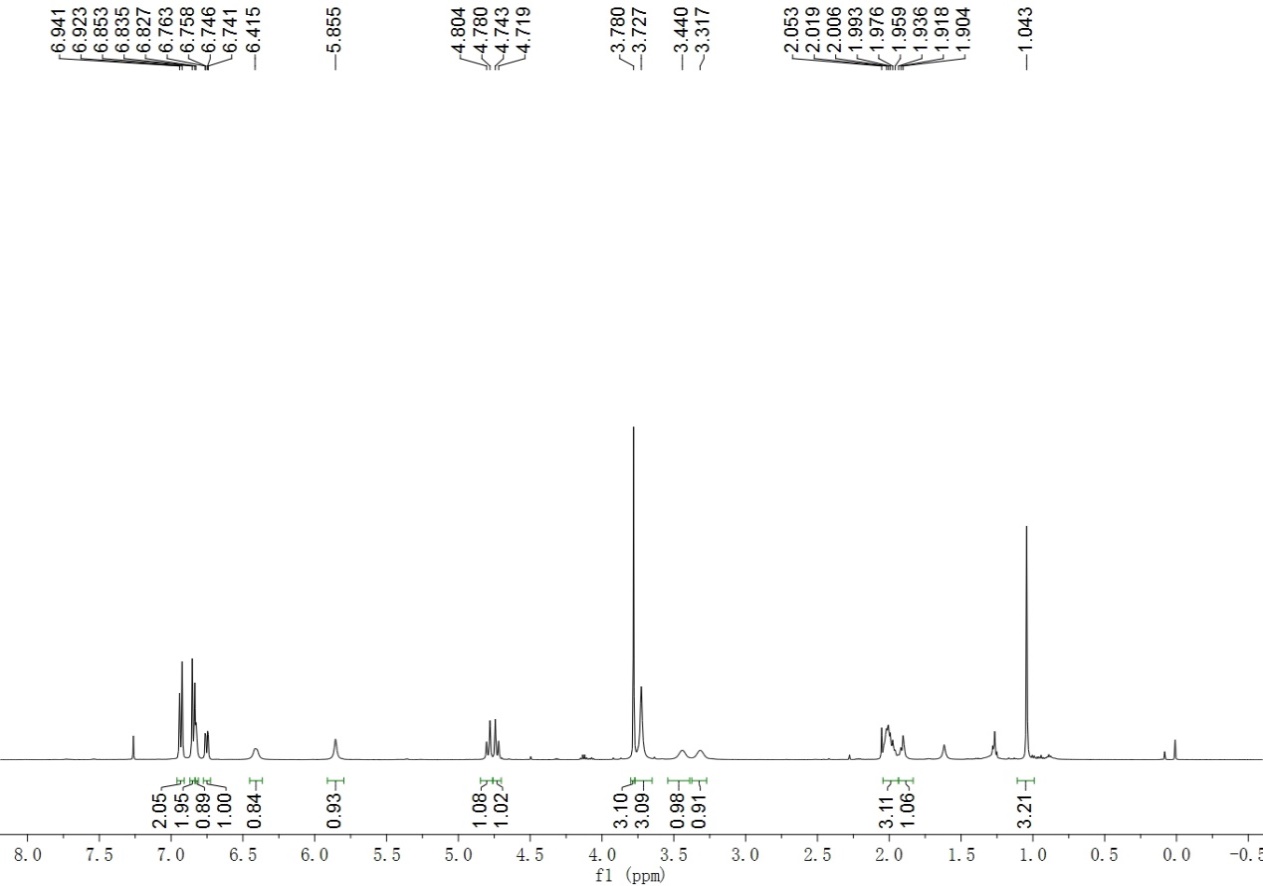


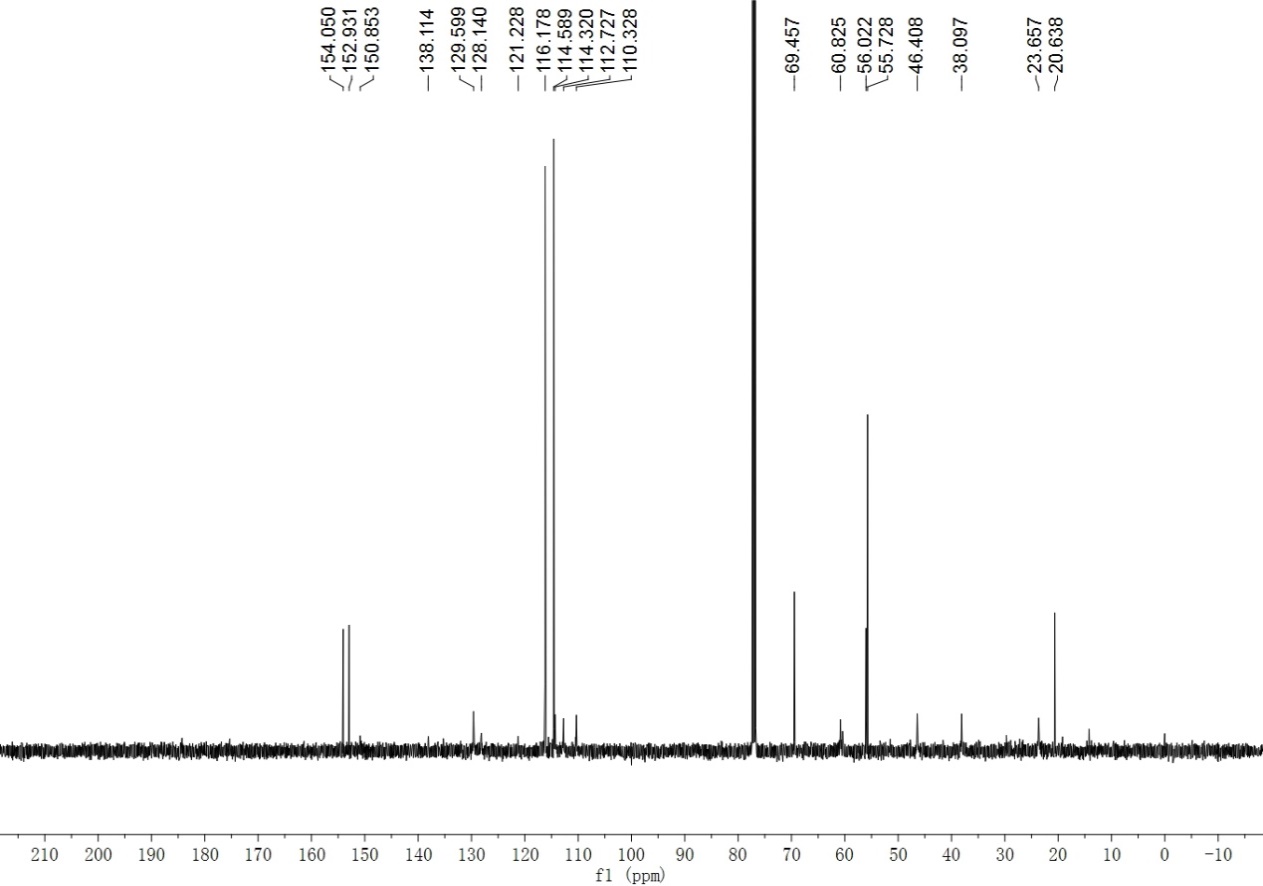


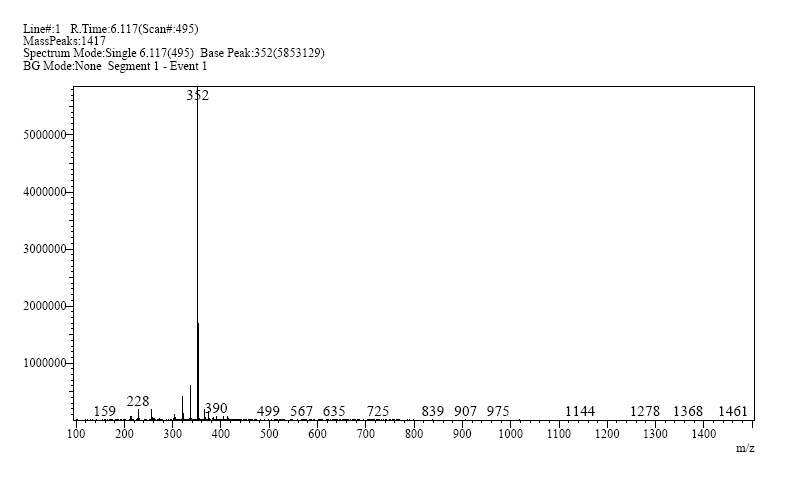


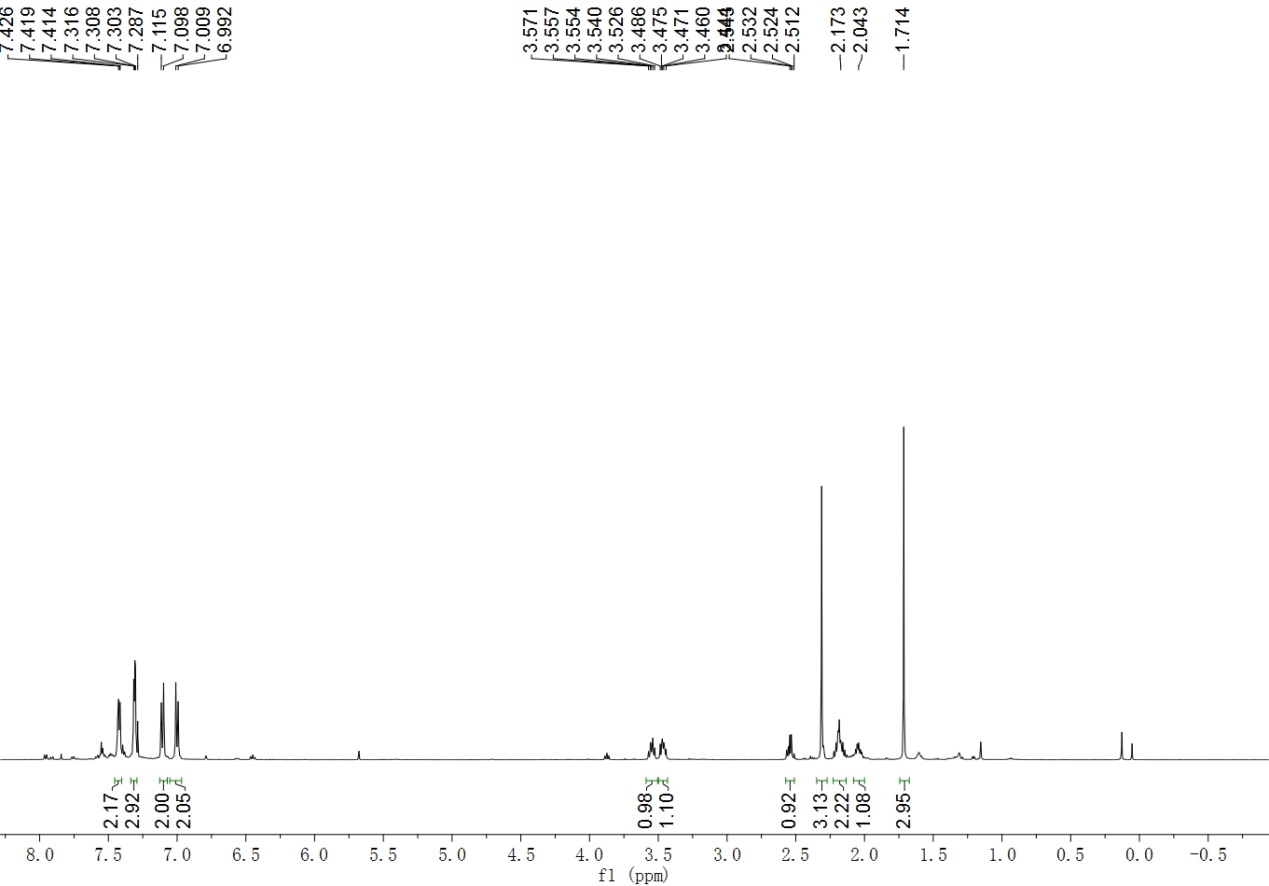


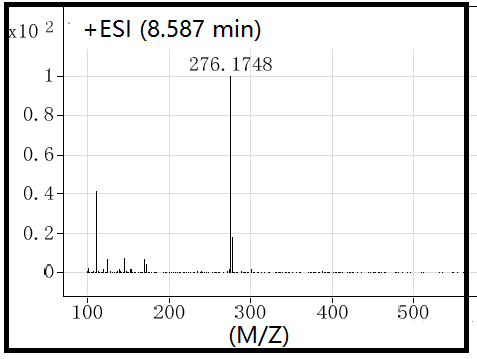

Supplement: Supplementary file 1 — Supplementary Information [file 41598_2017_16887_MOESM1_ESM.doc]
